# Magnetospectroscopic Studies of a Series of Fe(II) Scorpionate Complexes: Assessing the Relationship between Halide Identity and Zero-Field Splitting

**DOI:** 10.1021/acs.inorgchem.5c02691

**Published:** 2025-07-29

**Authors:** Daniel J. SantaLucia, Laxmi Devkota, Sergey V. Lindeman, Andrew Ozarowski, J. Krzystek, Mykhaylo Ozerov, Samuel M. Greer, Daniel C. Cummins, Klaus H. Theopold, Mihail Atanasov, Joshua Telser, Adam T. Fiedler

**Affiliations:** † 28313 Max Planck Institute for Chemical Energy Conversion, Stiftstraße 34-36, Mülheim an der Ruhr, North Rhine-Westphalia D-45470, Germany; ‡ Department of Chemistry, 5505 Marquette University, 1414 W Clybourn Street, Milwaukee, Wisconsin 53233, United States; § National High Magnetic Field Laboratory, Florida State University, 1800 E Paul Dirac Dr, Tallahassee, Florida 32310, United States; ∥ Department of Chemistry and Biochemistry, 7823 Florida State University, 95 Chieftain Way, Tallahassee, Florida 32306, United States; ⊥ Department of Chemistry and Biochemistry, 5972 University of Delaware, Brown Laboratory, Newark, Delaware 19716, United States; # Max-Planck-Institut für Kohlenforschung, Kaiser-Wilhelm-Platz 1, Mülheim an der Ruhr, North Rhine-Westphalia D-45470, Germany; ¶ Institute of General and Inorganic Chemistry, Bulgarian Academy of Sciences, Acad. Georgi Bonchev Street 11, Sofia BG-1113, Bulgaria; ∇ Department of Biological, Chemical and Physical Sciences, 2460 Roosevelt University, 430 S Michigan Avenue, Chicago, Illinois 60605, United States

## Abstract

Ferrous ions in four-coordinate environments are common in protein structures, synthetic catalysts, and molecular magnets. The 3*d* ^6^ configuration of high-spin Fe­(II) imparts an *S* = 2 ground state, whose analysis using conventional spectroscopic methods is often hindered by substantial zero-field splitting (ZFS). Herein, we provide detailed electronic-structure descriptions for [Fe^II^X­(Tp^*t*Bu,Me^)] (**1-X**; X = F, Cl, Br, I), where (Tp^*t*Bu,Me^)^−^ is hydrotris­(3-*tert*-butyl-5-methyl-pyrazol-1-yl)­borate. The three pyrazolyl N-donors of the “scorpionate” ligand facially coordinate to Fe­(II), giving idealized *C*_3v_ symmetry with the halide occupying the axial position. Although originally reported by Theopold and co-workers, this series is revisited herein using advanced experimental and theoretical tools. Ground-state transitions were probed by high-frequency and -field electron paramagnetic resonance (HFEPR) and far-infrared magnetic spectroscopy (FIRMS). Variable-temperature/-field (VTVH) ^57^Fe Mössbauer spectroscopy, paramagnetic susceptibility, and VTVH reduced magnetization were also utilized. This combined approach provided complete sets of spin-Hamiltonian parameters. Interpretation using ab initio multiconfigurational calculations enabled quantification of halide-dependent magnetoelectronic effects. Jahn–Teller distortions induce a descent in symmetry from *C*_3v_ to *C*_s_ in both solution and solid state. Finally, we demonstrate that the **1-X** series is ionic, with the ZFS arising from combined Jahn–Teller and ligand field effects, rather than intrinsic spin–orbit coupling from the halides.

## Graphical abstract

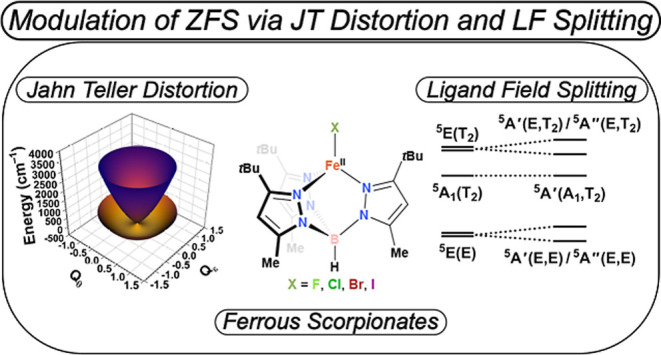

## 1 Introduction

In both synthetic and biological compounds, high-spin iron­(II) ions often adopt four-coordinate (4C) geometries that span between idealized tetrahedral (*T*_d_), trigonal pyramidal (*C*_3v_), and square planar (*D*_4h_) arrangements.^1–5^ The magnetic and spectroscopic properties of 4C ferrous centers are largely determined by their coordination environments, which modulate the relative energies of states arising from the high-spin *d* ^6^ (*S* = 2) configuration.^6^ Coordination geometry also plays a dominant role in adjusting biologically important Fe­(II/III) redox potentials, as well as regulating reactivity toward substrates in both homogeneous^7–9^ and enzymatic^10–12^ catalysis. Due to their integer-spin *S* = 2 ground state, high-spin Fe­(II) systems generally lack intense *d*–*d* transitions in their absorption spectra,^13^ and characterization with conventional (X-band) EPR is hindered due to sizable zero-field splitting (ZFS).^14^ Electronic-structure descriptions are further complicated by 3*d*-orbital degeneracy and Jahn–Teller effects in high-symmetry environments.^15,16^

Numerous 4C Fe­(II) complexes have been prepared using facially coordinating “scorpionate” chelates, including families of tris­(pyrazolyl)­borate (Tp^R1,R2^)^17–33^ and tris­(carbene)­borate (R^1^B­(R^2^Im)_3_)^34–41^ ligands. These complexes typically feature an anionic axial ligand, X, which lies along the 3-fold rotation axis established by the facially coordinating scorpionate framework, giving rise to neutrally charged complexes with local trigonal-pyramidal (*C*_3v_) geometries. Control over structural, electronic, and magnetic properties is achieved by modifying the axial ligand or substituents of the scorpionate rings. Tris­(pyrazolyl)­borate complexes have been reported with halide,^17–25^ alkylide/phenide,^17,18,26–28^ hydroxide/alkoxide/phenoxide,^17,19,29–31^ and bisulfide/thiolate^17,32,33^ axial ligands, while tris­(carbene)­borate complexes are limited to halide^34–38^ and phosphoraniminato^38–41^ axial ligands as well as one recent example of a phosphaethynolate^37^ complex. For example, Smith and co-workers demonstrated that a 4C tris­(carbene)-supported Fe­(II) complex with an axial phosphoraniminato ligand exhibits spin-crossover behavior and single-molecule magnetism at low temperature.^40,41^ Among the many reported [Fe^II^X­(Tp^R1,R2^)] complexes, in the current study we focus exclusively on the Tp-supported iron­(II)-halide complexes generated by Parkin, Theopold, and others.^17–25^ These 14-electron complexes maintain coordinative unsaturation through the presence of sterically bulky substituents (e.g., *tert*-butyl, mesityl, or isopropyl) at the 3-position of the pyrazolyl rings. Although [Fe^II^X­(Tp^R1,R2^)] (henceforth, X = halide ligand) complexes have been structurally characterized with X-ray diffraction (XRD), detailed analyses of their spectroscopic and magnetic properties have not been reported until now.

Recently, some of us examined the impact of halide variations on the ZFS of an analogous series of 4C high-spin (*S* = 3/2) Co­(II) complexes, [Co^II^X­(Tp^*t*Bu,Me^)] (X = F, Cl, Br, I), where (Tp^*t*Bu,Me^)^−^ = hydrotris­(3-*tert*-butyl-5-methyl-pyrazol-1-yl)­borate.^42^ This study concluded that the variations in ZFS across the series are due to changes in the energies of excited states due to ligand-field effects and not to changes in intrinsic spin-orbit coupling (SOC) of the halide ligands. In contrast, earlier studies of analogous series of 4C high-spin (*S* = 1) Ni­(II) complexes, [Ni^II^XTp] and [Ni^II^X­(Tp^Me,Me^)] (where (Tp^Me,Me^)^−^ = hydrotris­(3,5-dimethyl-pyrazol-1-yl)­borate), suggested that both ligand-field and SOC effects impact the size and magnitude of ZFS.^43,44^

Given the relevance of high-spin Fe­(II) centers to the fields of metalloenzyme catalysis and molecular magnetism, we pursued multifaceted electronic-structure studies of the [Fe^II^X­(Tp^*t*Bu,Me^)] series (**1-X** in Scheme 1; X = F, Cl, Br, I). We sought to assess the impact of the halide ligand and investigate the mechanism(s) that give rise to the ZFS in the **1-X** series. The spectroscopic inaccessibility and inherent complexity of the high-spin Fe­(II) ground-state wave function required the application of advanced spectroscopic techniques, including high-frequency and -field electron paramagnetic resonance (HFEPR), far-infrared magnetic spectroscopy (FIRMS), and variable-temperature/-field (VTVH) ^57^Fe Mössbauer spectroscopy. As described herein, these spectroscopic methods were combined with magnetometry to obtain fully consistent sets of *S* = 2 spin-Hamiltonian parameters for each member of the **1-X** series. Shifts in ZFS parameters across the halide series were interpreted with the assistance of ab initio multiconfigurational calculations. Furthermore, we examined whether the Jahn–Teller effect is responsible for distortions in molecular geometry away from *C*_3v_ to approximate *C*_s_ symmetry, as revealed in experimental X-ray crystallographic structures and near-infrared (NIR) absorption spectra. Collectively, the results offer an exceptionally detailed and comprehensive description of the electronic, structural, and magnetic features of high-spin Fe­(II) ions in distorted four-coordinate environments.

**Scheme 1:**
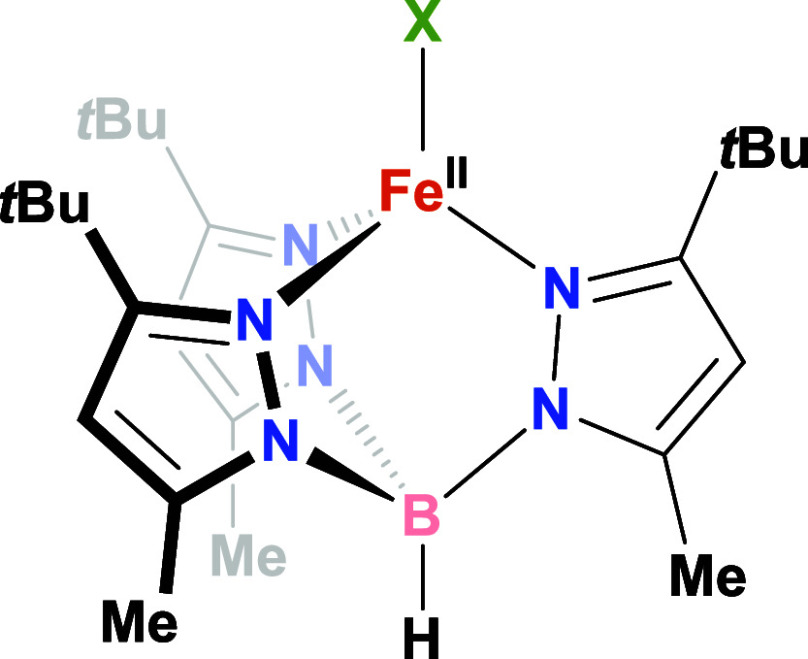
Idealized Structure of the **1-X** Series of Complexes (X = F, Cl, Br, or I) Studied Herein

## 2 Results and Analysis

### 2.1 Syntheses and X-ray Crystal Structures

Samples of [Fe^II^X­(Tp^*t*Bu,Me^)] (**1-X**; X = Cl, Br, I) were prepared following procedures derived from Theopold et al., with only minor modifications (see the General Synthetic Procedures section in the Supporting Information for details).^17^ In the earlier report, [Fe^II^F­(Tp^*t*Bu,Me^)] (**1-F**) was generated by treating **1-Cl** with AgBF_4_ in toluene; however, we found that using thallium­(I) fluoride instead provided higher yields and better purity (*Caution! Thallium salts are toxic and must be handled with care and appropriate PPE*). The identities of the **1-X** complexes were verified via single-crystal X-ray diffraction (SC-XRD) experiments (data collection and structural refinement parameters are reported in Tables S1 and S2). Crystals of **1-F**, **1-Cl**, and **1-Br** grown in our laboratory are isomorphous with those previously generated by Theopold et al.; in contrast, the unit cell parameters of **1-I** are unique. The structure of **1-Cl** reported herein exhibits 3-fold crystallographic symmetry; for completeness, we also analyzed another structure of **1-Cl** reported previously by Theopold et al., which is orthorhombic and has two-symmetry-independent molecules in its unit cell.^17^ For the **1-Br** and **1-I** complexes, two symmetry-independent molecules also are present in the unit cells.

Bond distances and angles derived from the X-ray structures are summarized in Tables S3–S5. In each case, the observed metric parameters are very similar to the values reported by Theopold and co-workers.^17^ As expected, the Fe–X bond distance increases with halide size, with average values (in Å) of 1.84 (**1-F**), 2.23 (**1-Cl**), 2.38 (**1-Br**), and 2.60 (**1-I**). The complexes exhibit either idealized *C*_3v_ symmetry (**1-Cl**) or approximate *C*_s_ symmetry (**1-F**, **1-Br**, and **1-I** as well as the previously reported^17^ **1-Cl** structure INUTUH01), with the 3-fold rotation axis defined along the B-Fe vector. The X–Fe–N_Tp_ bond angles fall within the range of 118–132°, whereas the smaller N_Tp_–Fe–N_Tp_ bond angles display an average value of 92.8[0.6]°.

### 2.2 Experimental and Computational Determination of Spin-Hamiltonian Parameters

#### 2.2.1 Magnetometry Studies

The paramagnetic susceptibility, χ_P_ (defined as χ_P_ = *M*/*H* for small applied fields, *H*),^45^ was determined for solid-state samples of each complex with a direct current applied field. Data measured for **1-F** and **1-I** are shown in Figure 1 while the data for **1-Cl** and **1-Br** are shown in Figure S1. The behavior of the paramagnetic susceptibilities (plotted as χ_P_ *T* vs *T*) across the **1-X** series is similar, with approximate level-off values in the high temperature ranges (>50 K) as well as steep downturns in χ_P_ *T* at low temperatures due to ZFS. The sharp downturns in χ_P_ *T* occur at lower temperatures in **1-Cl**, **1-Br**, and **1-I** (∼20 K) than in **1-F** (∼32 K). This difference suggests that the ZFS in **1-F** is larger than in **1-Cl**, **1-Br**, and **1-I**, as more thermal energy is required to significantly populate higher energy *M*_*S*_ levels in **1-F**. The small quasi-linear increase of χ_P_ *T* in the high temperature ranges of the paramagnetic susceptibility plots is due to temperature-independent paramagnetism (TIP),^45^ which is caused by second-order Zeeman coupling of the magnetic ground state to low-lying excited states (Van Vleck paramagnetism)^46^ and/or from small quantities of metallic impurities in the sample (Pauli paramagnetism).^47^ The TIP-corrected χ_P_ *T* values at 300 K for the **1-X** complexes range between values of 3.058 (**1-F**) and 3.279 (**1-Cl**) cm^3^ mol^–1^ K, which are slightly larger than the expected spin-only value of 3.008 cm^3^ mol^–1^ K for *S* = 2 systems. These deviations suggest that the *g*-values are larger than the free electron value (*g*_e_ = 2.002), consistent with the unquenched orbital angular momentum of the more-than-half-filled 3*d* ^6^ subshell of high-spin Fe­(II).^48^

**Figure 1:**
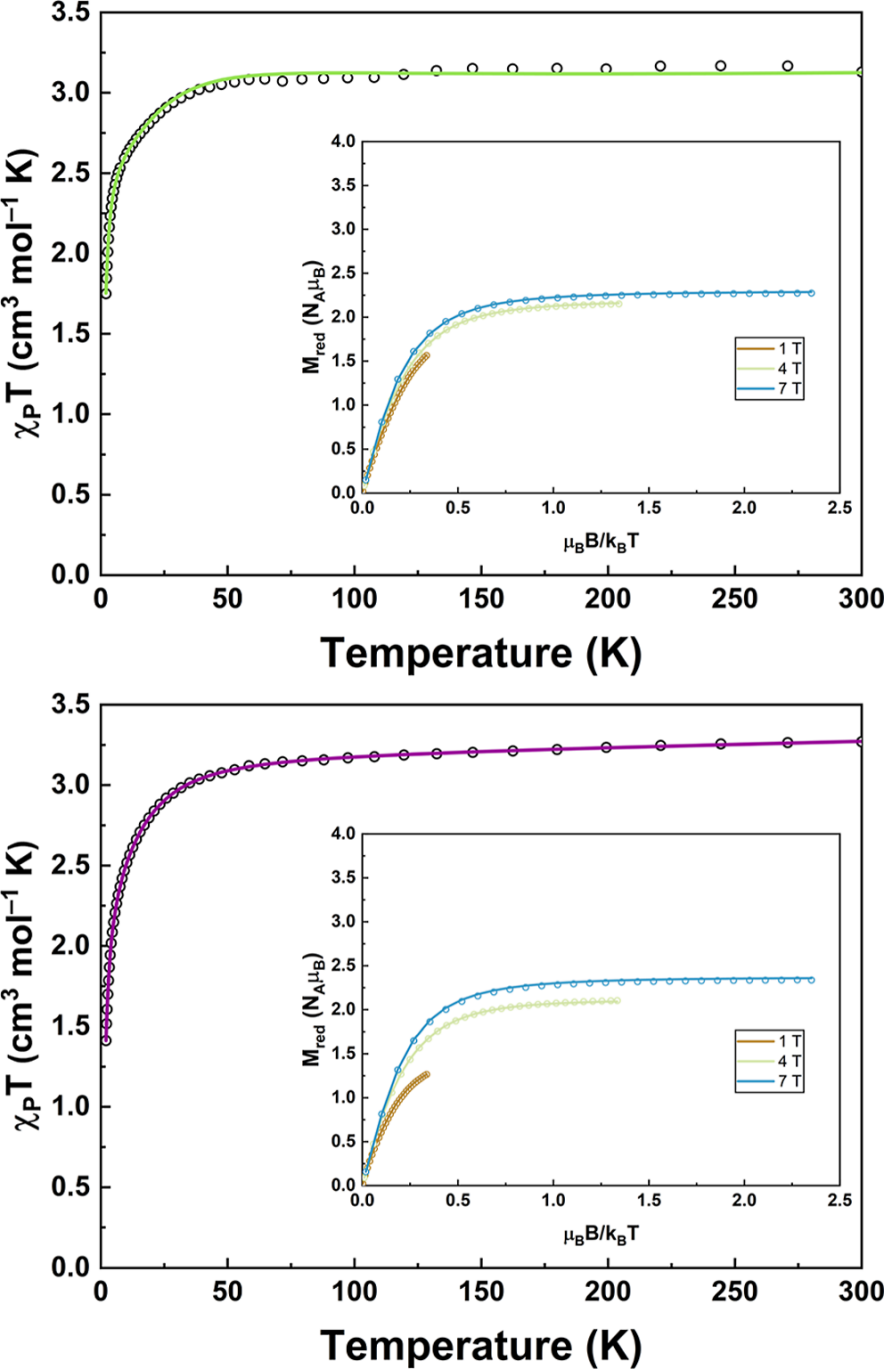
Paramagnetic susceptibility of **1**-**F** (top) and **1-I** (bottom) plotted as χ_P_ *T* vs *T* from 2 to 300 K. The insets display the corresponding VTVH reduced magnetization isofield measurements at 1, 4, and 7 T. The solid lines are the fits generated from the spin-Hamiltonian, where simultaneous fitting to both the susceptibility and VTVH data was performed. The fit parameters are reported in Table S6.

Variable-temperature/-field (VTVH) reduced magnetization data for each complex were measured at isofields of 1, 4, and 7 T, and reveal a similar conclusion about the relative sizes of the ZFS for each complex. The isofield traces for **1-F** are notably less nested than the isofield traces of the other **1-X** complexes (Figures 1 and S1, insets). Furthermore, the 7 T isofield trace levels off at a smaller value for **1-F** (*M*_red_/(*N*_A_μ_B_) = 2.274) than for **1-Cl**, **1-Br**, and **1-I** (2.593, 2.744, and 2.337, respectively) at high-fields and low-temperatures, which confirms that **1-F** possesses the largest ZFS of the series.^45^ The level-off values in the high reduced-field (μ_B_ *B*/*k*_B_ *T*) range suggest the following ordering for the magnitude of the ZFS: **1-F** > **1-I** > **1-Cl** > **1-Br**.^49,50^

The paramagnetic susceptibility and VTVH reduced magnetization data for the **1-X** complexes were modeled simultaneously using *S* = 2 spin-Hamiltonians with rhombic *g*-values and ZFS parameters. The exception was **1-F**, which was modeled with axial *g*-values and a D⃡ -tensor with only mild rhombicity. All fit parameters are reported in Table S6. The inclusion of the VTVH data in the fits allowed for the sign of the *D* parameters to be unambiguously determined for each complex.^49^ Since **1-Cl** was modeled at the rhombic limit with *E*/*D* = 0.333, the sign of *D* in this case is not meaningful, and the values of *g*_*y*_ & *g*_*z*_ may be swapped with one another upon changing the sign of *D*. The *D* parameter for **1-F** is indeed larger than the *D* parameters for the other **1-X** complexes, and the magnitudes of the *D* parameters follow the order of **1-F** > **1-I** > **1-Cl** > **1-Br**, consistent with the qualitative analysis of the VTVH data. Error sum plots of each fit to the data were generated for each complex by varying *D* and *E*/*D* (Figures S2–S5), which helped to validate the signs of the *D* values.

#### 2.2.2 HFEPR and FIRMS Studies

The presence of sizable ZFS in the **1-X** series, as revealed by magnetometry, prompted further investigation using far-infrared magnetic spectroscopy (FIRMS) in conjunction with high-frequency and -field electron paramagnetic resonance (HFEPR). FIRMS data were collected in the range of ∼10 to 120 cm^–1^ with applied fields up to 17.5 T, probing field-dependent transitions between *M*_*S*_ levels. HFEPR spectra were collected at 6–10 K with sub-THz frequencies (70–641 GHz) and applied fields up to 14.9 T. While HFEPR spectra were obtained for **1-Cl** and **1-Br**, no signals were detected for **1-F** and **1-I**. However, a perpendicular-mode X-band EPR spectrum (9.375 GHz) was successfully measured for **1-F** at 4 K (Figure S6). Two representative HFEPR spectra for **1-Br**, collected at different frequencies, are shown in Figure 2, while two representative HFEPR spectra for **1-Cl** are given in Figure S7.

**Figure 2:**
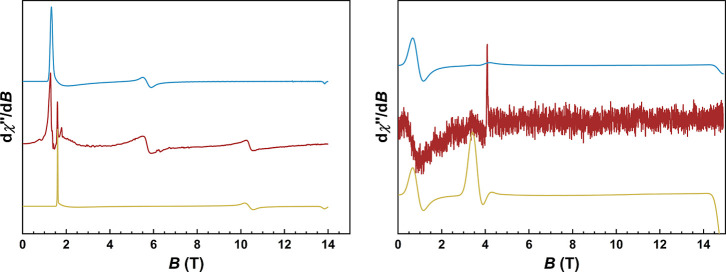
HFEPR spectra for **1-Br** collected at 6 K and 208 GHz (left) and at 10 K and 504 GHz (right). The experimental data are in maroon while spin-Hamiltonian simulations using positive and negative ZFS parameters are in blue and gold, respectively. The spin-Hamiltonian parameters are reported in Table S9.

FIRMS studies revealed numerous zero-field transitions and multiple field-dependent resonance modes for each complex of the **1-X** series. FIRMS spectra are shown in Figure S8 while the experimental transition energies observed at zero applied field in each FIRMS spectrum are given in Table S7. For an *S* = 2 system, up to ten zero-field transitions between the *M*_*S*_ levels are conceivable, with the following zero-field energies for the *M*_*S*_ levels (assuming a positive *D* parameter and neglecting the fourth-order ZFS terms *B*_4_^0^ and *B*_4_^3^)­

$$\begin{array}{l} |0,+2\rangle =\mp 2\rangle \sqrt{D^{2}+3E^{2}}\\ |\pm 1\rangle =-D\pm 3E\\ |-2\rangle =2D \end{array}$$

­(the labels for the *M*_*S*_ levels are inexact, as the eigenstates are mixed with rhombicity). Relative energies of the *M*_*S*_ levels and possible zero-field transitions are shown in Figure S9. At the axial limit, only Δ*M*_*S*_ = ±1 transitions are allowed. Rhombicity introduces mixing between the *M*_*S*_ levels, such that formally Δ*M*_*S*_ = ±2, ±3, and ±4 transitions become partially allowed; at the rhombic limit all transitions are allowed. Depending on the availability of HFEPR spectra for a particular complex, different approaches were used to simulate the FIRMS data of the **1-X** series.

HFEPR spectra for **1-Cl** and **1-Br** were simulated using an *S* = 2 spin-Hamiltonian (Figures S7 and 2, respectively). The turning point *g*-values from simulations of individual HFEPR spectra were assigned to *x*-, *y*-, or *z*-direction branches, and the resulting spin-Hamiltonian parameters are provided in Table S9. Simulating every experimental transition visible in the HFEPR spectra required separate simulations using negative and positive ZFS parameters. The presence of both sets of transitions in the HFEPR spectra suggests that the populations of the *M*_*S*_ levels do not follow the expected Boltzmann distribution. We excluded the possibility of thermalization effects due to low-energy vibrational modes given that the HFEPR spectra were collected at well-controlled and stable temperatures. Instead, we hypothesize that relaxation effects far beyond the scope of this paper are the origin of the non-Boltzmann distributions. The ZFS parameters are consistent with those obtained from magnetometry, and their signs are reported with those obtained from simulating the magnetometry and VTVH Mössbauer data (vide infra). (Inclusion of a fourth-order ZFS parameter, *B*_4_^0^, improved the simulation of the data, but the values were small enough that they may be safely ignored for the discussion herein). In Figure 3, the turning point branches derived from the HFEPR simulations are superimposed on the FIRMS data, indicating good agreement between the two data sets (FIRMS data without any superimposed turning point branches are displayed in Figure S8). Table S8 displays predicted transition energies calculated from the parameters given in Table S9.

**Figure 3:**
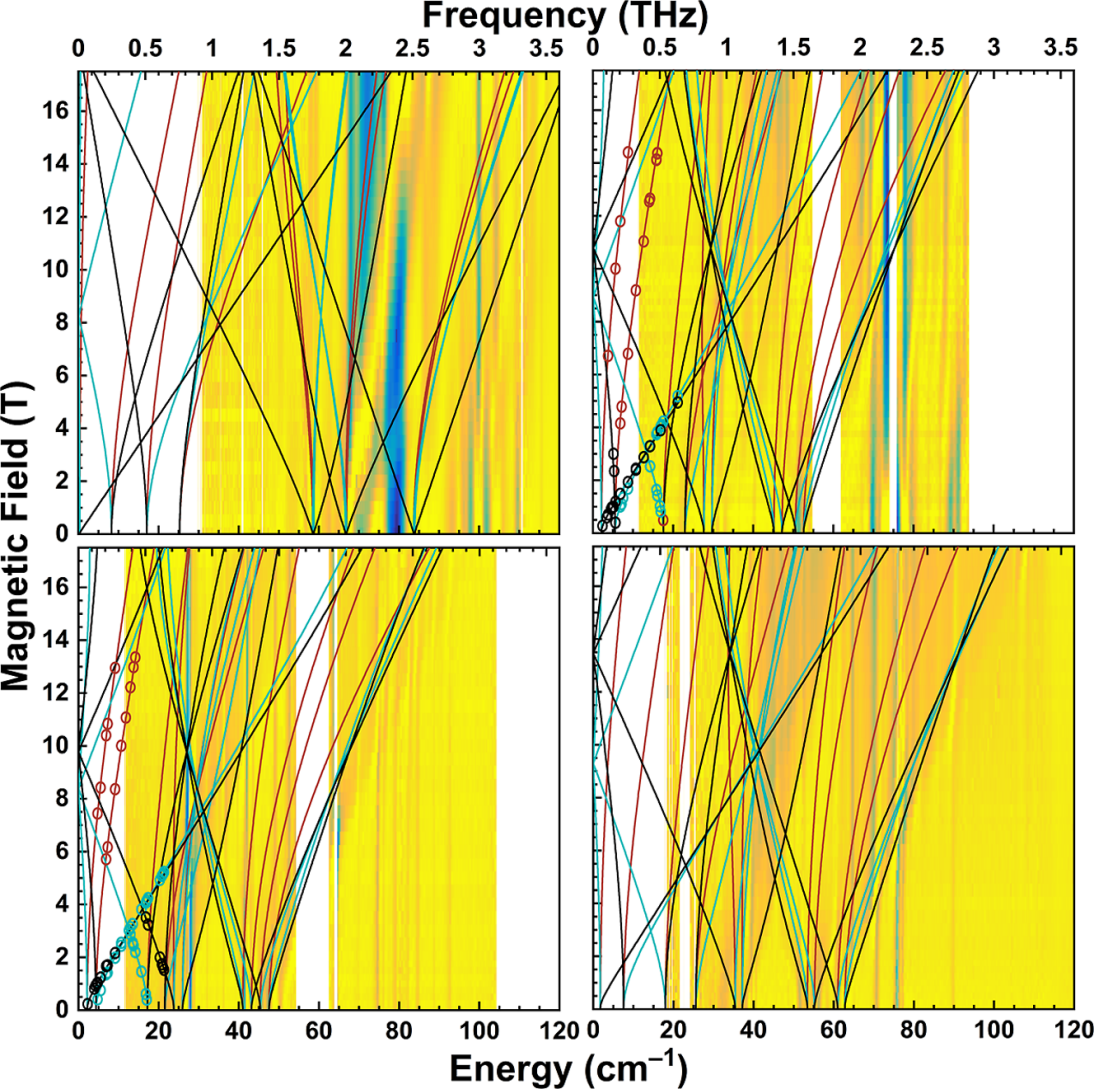
FIRMS data for the **1-X** series from 0 to 120 cm^–1^ as a function of applied magnetic field up to 17.5 T. Yellow and blue represent maximum and minimum transmittance, respectively. Top left: **1-F**; top right: **1-Cl**; bottom left: **1-Br**; and bottom right: **1-I**. Turning points from simulations to the HFEPR spectra for **1-Cl** and **1-Br** are included as open circles. Near vertical lines represent phonons and nonmagnetic vibrational modes while the energies of the magnetic dipole-allowed transitions exhibit field dependence. Turning point branches in the *x*-, *y*-, and *z*-directions (maroon, cyan, and black traces, respectively) were simulated based on the FIRMS data (**1-I**), a combination of both the HFEPR and FIRMS data (**1-Cl** and **1-Br**), or a combination of both the X-band EPR and FIRMS data (**1-F**). The simulated spin-Hamiltonian parameters are found in Table S9.

As HFEPR spectra were not available for **1-I**, the ZFS parameters in Table S9 were derived from simulating the turning point branches and comparing their agreement with the FIRMS data. The *g*-values obtained from magnetometry were used as fixed parameters, while the ZFS parameters were varied. This allowed for the calculation of the energies of the *M*_*S*_ levels; the Zeeman splitting of these levels was then calculated from 0 to 17.5 T (applied in the in the *x*-, *y*-, and *z*-directions). Finally, the energy differences between the *M*_*S*_ levels corresponding to the transitions in Figure S9 were calculated, yielding simulated turning point branches. This process was repeated until ZFS parameters were obtained that gave turning point branches that had good agreement when superimposed with the FIRMS data for **1-I**.

For the case of **1-F**, the X-band EPR spectrum (Figure S6) enabled precise determination of its *E*/*D* ratio by using the *g*-values and *D* parameter obtained from the magnetometry data as fixed values during simulation. The process of simulating turning point branches and comparing their agreement with the FIRMS data for **1-F** was then repeated by using the *g*-values obtained from magnetometry and the *E*/*D* ratio obtained from simulating the X-band EPR spectrum as fixed parameters, while varying only the *D* parameter until the turning point branches agreed well when superimposed with the FIRMS data.

#### 2.2.3 Mössbauer Spectroscopy

Zero-field Mössbauer spectra were collected at 1.6–1.8 K for each complex. From the spectrum of **1-F** (Figure 4, top), it is apparent that two quadrupole doublets are present in an approximately 1:1 ratio, suggesting that two species are present in the sample. In contrast, the single-crystal X-ray data show that there is only one molecule per unit cell, and the ^1^H NMR spectrum (available in the open-access Supporting Information) shows that the sample is composed of only one resolvable **1-F** species in solution at room temperature. We also collected powder X-ray diffraction data (Figure S10), which clearly shows that the sample comprised of only one crystalline phase. We therefore hypothesize that the two quadrupole doublets correspond to different chemical environments arising from Jahn–Teller distortions in the powder sample—a possibility discussed in greater detail below in the section on Vibronic Coupling and Jahn–Teller Analysis. Given the presence of two distinct quadrupole doublets, two subspectra are required to adequately model the zero-field Mössbauer spectrum. As shown in Figure S11 and Table 1, two possible fits were considered. The first fit considers the possibility that the isomer shifts (δ) of the quadrupole doublets differ while having similar quadrupole splittings (|Δ*E*_*Q*_|), whereas the second fit considers the alternative scenario with similar δ values and different |Δ*E*_*Q*_| values. While it is not possible to distinguish the correct fit on the basis of goodness-of-fit metrics to the data, the second possibility (fit 2 in Table 1) is more plausible because the other three **1-X** species exhibit isomer shifts in the 0.9–1.0 mm s^–1^ range. Furthermore, the changes in the chemical environments imparted by the two different geometries of the Jahn–Teller mode (vide infra) likely would lead to distinct lattice contributions to the electric field gradient (efg) tensors, producing distinct quadrupole splittings.^51^ Finally, only parameters from fit 2 led to reasonable fits of the VTVH Mössbauer data discussed below.

**Figure 4:**
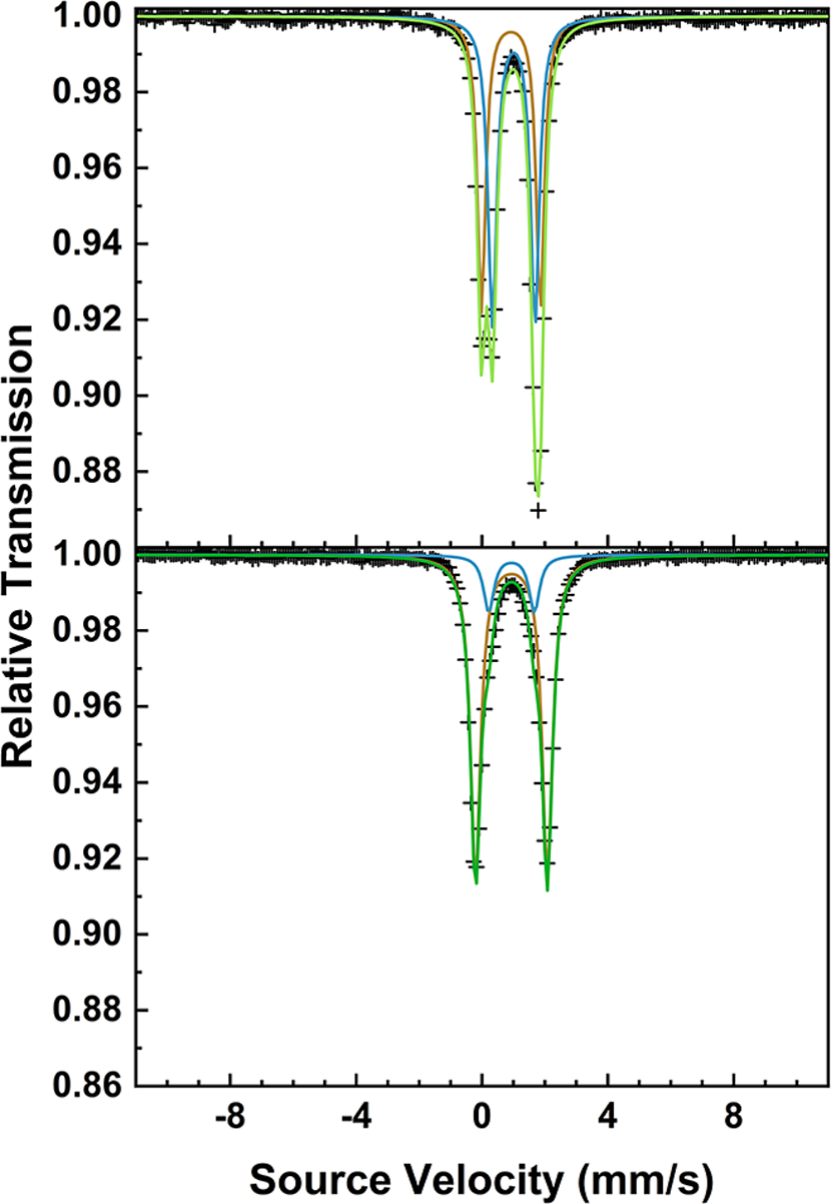
Zero-field Mössbauer spectrum of **1-F** (top) and zero-field (0.05 T) spectrum of **1-Cl** (bottom). The solid lines correspond to the spin-Hamiltonian model, where brown is the first subspectrum, blue the second subspectrum, yellow-green the overall fit (fit 2) to the data for **1-F**, and green the overall fit to the data for **1-Cl**. Fit parameters are reported in Table 1.

**Table 1:** Summary Fit Parameters to the Zero-Field Mössbauer Spectra for **1-X**

parameter	**1-F** (fit 1)	**1-F** (fit 2)	**1-Cl**	**1-Br**	**1-I**
δ_1_ (mm s^–1^)	0.830(8)	0.910(9)	0.930(9)	0.970(10)	0.930(9)
|Δ*E*_*Q*1_| (mm s^–1^)	1.69(5)	1.87(6)	2.28(7)	2.75(8)	2.77(8)
Γ_1_ (mm s^–1^)	0.340(17)	0.310(16)	*0.40*	0.350(18)	0.330(17)
*A*_1_ (%)	52.0(1.0)	46.8(9)	85.0(1.7)		
δ_2_ (mm s^–1^)	1.090(11)	1.000(10)	0.930(9)		
|Δ*E*_*Q*2_| (mm s^–1^)	1.52(5)	1.36(4)	1.45(4)		
Γ_2_ (mm s^–1^)	0.310(16)	0.340(17)	*0.40*		
*A*_2_ (%)	48.0(1.0)	53.2(1.1)	15.0(3)		

The near zero-field (0.05 T) Mössbauer spectrum for **1-Cl** collected at 1.8 K is displayed in Figure 4, bottom, along with the fit to the data. Similar to the zero-field spectrum for **1-F**, it is apparent that two quadrupole doublets are necessary to model the data, given the apparent shoulders visible at ∼0.2 and 1.6 mm s^–1^. Again, this result conflicts with the single-crystal X-ray and ^1^H NMR data, which suggest that there is only one **1-Cl** species. Therefore, as with **1-F**, we suggest that the two quadrupole doublets correspond to two distinct molecular geometries in the powder sample that arise due to Jahn–Teller distortions (vide infra). However, in the case of **1-Cl**, the intensities of the two quadrupole doublets are unequal, indicating that one geometric conformation is more favorable over the other. This major species contributes 85% of the Mössbauer intensity, compared to 15% for the minor species (Table 1).^52^ The near zero-field (0.05 T) Mössbauer spectra for **1-Br** and **1-I** collected at 1.7 K are remarkably similar to one another, as evident in Figure S12 and the fit results in Table 1. Interestingly, only one quadrupole doublet is observed for each complex, which is consistent with the ^1^H NMR data, but in contrast to the fact that the single-crystal X-ray data show that there are two molecules per unit cell for **1-Br** and **1-I**. We propose that only one distorted geometry is present in the powder samples.

As shown by the crystal structures, the Fe–N_Tp_ bond distances remain largely unchanged across the **1-X** series. However, a progressive change in bond-lengths is observed for the Fe–X bonds. Because the isomer shift is normally correlated to bond lengths (a proxy for covalency),^51^ the lack of change across the series suggests that the Fe–X bonds are highly ionic in character given the similarity in the isomer shifts observed across the **1-X** series (δ_avg_ = 0.95 ± 0.05 mm s^–1^). Similarly, this lack of covalency is also present in the analogous high-spin Co­(II) complexes that we studied previously.^42^

The magnitude of the quadrupole splittings changes across the **1-X** series with the following ordering of |Δ*E*_*Q*_|: **1-F** < **1-Cl** < **1-Br** = **1-I** (Table 1), implying that the electric field gradient increases systematically with Fe–X bond lengths until reaching a certain limit, exemplified by **1-Br** and **1-I**. Because the Fe–X bonds in the **1-X** series are quite ionic, it can be inferred that *V*_*zz*_ (the *z*-component of the efg) depends greatly on the proximity of a halide to its corresponding Fe center. However, beyond a certain distance, the impact of a given halide on the lattice contribution to *V*_*zz*_ more or less plateaus. This is unsurprising when one considers that *V*_*zz*_ ∝ *r* ^–3^, where *r* is the distance between the ^57^Fe nucleus and a hypothetical point charge.^51,53,54^ Altogether, the δ and |Δ*E*_*Q*_| parameters for each quadrupole doublet for each **1-X** species are consistent only with high-spin Fe­(II), showing that there are no Fe-containing impurities with other oxidation or spin-states.

The VTVH Mössbauer spectra for **1-F** are displayed in Figure 5, along with the fits to the data. Consistent with the spin-Hamiltonian parameters derived by magnetometry, it is clear that the *D* parameter is negative because of the observed rapid increase in magnetic hyperfine splitting upon application of an external magnetic field. The hyperfine splitting has nearly reached its maximum extent already at 1 T, suggesting a relatively large internal field $\frac{\left\langle -{\vec{S}}_{i}\right\rangle \mathrm{A⃡}}{g_{N}\mu_{N}}$ , which depends on the spin expectation values ⟨*S*⟩_*i*_ for each *M*_*S*_ level as well as the strength of the electron–nuclear hyperfine coupling tensor, A⃡ . This implies a distinct rapid change in the expectation value of the total spin, ⟨*S*⟩, consistent only with a negative value for the *D* parameter. This is demonstrated in Figure S13, which shows how the spin expectation values for each *M*_*S*_ level for **1-F** change; at 1.8 K and with a negative ZFS, the only levels with any appreciable Boltzmann population are *M*_*S*_ = ±2. Conversely, for a positive *D* parameter, it would be expected that ⟨*S*⟩ would not change much with an externally applied field since only the *M*_*S*_ = 0 sublevel would be populated at 1.8 K and, hence, the observed magnetic hyperfine splitting of the Mössbauer spectrum would be much smaller, depending only on the external applied field.^51^ This is exemplified by the VTVH Mössbauer data for **1-Br** (Figure S15), which clearly show a small magnetic hyperfine splitting even up to applied fields of 7 T. The fits to the data for **1-Br** reflect this, with the sign of *D* being clearly positive.

**Figure 5:**
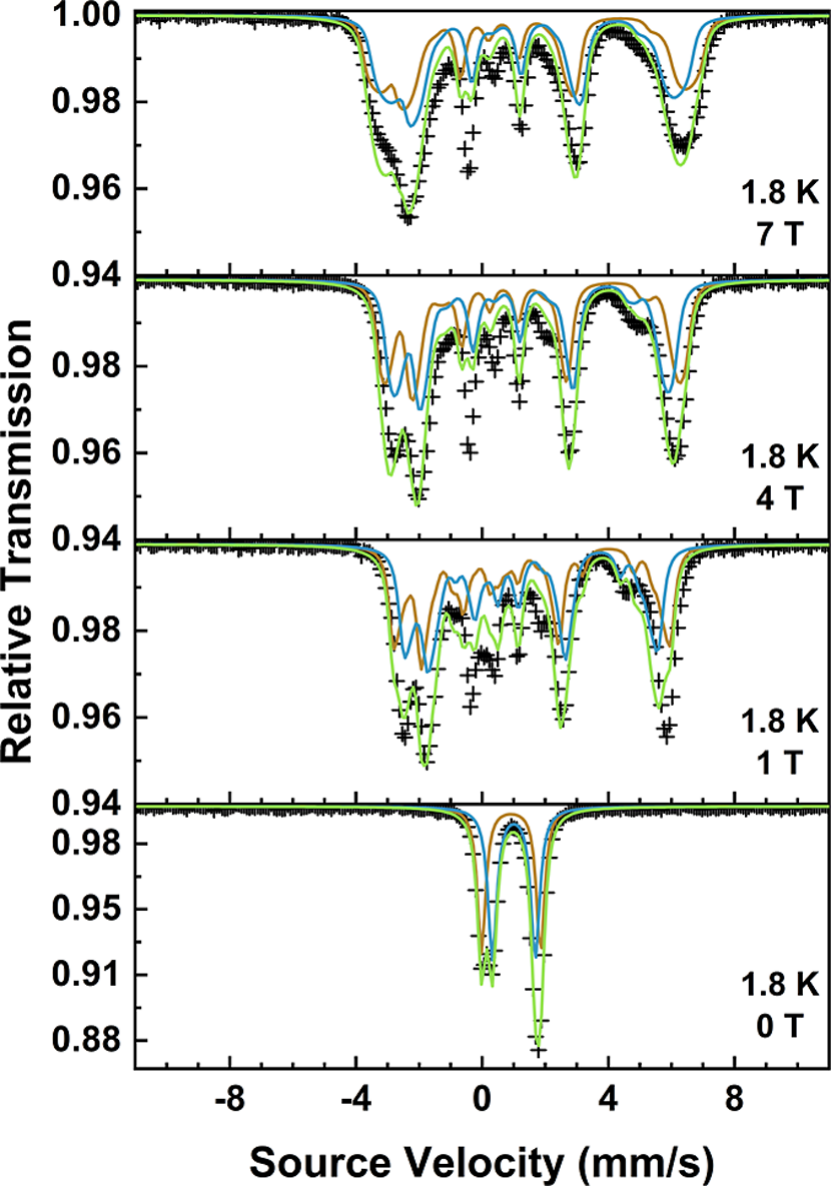
VTVH Mössbauer spectra of **1-F** with fit to the data including both subspectra from fit 2 of the zero-field spectrum (bottom). Bottom to top: 0, 1, 4, and 7 T, all at 1.8 K. The solid lines correspond to the spin-Hamiltonian model, where brown is the first subspectrum, blue the second subspectrum, and yellow-green the overall fit to the data. Fit parameters are reported in Table S10.

It should be noted that this argument is most applicable for systems with rhombicity parameters closer to the axial limit (*E*/D < 0.15), such as **1-F**. The other three systems in the **1-X** series are near the rhombic limit, so determination of the sign of *D* via inspection of the VTVH Mössbauer spectra is more difficult. However, consistent with the fits to the magnetometry data sets, inspection of the VTVH Mössbauer spectra for **1-Br** and **1-I** indicates that the *D* parameters are positive and negative, respectively, considering that the observed magnetic hyperfine splittings are relatively small and large, respectively, at small magnetic fields. The sign of *D* for **1-Cl** is irrelevant at the rhombic limit (vide supra), though it was modeled as positive and with a rhombicity not at the rhombic limit, consistent with the parameters obtained from modeling the HFEPR/FIRMS data. The VTVH Mössbauer data for **1-Cl**, **1-Br**, and **1-I**, as well as the fits to the data, are displayed in Figures S14–S16, respectively. The resulting spin-Hamiltonian fitting parameters are summarized in Table S10. Additional details concerning the fitting procedure for the VTVH Mössbauer data are provided in the Supporting Information, which includes Figure S17 and Table S11.

#### 2.2.4 Electronic Structure Calculations of Spin-Hamiltonian Parameters

Quantum chemical theory (QCT) was employed to assess the relationship between the coordination geometries and electronic structures for each **1-X** complex. Two separate sets of geometry optimizations for each complex were carried out at the level of density functional theory (DFT). In the first set, only the positions of the H atoms from the crystal structure were optimized (labeled “H-only”), whereas the positions of all atoms were optimized in the second set (labeled “Full Opt”). The H-only and Full Opt iron–ligand bond distances and angles are reported in Table S12 (note that the distances and angles for the H-only geometries are exactly equivalent to those of the crystal structure data). Given the crystallographically imposed trigonal symmetry for the **1-Cl** structure reported herein, we also considered the H-only optimized geometries for a previously reported orthorhombic structure of **1-Cl** (INUTUH01); both symmetry-independent molecules in its unit cell were considered.^17^ Similarly, both molecules in the crystallographic unit cells were considered for the H-only optimized geometries for **1-Br** and **1-I**. In full optimizations of **1-F**, two geometries with roughly equal energies were located, referred to as the forward (+) and backward (−) bent distortions (defined in the Vibronic Coupling and Jahn–Teller Analysis section below). For all other complexes, only the (+) bent distortion energy minimum was located.

We then turned to multireference calculations to probe the electronic structures of the **1-X** series. We utilized the state-averaged complete active space self-consistent field (SA-CASSCF) method with an active space consisting of six electrons in five Fe 3*d*-orbitals (i.e., CAS­(6,5)). Dynamic correlation was accounted for using strongly contracted *N*-electron valence state second-order perturbation theory (SC-NEVPT2). We then used the effective Hamiltonian approach to extract spin-Hamiltonian parameters for the **1-X** series.^55^ Both sets of geometries (i.e., H-only and Full Opt) were considered in our analyses for completeness and to minimize biasing the results toward a specific geometry. A complete comparison of experimental and computed spin-Hamiltonian parameters is reported in Table 2.

**Table 2:** Comparison of Spin-Hamiltonian Parameters for the **1-X** Series Obtained from Various Experimental and Computational Methods^a^

complex	method	*g*_*x*_	*g*_*y*_	*g*_*z*_	*g*_avg_ ^d^	*D* (cm^–1^)	*E*/*D*
**1-F**	magnetometry	2.028	2.028	2.394	2.157	–20.8	0.000–0.105
	X-Band EPR/FIRMS					–19.8	0.0655
	VTVH ^57^Fe Mössbauer					–	0.10 and 0.11
	CASSCF/NEVPT2 (H-only)	2.034	2.004	2.447	2.171	–23.19	0.049
	CASSCF/NEVPT2 (Full Opt (−))	2.015	2.047	2.346	2.141	–17.33	0.068
	CASSCF/NEVPT2 (Full Opt (+))	2.053	2.006	2.360	2.145	–18.10	0.078
**1-Cl**	magnetometry	2.002	2.235^c^	2.002^c^	2.083	±11.39	0.333
	HFEPR/FIRMS	2.159	2.248	2.337	2.249	+12.12	0.2406
	VTVH ^57^Fe Mössbauer					+	
	CASSCF/NEVPT2 (H-only)	1.746	1.725	2.585	2.058	–38.28	0.154
	CASSCF/NEVPT2 (H-only Mol. A)^b^	2.002	2.125	2.358	2.167	–15.48	0.301
	CASSCF/NEVPT2 (H-only Mol. B)^b^	2.010	2.160	2.261	2.146	+11.95	0.217
	CASSCF/NEVPT2 (Full Opt (+))	2.134	1.997	2.345	2.163	–14.74	0.318
**1-Br**	magnetometry	2.031	2.386	2.016	2.151	+8.28	0.255
	HFEPR/FIRMS	2.238	2.257	2.22	2.238	+10.741	0.2656
	VTVH ^57^Fe Mössbauer					+	
	CASSCF/NEVPT2 (H-only Mol. A)	2.167	2.015	2.258	2.149	+11.42	0.239
	CASSCF/NEVPT2 (H-only Mol. B)	2.168	2.018	2.261	2.151	+11.27	0.287
	CASSCF/NEVPT2 (Full Opt (+))	2.001	2.194	2.299	2.168	+15.86	0.134
**1-I**	magnetometry	2.010	2.381	2.306	2.238	–12.29	0.265
	FIRMS					–14.8	0.20
	VTVH ^57^Fe Mössbauer					–	
	CASSCF/NEVPT2 (H-only Mol. A)	2.352	1.996	2.142	2.168	–14.84	0.278
	CASSCF/NEVPT2 (H-only Mol. B)	2.369	2.012	2.119	2.172	–16.53	0.177
	CASSCF/NEVPT2 (Full Opt (+))	2.022	2.324	2.179	2.179	+17.19	0.271

a The (+) and (−) labels correspond to the forward and backward distortions, respectively, for the Full Opt geometries (defined in the Vibronic Coupling and Jahn Teller Analysis section below).

b H-only geometries from a previously reported orthorhombic structure (INUTUH01) were also considered for **1-Cl**.^17^

c *g*_*y*_ and *g*_*z*_ swap with one another upon changing the sign of *D* at the rhombic limit.

d $g_{avg}=\sqrt{\frac{\left({g_{x}}^{2}+{g_{y}}^{2}+{g_{z}}^{2}\right)}{3}}$ , which is the powder average.

One point that is readily apparent from the results reported in Table 2 is that the agreement of the CASSCF/NEVPT2 calculations with the experimentally determined spin-Hamiltonian parameters is strongly dependent on geometry. This fact is clearly seen with **1-Cl**, where the predicted spin-Hamiltonian parameters from the H-only structure derived from the X-ray data reported herein have very poor agreement, while the predicted spin-Hamiltonian parameters from the Full Opt (+) geometry are in relatively good agreement.^56^ Conversely, the parameters obtained from the Full Opt (+) geometries for **1-Br** and **1-I** are in worse agreement with the experimental values, with the computed parameters even predicting the incorrect sign of *D* for **1-I**. Instead, the parameters from the H-only geometries are in better agreement. For **1-F**, the parameters obtained from every geometry are in good agreement with the experimental results. We stress therefore that the ability to predict spin-Hamiltonian parameters with high-quality ab initio methods such as CASSCF/NEVPT2 is often very sensitive to geometric structure, *with no clear indication that either a fully optimized or crystallographic structure will lead to better results*.

We next sought to provide an explanation of the experimental zero-field splittings (ZFS) modeled for each complex. For transition metals, the primary contribution to ZFS is via spin–orbit coupling (SOC) between the ground and low-lying excited states, with multiplicities the same as or differing by ±1 relative to the ground state multiplicity.^57^ Using the NEVPT2-corrected SA-CASSCF transition energies between the quintet CASSCF states, in conjunction with the reported contributions of each individual root to the D⃡ tensor in the effective Hamiltonian approach (Tables S13–S16), we were able to delineate the contributions of each of the quintet states to the ZFS.^58^ Figure 6 shows the contributions of individual quintet states to the computed ZFS parameters of the Full Opt structures (the corresponding diagram for the H-only structures is provided in Figure S18). We also accounted for any spin–spin coupling (SSC) contributions, and found that they were ∼0.2–0.3 cm^–1^, an order of magnitude smaller than the SOC contributions (Table S17); they are therefore ignored herein given their relative unimportance. Calculated effective one-electron SOC constants (ζ) for the **1-X** series are reported in Table S18.

**Figure 6:**
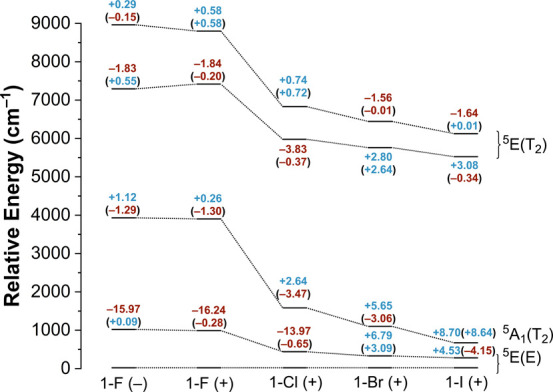
Energies of the five quintet states for each of the **1-X** complexes derived from the CASSCF/NEVPT2 calculations on the Full Opt geometries. The state labels from *C*_3v_ symmetry are used merely for accounting (in actuality, the ^5^A_1_ state maps to ^5^A′ while the ^5^E states map to ^5^A′ and ^5^A″ in *C*_s_ symmetry). The contributions of the four excited quintet states to the D⃡ tensor are shown, with values outside and inside of parentheses corresponding to contributions to *D* and *E*, respectively. Positive contributions are shown in blue, while negative contributions are shown in maroon.

It is clear that **1-F** is the exceptional complex in the **1-X** series. The relatively strong σ-donation of fluoride pushes the ^5^A_1_(T_2_) state (state labels from *C*_3v_ symmetry were retained for simplicity) quite high in energy compared to the first excited ^5^E­(E) state. This energy difference reduces the degree of SOC between the ground ^5^E­(E) state and the excited ^5^A_1_(T_2_) state, leading to small rhombicity and a relatively large and negative value for *D*. Conversely, the other **1-X** compounds have weaker σ-donation from their respective halides, which ultimately dramatically lowers the energy of the excited ^5^A_1_(T_2_) state. Thus, the magnitude of *D* becomes similar across the rest of the **1-X** series, and there is much more rhombicity as the SOC between the excited ^5^A_1_(T_2_) and the ground ^5^E­(E) state is greatly strengthened. Compared to the ^5^A_1_(T_2_) state, the energies of the excited ^5^E­(E) and ^5^E­(T_2_) states exhibit only modest changes with halide identity.

Interestingly, the change in the sign of the experimental *D* values from positive in **1-Br** to negative in **1-I** can be explained by the relative energies of the three lowest quintet states. For the **1-Br** complex, the ^5^A_1_(T_2_) state is higher in energy than the excited ^5^E­(E) state. However, in CASSCF/NEVPT2 calculations of the H-only structures of **1-I**, which provide the correct sign of *D* (Table 2), the excited ^5^A_1_(T_2_) state actually is the first excited state, becoming lower in energy than the excited ^5^E­(E) state. In fact, the reason that the sign of *D* computed for the Full Opt (+) geometry of **1-I** is incorrect is due to an incorrect ordering of the excited state energies (Figure 6 vs Figure S18).

We also used ab initio ligand field theory (AILFT) to treat the NEVPT2 transition energies to create an Fe 3*d*-orbital energy level diagram for each **1-X** complex. The canonical 3*d*-orbitals are used to form trigonal symmetry-adapted linear-combination (SALC) orbitals in the *C*_3v_ point group; these are reported in Table S19. The SALC-orbital energies and their corresponding canonical 3*d*-orbital compositions for the H-only and Full Opt geometries (approximately *C*_s_ symmetry) are reported in Tables S20 and S21, respectively, while the orbital energy diagrams are displayed in Figures S19 and S20 for the H-only and Full Opt geometries, respectively. A representative MO diagram for the **1-X** series is presented in Figure 7 for the **1-F** Full Opt (+) isomer.

**Figure 7:**
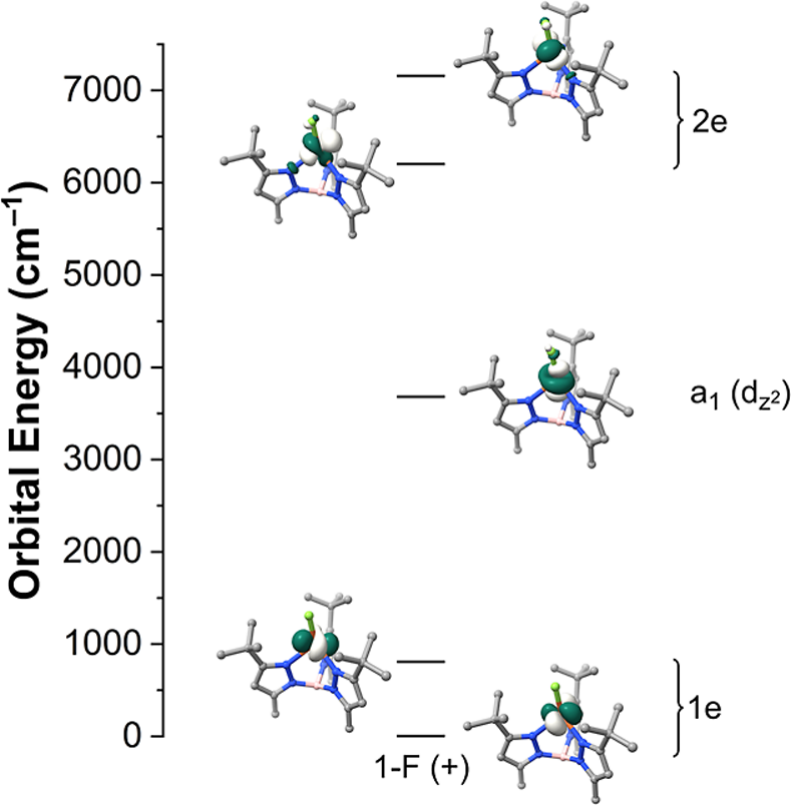
AILFT SALC 3*d*-orbital MO diagram for the ground ^5^E state of the **1-F** Full Opt (+) isomer derived from CASSCF/NEVPT2 calculations (corresponding to the second column in Figure 6). The symmetry labels for the *C*_3v_ point group are used for clarity.

### 2.3 Analysis of Jahn–Teller Effects in the **1-X** Series

The crystallographic, spectroscopic, and computational data presented above consistently point to deviations from *C*_3v_ to approximate *C*_s_ symmetry for each member of **1-X** series. Below, we examine to what extent these structural distortions are the result of the Jahn–Teller effect. In order to rule out solid-state lattice effects, we compared the structural data reported herein to the high-spin Co­(II) congeners reported by us previously,^42^ and measured near-infrared (NIR) absorption spectra in solution. The energies of the observed *d*–*d* transitions were then analyzed with ligand-field theory (LFT) within the framework of the angular overlap model (AOM) using both ab initio and classical approaches. Finally, we present Jahn–Teller potential energy surfaces of the **1-X** complexes derived from the combined experimental and computational results.

#### 2.3.1 NIR Absorption Spectra

The solution state (1.0 mM in CH_2_Cl_2_) NIR absorption spectra for the **1-X** series are displayed in Figure 8. The complexes appear colorless in solution, as their *d*–*d* transitions fall in the near-infrared (NIR) region. Two transitions are apparent, one main peak and one shoulder. Both transitions were deconvoluted by fitting two Gaussian peaks to each spectrum (described in detail in the Supporting Information, Figure S21 and Table S22), and were assigned as *d*–*d* transitions on the basis of their relatively small molar absorptivities and oscillator strengths (Table S22). These band energies were used in the LFT fitting and are reported in Table S23. The splitting between both bands decreases across the **1-X** series, ranging from 2150 cm^–1^ (**1-F**) to 1464 cm^–1^ (**1-I**).

**Figure 8:**
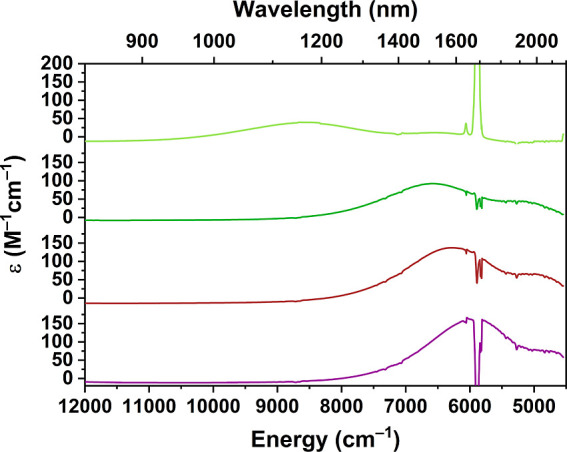
NIR absorption spectra of the **1-X** series (from top to bottom: X = F, Cl, Br, I) measured in CH_2_Cl_2_ (concentration = 1.0 mM). The prominent features at 5817, 5834, and 5889 cm^–1^ are from vibrational overtones from the solvent.^59^

In *T*_d_ point group symmetry, the ground state ^5^D free-ion term of the [Ar]­3*d* ^6^ configuration splits into a ^5^E ground state and a ^5^T_2_ excited state (Scheme 2). In *C*_3v_ point group symmetry (the idealized symmetry for the **1-X** series) the ground state remains ^5^E, but the excited ^5^T_2_ state splits into two states, ^5^A_1_ and ^5^E (Scheme 2). Therefore, one possible assignment (assuming idealized *C*_3v_ symmetry) is that the most intense main bands observed in the spectra for the **1-X** series correspond to the ^5^E­(E) → ^5^E­(T_2_) transition while the shoulders correspond to the ^5^E­(E) → ^5^A_1_(T_2_) transition. A second possibility, however, is that the two observed bands are assigned to splitting of the parent ^5^E­(E) → ^5^E­(T_2_) transition due to distortion of the molecular geometries from *C*_3v_ to *C*_s_ symmetry. If only *C*_s_ symmetry is assumed, then the excited ^5^E­(T_2_) state splits into ^5^A′(E,T_2_) and ^5^A″(E,T_2_) states,^60^ where the terms in parentheses refer to the parent states in *C*_3v_ and *T*_d_ symmetry, respectively (Scheme 2). In this scenario, the lower-energy ^5^A_1_(T_2_) excited-state in *C*_3v_ becomes ^5^A′(A_1_,T_2_) in *C*_s_ symmetry. This second possibility is most consistent with the results from the CASSCF/NEVPT2 calculations presented in the previous section, which point to a large energetic splitting between the three states derived from the parent ^5^T_2_ term (in *T*_d_ symmetry).

**Scheme 2:**
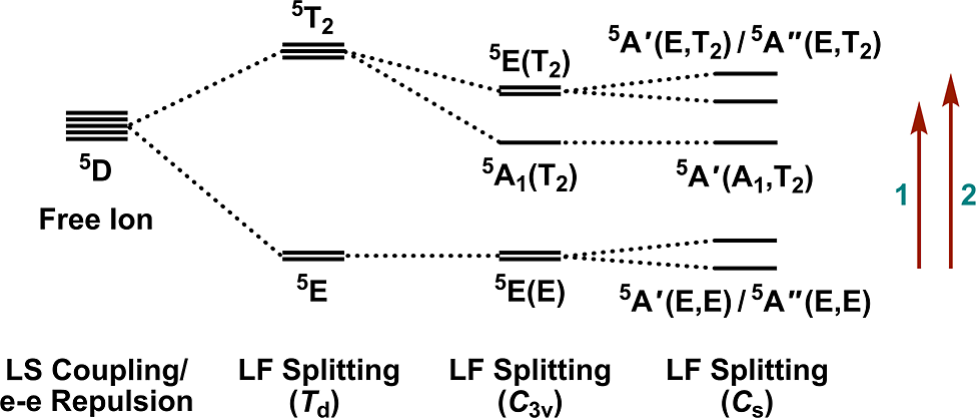
Due to Russell-Saunders Coupling and Electron–Electron Repulsion the [Ar]­3*d* ^6^ Configuration Splits into the ^5^D Free Ion Ground State and Numerous Free Ion Triplet and Singlet Excited States (Not Shown)^a^

a Ligand-field splitting in *T*_d_, *C*_3v_, and *C*_s_ symmetry further splits the ^5^D term into various states as shown. Spin–orbit coupling effects on the energies were ignored. Both NIR transitions are indicated with red arrows.

Computed NIR absorption spectra for the **1-X** series are provided in Figures S22 and S23 for H-only and Full Opt structures, respectively. The computed peak energies exhibit reasonable agreement with the experimental values. Significantly, the calculations reproduce the red-shifts in band energies and the corresponding reduction in peak splittings (for the Full Opt geometries) going from F to I. Based on the CASSCF/NEVPT2 results, the two bands in the region of 5000–10,000 cm^–1^ arise from transitions to the ^5^A′(E,T_2_) and ^5^A″(E,T_2_) excited states, as illustrated in Scheme 2. In contrast, the transition to the lower-energy ^5^A′(A_1_,T_2_) state lies outside of the detection window of the NIR absorption spectra at energies less than 4000 cm^–1^.

#### 2.3.2 Ligand Field Analysis

The NIR data were further analyzed using the angular overlap model (AOM)^61,62^ to understand ligand-field trends across the **1-X** series. We first utilized AILFT matrices to obtain AOM parameters. The results of the AOM parametrizations for each of the **1-X** complexes based on the NEVPT2 transition energies are reported in Tables S24 and 3 for the H-only and Full Opt geometries, respectively. Consistent with the 3*d*-orbital energies shown in Figure 7, the AOM parameters provide further evidence that fluoride is a much stronger σ-donor compared to the other halide ligands. Interestingly, the negative ε_π_(X) parameters for **1-Br** and **1-I** suggest that the heavier halides actually act as weak π-acceptors. This suggests that low-energy empty 4*d*- (Br) and 5*d*-orbitals (I) are capable of accepting π-electron density from the Fe­(II) centers. Racah *B* and *C* parameters were calculated, along with nephelauxetic ratios (β_*B*_ and β_*C*_), to parametrize the ligand field and assess covalency in the **1-X** series. The compounds are highly ionic, with *B* and *C* parameters only slightly reduced compared to their free-ion values (*B*_free‑ion_ = 1020 cm^–1^ and *C*_free‑ion_ = 3965 cm^–1^)^63^ and nephelauxetic ratios close to the ionic limit of β = 1,^64^ which remain static across the **1-X** series.

**Table 3:** AOM Parametrization of the Fe–Ligand Interactions in the **1-X** Series for the Full Opt Geometries (NEVPT2 Energies) and the Corresponding Racah Parameters^a^

	**1-F**		**1-Cl**	**1-Br**	**1-I**
	full (−)	full (+)	full (+)	full (+)	full (+)
ε_σ_(X)	5090	4983	2474	1945	1584
ε_π_(X)	1929	1912	180.6	–242.5	–626.3
ε_σ_(N)	4160	4104	4084	4121	4157
ε_πs_(N)	603.1	530.4	638.8	727.3	867.9
σ_SD_	130.7	140.7	115.1	93.47	82.83
*B*	929.9	930.3	918.8	919.9	919.9
*C*	3630	3631	3621	3628	3631
*C*/*B*	3.903	3.903	3.941	3.944	3.947
β_*B*_ ^b^	0.912	0.912	0.901	0.902	0.902
β_*C*_ ^b^	0.916	0.916	0.913	0.915	0.916

a All values are in cm^–1^, except *C*/*B* and β ratios, which are dimensionless.

b β_*B*_ = *B*_complex_/*B*_free‑ion_ and β_*C*_ = *C*_complex_/*C*_free‑ion_ are the nephelauxetic ratios. The free-ion Racah parameters for Fe­(II) are *B*_free‑ion_ = 1020 cm^–1^ and *C*_free‑ion_ = 3965 cm^–1^.^63^

We also attempted to fit the NIR absorption data with classical LFT, as described in the Supporting Information (Table S23). However, this approach proved incapable of reproducing the experimental transition energies, although parameters resembling the AILFT values could be obtained if only the higher-energy band was fitted (Tables S25 and S26). Comparing the AOM results from both classical and ab initio LFT (Table S26) suggests that the splittings between the observed NIR bands are much larger than what would be expected from minor deviations from *C*_3v_ symmetry due to lattice-strain effects. This discrepancy is a manifestation of the Jahn–Teller distortion energy, which cannot be reproduced via classical LFT arguments, consistent with the vibronic coupling analysis given in the following section.

#### 2.3.3 Vibronic Coupling and Jahn–Teller Analysis

Tetrahedral (*T*_d_) high-spin Fe­(II) complexes with ^5^E ground states are predicted by the Jahn–Teller theorem to be geometrically unstable. Molecular vibrations of the same E-symmetry (denoted hereafter by ε) interfere with the ^5^E electronic ground state leading to distorted geometries with point group symmetries belonging to subgroups of *T*_d_ symmetry (*D*_2d_ and lower). Such vibrations lead to symmetry breaking, lifting the orbital degeneracy. Lower-symmetry Fe­(II) *C*_3v_ complexes, such as those featuring trigonal scorpionate ligands, exhibit a ^5^E ground state arising from the 1*e* ^3^ *a*_1_^1^2*e* ^2^ configuration. In contrast, the high-spin Co­(II) congeners studied by us previously, [Co^II^X­(Tp^*t*Bu,Me^)] (X = F, Cl, Br, I), have a ^4^A_2_ ground state (1*e* ^4^ *a*_1_^1^2*e* ^2^ configuration) for which no Jahn–Teller distortions are expected to occur.^42^

Structural data on the herein reported **1-X** series of complexes show a clear descent from idealized *C*_3v_ to lower symmetries, evident by Fe–N_Tp_ bond distance and X–Fe–N_Tp_ bond angle deviations (Tables S3–S5 and summarized in Table S12). The structural distortion pattern (Table S12) is that of an overall shortening (lengthening) of one (two) Fe–N_Tp_ bonds accompanied by an increase (decrease) of the corresponding X–Fe–N_Tp_ angles.

A complete quantitative analysis of the energetic and structural aspects of the ^5^E ⊗ ε Jahn–Teller effect in the **1-X** series is intractable due to the large number of vibrations with ε-symmetry. Because each ε-symmetry vibration can potentially contribute to Jahn–Teller distortions, the result is an unsolvable multimode vibronic coupling problem. To this end, we applied the effective interaction mode concept put forward by Bersuker,^65^ which considers a single local mode to be a superposition of all molecular and lattice vibrations. There are two local modes of ε-symmetry. The first is a stretching mode, *q*_str_, which effects deviations of the Fe–N_Tp_ bond lengths Fe–N1, Fe–N3, and Fe–N5 (d*R*_FeN_1__, d*R*_FeN_3__, d*R*_FeN_5__, respectively) from their average value, d*R*_FeN_avg__. The second is a bending mode, *q*_bend_, which distorts the X–Fe–N_Tp_ bond angles *θ*_XFeN_1__, *θ*_XFeN_3__, and *θ*_XFeN_5__ (d*θ*_XFeN_1__, d*θ*_XFeN_3__, and d*θ*_XFeN_5__, respectively) from their average value, *θ*_XFeN_avg__. Scheme 3 depicts the atom labeling and movements of both vibrations. Each mode, *q*_str_ and *q*_bend_, consists of two submodes; one of these, *Q*_θ_, tends to preserve the highest possible (epikernel) symmetry that breaks the electronic degeneracy (in the present case *C*_s_, A′(*C*_s_)). The other, *Q*_ε_, is orthogonal to the first and distorts the trigonal *C*_3v_ geometry to lower symmetry (kernel, here *C*_1_, A″(*C*_s_)). Remarkably, structural data of the **1-X** series exhibit distortions along submodes of A′ symmetry (Table S27). This leads to structures with approximate *C*_s_ symmetry, in accordance with the epikernel principle, which states that Jahn–Teller distortions tend toward higher-symmetry (epikernel) structures over lower-symmetry (kernel) ones.^66,67^

**Scheme 3:**
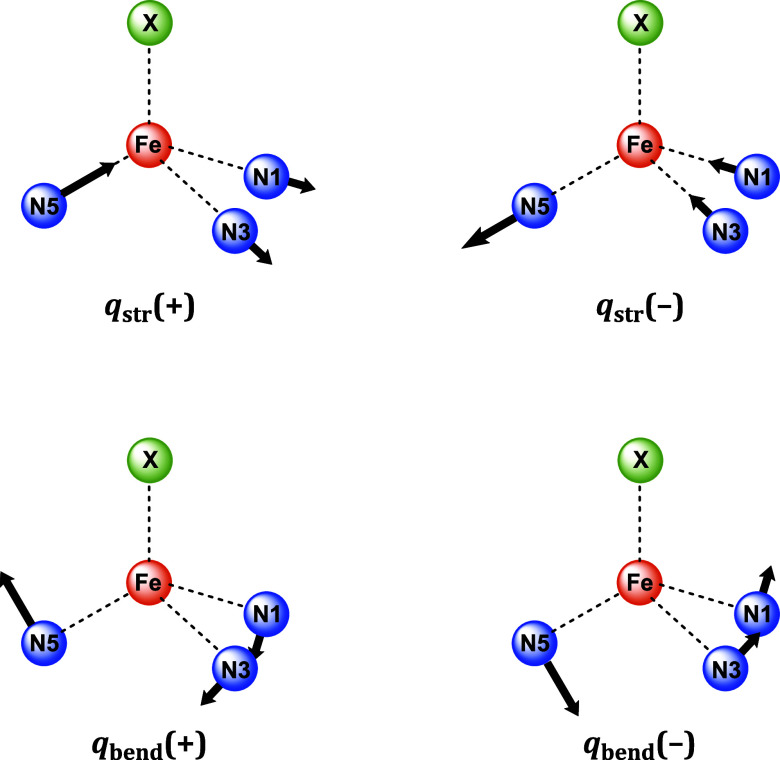
Shapes of the Jahn–Teller Modes *q*_str_ (Top) and *q*_bend_ (Bottom), with Both the Forward (+) and Backward (−) Distortions Depicted

When constructing a ^5^E ⊗ ε Jahn–Teller potential energy surface for a system with trigonal symmetry, one would expect three energetically equivalent minima if quadratic effects are included. Alternatively, if quadratic effects are ignored, the result is isoenergetic minima along a circular trough.^67,68^ Regardless, one of the minima points would lie along the *Q*_θ_ axis, such that *Q*_ε_ = 0. By choosing this specific point, expressions for *q*_str_ and *q*_bend_ may be defined according to eqs 1–4:

1 $$q_{str}=\frac{1}{\sqrt{6}}\left(dR_{{FeN}_{1}}+dR_{{FeN}_{3}}-2\;dR_{{FeN}_{5}}\right)$$

2 $$q_{bend}=\frac{1}{\sqrt{6}}R_{{FeN}_{avg}}\left(d\theta_{{XFeN}_{1}}+d\theta_{{XFeN}_{3}}-2\;d\theta_{{XFeN}_{5}}\right)$$

3 $$dR_{{FeN}_{i}}=R_{{FeN}_{i}}-R_{{FeN}_{avg}}$$

4 $$d\theta_{{XFeN}_{i}}=\theta_{{XFeN}_{i}}-\theta_{{XFeN}_{avg}}$$

where *R*_FeN_avg__ is the average Fe–N_Tp_ bond distance (in Å) and *θ*_XFeN_avg__ is the average X–Fe–N_Tp_ bond angle (in radians). This simplified approach allows for the extraction of values for *q*_str_ and *q*_bend_ from the X-ray data (i.e., the H-only geometries) or from the Full Opt geometries (summarized in Table S12) by defining *q*_str_ ≡ *Q*_θ_^str^ and *q*_bend_ ≡ *Q*_θ_^bend^. The resulting values are listed in Table 4.

**Table 4:** Vibronic Parameters were Extracted from the Data Summarized in Table S12 and the Calculated Δ*E*_FC_^min^ Energies Using the Simple Model Described in the Text; Energies are in Units of cm^–1^, *F*_bend_ is in Units of cm^–1^ Å^–1^, and *K*_bend_ is in Units of cm^–1^ Å^–2^ ^a^

	**1-F**			**1-Cl**		**1-Br**		**1-I**	
	H-only	full (−)	full (+)	H-only INUTUH01 Mol. A(B)	full (+)	H-only Mol. A(B)	full (+)	H-only Mol. A(B)	full (+)
*q*_str_	0.026	0.032	–0.040	0.029(0.023)	–0.034	0.038(0.036)	–0.030	0.040(0.031)	–0.027
*q*_bend_	–0.353	–0.409	0.369	–0.152(−0.302)	0.182	–0.326(−0.242)	0.129	–0.268(−0.263)	0.098
Δ*E*_FC_^min^	836.6	1023	978.9	402.3(692.0)	422.7	845.8(696.5)	309.5	875.1(710.2)	261.0
*F*_bend_	–1186	–1250	1325	–1320(−1147)	1159	–1296(−1439)	1199	–1635(−1349)	1333
*K*_bend_	3363	3056	3589	8660(3805)	6352	3970(5946)	9285	6113(5121)	13616
Δ*E*_JT_	209.2	255.7	244.7	100.6(173.0)	105.7	211.5(174.1)	77.38	218.8(177.6)	65.26

a H-only geometries derived from previously reported X-ray data for **1-Cl** (INUTUH01)^17^ were used instead of the H-only geometry reported herein due to the crystallographically-imposed trigonal symmetry precluding Jahn–Teller analysis.

The ^5^E ⊗ ε Jahn–Teller problem in the basis of the ^5^E ground state and linear vibronic coupling (i.e., ignoring quadratic vibronic coupling) has the matrix representation of eq 5.^69^ It describes the splitting of the ^5^E ground state into sublevels, |^5^E­(A′)⟩ and |^5^E­(A″)⟩, reflected by the diagonal elements, *FQ*_θ_ and −*FQ*_θ_ (where *F* is the linear vibronic coupling constant in units of cm^–1^ Å^–1^), as well as their mixing via off-diagonal elements, −*FQ*_ε_. The term $\frac{1}{2}K\left({Q_{\theta }}^{2}+{Q_{\varepsilon }}^{2}\right)$ is the restoring force energy (where *K* is the harmonic force constant in units of cm^–1^ Å^–2^).

HE5⊗ε=|E5(A′)⟩|E5(A″)⟩[12K(Qθ2+Qε2)+FQθ−FQε−FQε12K(Qθ2+Qε2)−FQθ] 5

The values reported in Tables S27 and 4 show that *q*_bend_ is much more prominent across the **1-X** series; therefore *q*_str_ may be ignored because the Jahn–Teller stabilization energies for the *q*_bend_ modes dominate. Furthermore, comparison with the analogous [Co^II^X­(Tp^*t*Bu,Me^)] series reveals that *q*_bend_ modes are much stronger in the **1-X** series (Table S27), suggesting that Jahn–Teller distortions are the dominant contributor to the structural distortions rather than lattice strain effects. From the diagonal elements of eq 5 (minimizing with respect to *Q*_θ_^bend^), expressions in terms of *F*_bend_ and *K*_bend_ for ±*q*_bend_ at the energetic minimum, as well as Jahn–Teller splitting of the ground ^5^E state at the minimum, Δ*E*_FC_^min^, were obtained using eqs 6 and 7

6 $$\pm q_{bend}=\frac{\pm F_{bend}}{K_{bend}}$$

7 $$\Delta E_{FC}^{\min}=2\left|F_{bend}\cdot q_{bend}\right|$$

The value of ±*q*_bend_ is defined as ±*Q*_θ_^min^ at the energetic minimum (from either the H-only or Full Opt geometries). The Δ*E*_FC_^min^ parameter is the Franck–Condon energy (i.e., the vertical excitation energy) corresponding to the energetic splitting of the ground ^5^E state. This value is calculated by CASSCF/NEVPT2 (neglecting spin–orbit coupling) using NEVPT2 diagonal energies of the output file (Table 4). More details of this approach were described previously by Atanasov and Neese et al.^16^

The ground state potential surface (PES) of a ^5^E ⊗ ε Jahn–Teller complex in this (linear) treatment is a sombrero potential. An example for **1-F** is shown in Figure 9a,b, in which *Q*_θ_ and *Q*_ε_ refer to the high-symmetry (*C*_s_, A′(*C*_s_)) and low-symmetry (*C*_1_, A″(*C*_s_)) submodes of *q*_bend_, respectively. A cross section along the *Q*_ε_ = 0 plane is shown in Figure 9c. The cross-sectional plot in Figure 9c shows the possible coexistence of *C*_s_ symmetry distortions in different directions along *Q*_θ_, which we refer to as the forward (+) and backward (−) distorted geometries (Scheme 3). According to Figure 9c, these geometries represent different minima having the same energy. Remarkably, gas-phase DFT optimizations of **1-F** were able to locate the (+) and (−) distorted geometries, which have almost identical energies.

**Figure 9:**
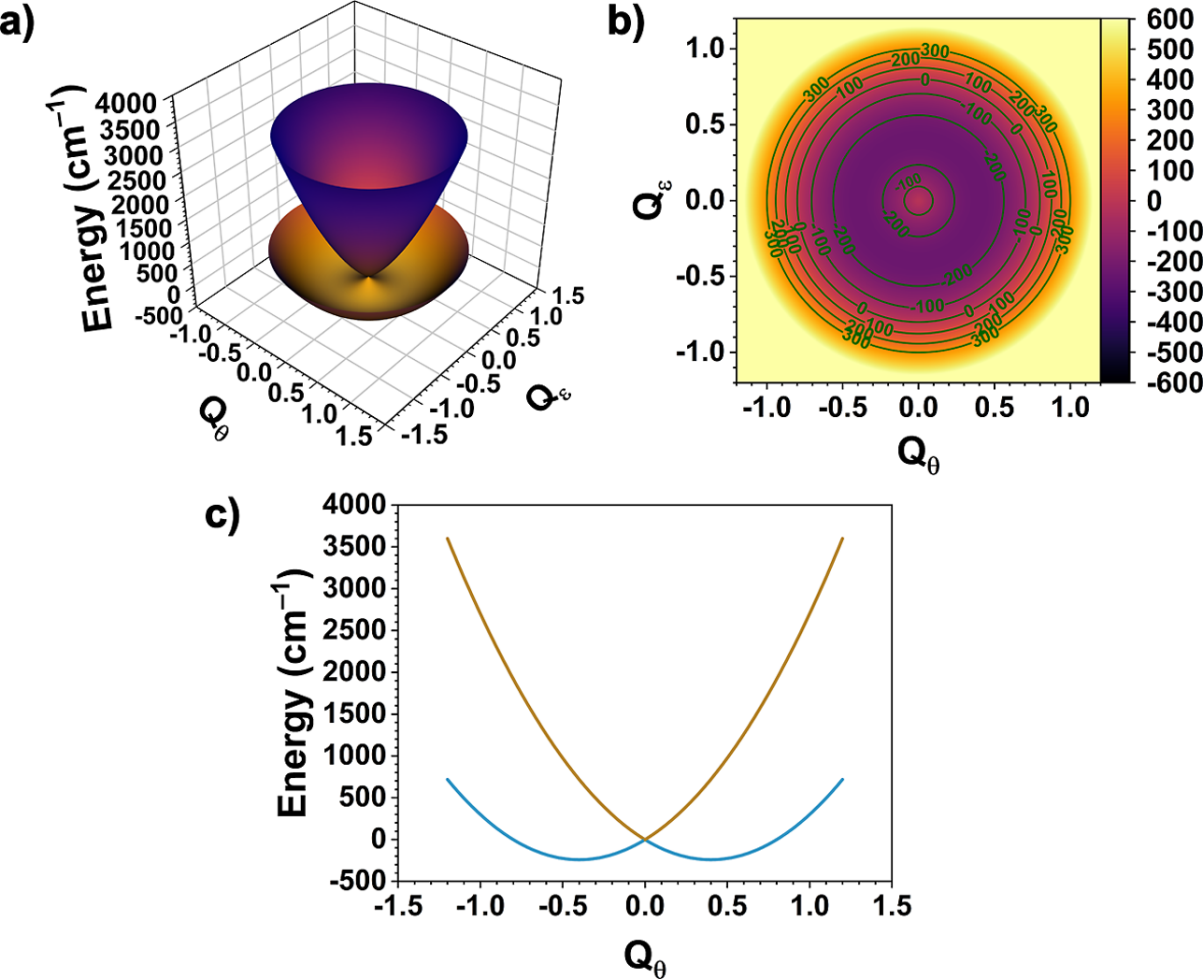
(a) Jahn–Teller ground state sombrero potential energy surface (PES) for the *q*_bend_ mode of **1-F** defined by the ^5^E ⊗ ε Jahn–Teller problem; (b) a top-down view of the PES; (c) the cross section along the *Q*_ε_ = 0 plane in the linear coupling limit; the plots were made using eq 5 with *F*_bend_ = 1200 cm^–1^ Å^–1^ and *K*_bend_ = 3000 cm^–1^ Å^–2^, approximated using structural and energy data from the CASSCF/NEVPT2 calculations on **1-F** (see Tables S12 and 4, respectively); units of energy are in cm^–1^.

To assess the possibility of quadratic vibronic coupling effects, we utilized the (−) and (+) geometries for **1-F** to estimate that the barrier, δ, between energetic minima and saddle points on the sombrero PES is δ = 11.00 cm^–1^; such a small barrier, as well as a very small quadratic vibronic coupling constant of only |*G*_bend_| = 37.34 cm^–1^ Å^–2^, suggest free pseudorotation (for a gas-phase molecule) around the trough of the PES (see eqs S1 and S2 in the Supporting Information for more details). Therefore, we ignored quadratic vibronic coupling across the entire **1-X** series because these effects are negligible compared to the linear vibronic coupling, and a full analysis of both would require an approach far beyond the scope of this paper.^68^

The Jahn–Teller stabilization energy, Δ*E*_JT_ (eq 8), is defined as the difference between the energies of the minima referenced to the energy of the trigonal *C*_3v_ geometry

8 $$\Delta E_{JT}=\frac{1}{2}\frac{{F_{bend}}^{2}}{K_{bend}}=\frac{1}{4}\Delta E_{FC}^{\min}$$

The magnitude of Δ*E*_JT_ quantifies the Jahn–Teller activity across the **1-X** series. The Δ*E*_JT_ values provided in Table 4 show that the vibronic coupling strength diminishes from **1-F** to **1-Cl** to **1-Br** to **1-I**. The underlying mechanism is the energetic lowering of ligand-to-metal charge transfer (LMCT) excited states, as the halide ligands become more reducing (less electronegative) from F to I. This trend slightly increases the contribution of the Jahn–Teller inactive 1*e* ^4^ *a*_1_^1^2*e* ^2^ (i.e., a Fe^I^–X^•^ LMCT state) configuration into the ground-state wave function. The energetic lowering of LMCT states from **1-F** to **1-Cl** to **1-Br** to **1-I** leads to an increase in their mixing into the ^5^E electronic ground-state wave function of the **1-X** complexes, thereby reducing the Jahn–Teller stabilization energies with heavier halides.^70^

Remarkably, the high-spin Co­(II) congeners exhibit similar structural distortion patterns as the **1-X** series reported herein, albeit they are much smaller.^42^ In the [Co^II^F­(Tp^*t*Bu,Me^)] complex, a similar yet much weaker (−) distorted geometry is seen (see Table S27). We hypothesize that this distortion arises from lattice strain in crystals of [Co^II^F­(Tp^*t*Bu,Me^)], as the gas-phase fully optimized structure exhibits almost ideal *C*_3v_ symmetry. Conversely, the presence of two well-resolved quadrupole doublets in the zero-field Mössbauer spectrum at 1.8 K of **1-F** indicates that, under the experimental conditions, both the forward (+) and backward (−) distortions along the *q*_bend_ mode coexist (Figure 9c). Table 4 shows that the Jahn–Teller stabilization energy of **1-Cl** is half the size of **1-F**. This may be the reason why the X-ray structure for **1-Cl** shows a trigonal *C*_3v_ complex without distortion. However, previously reported X-ray structural data exhibit orthorhombic symmetry, allowing us to analyze the Jahn–Teller effect for **1-Cl** as well; two different sites appear in the unit cell, a consequence of the space group in which the compound crystallized, with each site exhibiting a similar (−) distortion.^17^ An attempt to obtain a DFT-optimized geometry for the (−) *q*_bend_ Jahn–Teller isomer for **1-Cl** failed. Conversely, one previously reported^71^ X-ray structure of [Co^II^Cl­(Tp^*t*Bu,Me^)] shows the expected (−) *q*_bend_ geometry although the distortion is very small with near-trigonal symmetry (the two more recent reports of [Co^II^Cl­(Tp^*t*Bu,Me^)] show structures with trigonal symmetry^42,72^ ). The zero-field Mössbauer spectrum at 1.8 K for **1-Cl** exhibits two broadened quadrupole doublets, with one corresponding to the predominant *q*_bend_ distortion and the other to the minority, which is consistent with these theoretical observations.

Computed Jahn–Teller stabilization energies are smaller for **1-Br** and **1-I**. While two different sites appear in their unit cells, this is again merely a consequence of the space group in which they crystallized, considering that both molecules in the unit cells exhibit similar *q*_bend_ (−) distortions. The lowering of the computed Jahn–Teller stabilization energies for **1-Br** and **1-I** is consistent with the single well-resolved quadrupole doublets in each of their corresponding zero-field Mössbauer spectra at 1.8 K.

## 3 Discussion

Herein, we examined the role of the halide identity on the zero-field splitting (ZFS) of a series of scorpionate high-spin Fe­(II)-halide complexes (**1-X**; X = F, Cl, Br, or I). The compounds were characterized by X-ray crystallography, which showed that the halide ligands distort away from the idealized *C*_3v_ symmetry to approximate *C*_s_ symmetry by moving off the *C*_3_ axis of rotation, with the exception of **1-Cl**, which exhibits crystallographic trigonal symmetry. We investigated each compound with magnetometry, X-band EPR (for **1-F**), high-frequency and -field electron paramagnetic resonance (HFEPR) (for **1-Cl** and **1-Br**), far-infrared magnetic spectroscopy (FIRMS), and variable-temperature/-field (VTVH) ^57^Fe Mössbauer spectroscopy to understand their electronic structures by modeling the data with the spin-Hamiltonian, which provided a consistent set of parameters between the experimental techniques (provided in Table 2). The relationships between halide identity, molecular geometries, ligand-field energies, and ZFS were elucidated using CASSCF/NEVPT2 calculations, as well as ab initio ligand field theory (AILFT).

We also measured NIR-absorption spectra on the compounds, which revealed that the structural distortions observed in the solid state (via crystallography) are retained in solution. Analysis of the vibronic coupling indicated that the geometric distortions of the compounds away from the idealized *C*_3v_ symmetry are the result of Jahn–Teller distortions that lift the degeneracy of the ^5^E ground states. This work represents one of the few studies on structural distortions due to the Jahn–Teller effect in molecular tetrahedral high-spin Fe­(II) complexes.

In our previous work, we systematically investigated the corresponding [Co^II^X­(Tp^*t*Bu,Me^)] (X = F, Cl, Br, I) complexes.^42^ In that series, the ZFS for the fluoride complex was found to be the smallest with |*D*| = 3.9 cm^–1^ and with a rhombicity near the rhombic limit (*E*/*D* = 0.326), while the chloride, bromide, and iodide complexes had ZFS values of *D* = 11–13 cm^–1^ and *E*/*D* = 0.012–0.172 (becoming more and more rhombic from Cl to Br to I). We concluded that the Co–X bonds were highly ionic, and thus that their ZFS was due to ligand-field effects rather than from intrinsic spin–orbit coupling (SOC) from the halide ligands, as proposed previously.^43,73–78^ The unique ZFS parameters for the [Co^II^F­(Tp^*t*Bu,Me^)] complex result instead from the so-called “fluoro effect”, whereby the strong σ-donation from the fluoride ligand destabilized the low-lying ^4^E state relative to the ^4^A_2_ ground state, leading to a small ZFS.

The ZFS parameters for the **1-X** series herein can be explained partly by a similar argument. Given the relatively invariant isomer shifts from the zero-field ^57^Fe Mössbauer spectra, we can conclude that the Fe–X bonds in the **1-X** series are also ionic. This lack of covalency is further supported by the calculated Racah parameters, which are close to the free-ion values for Fe­(II).^63^ The calculated nephelauxetic ratios, β_*B*_ and β_*C*_, are ∼0.90–0.92, close to the ionic limit of β = 1, suggesting that the electron clouds of the Fe­(II) centers do not expand to participate in covalent bonds with the halides in the **1-X** series.^64^ Furthermore, the nephelauxetic effect is absent across the **1-X** series, where it would be expected that the nephelauxetic ratios would decrease from **1-F** to **1-I** due to increased Fe–X covalency, following the nephelauxetic series.^79^ This lack of covalency means that the Fe 3*d*-orbitals lack halide character (evident from Figure 7), and hence there is no pathway to increase the ZFS via intrinsic SOC contributions from the halide ligands. Were this the case, it would be expected that the **1-I** complex would have the largest ZFS and the **1-F** complex the smallest according to the expected intrinsic SOC of the halogens (the one-electron effective SOC constants are 404.1, 882.4, 3685, and 7603 cm^–1^ for neutral F, Cl, Br, and I, respectively, which each have n*p* ^5^ configurations^80^). In fact, the opposite is true, with the **1-F** complex exhibiting the largest ZFS. We turned to AILFT to assess the changes, if any, in the one-electron effective SOC constant, ζ. The results, reported in Table S18, show that ζ remains essentially constant (∼390–400 cm^–1^) across the entire **1-X** series, essentially unchanged from the free-ion value of ζ = 400 cm^–1^ for Fe­(II).^81^ Thus, the idea that intrinsic SOC from the halides contributes to the ZFS has no support from experiment or theory for the **1-X** series of complexes.

Instead, the ZFS parameters of the **1-X** series are the result of a combination of Jahn–Teller distortion and ligand-field effects. In these systems, the **1-F** complex has the largest ZFS with *D* = −20.8 cm^–1^ (determined by magnetometry) and is the most axial with *E*/*D* ≈ 0.07–0.11 (determined by X-band EPR and VTVH ^57^Fe Mössbauer). The strong σ-donation from the fluoride ligand greatly destabilizes the second excited ^5^A_1_ excited state, while the Jahn–Teller distortion to *C*_s_ symmetry lifts the degeneracy of the ground ^5^E state. The high electronegativity of elemental fluorine (4.193 on the Allen scale^82^) decreases the ability for ligand-to-metal charge transfer (LMCT) from the halide to the high-spin Fe­(II) center, which causes the **1-F** complex to have a relatively large Jahn–Teller stabilization energy, leading to a large energetic splitting in the ^5^E ground state. In contrast, the **1-Cl**, **1-Br**, and **1-I** complexes have smaller ZFS parameters of *D* = +12.12, +10.741, and −12.29 cm^–1^, respectively (determined by modeling the HFEPR/FIRMS data for **1-Cl** and **1-Br** and the magnetometry data for **1-I**), with rhombicities of *E*/*D* ≈ 0.24–0.27. The ability of the LMCT states to mix into the ground state ^5^E wave function increases across the series from **1-Cl** to **1-Br** to **1-I**, a result of the smaller electronegativities of their corresponding halogens (2.869, 2.685, and 2.359, respectively^82^). However, it should be noted that this mixing of excited LMCT states into the ground state is very small, such that the Fe–X bonds remain very ionic across the series, with any slight changes in covalency having only a minor impact on the isomer shifts observed by Mössbauer spectroscopy.^83–85^ Nonetheless, this increase in mixing of the LMCT states across the **1-X** series causes the Jahn–Teller stabilization energies to decrease across the series, and the ground ^5^E state splitting diminishes. At the same time, the ^5^A_1_ second excited state decreases in energy across the series due to the weaker and weaker σ-donation from the halides. The combined ligand-field and Jahn–Teller effects on the energies of the first ^5^E and second ^5^A_1_ excited states is summarized in Figures 6 and S18. This also explains the change in the sign of *D* from positive to negative going from **1-Br** to **1-I**, where the ^5^A_1_ excited state becomes lower in energy than the ^5^E excited state for the H-only optimized geometries (Figures 6 and S18). Based on these results, we once more propose that the “fluoro effect” is operative in the **1-X** series, just with the inverse effects given the differences between the ground states in the **1-X** high-spin Fe­(II) series and their high-spin Co­(II) counterparts.

Comparison of the crystal structures of the **1-X** series reported herein to those of our previous work on the analogous high-spin Co­(II) series of complexes shows that the structural distortions in the Fe­(II) compounds studied herein are much larger (see Table S27). We therefore conclude that the distortions to approximate *C*_s_ symmetry away from idealized *C*_3v_ symmetry primarily result from Jahn–Teller distortion to lift the degeneracy of the ^5^E ground state rather than lattice strain effects in the solid state, an unusual example of the Jahn–Teller effect given that these systems are four-coordinate tetrahedral complexes with *d* ^6^ configurations.^86^ This is a manifestation of the relatively strong ligand field imparted by the pyrazolyl N atom donors (Tables S24 and 3), whereas compounds with weaker ligand fields, such as [Fe^II^(SR)_4_]^2–^ complexes, do not exhibit Jahn–Teller distortions.^15^

## 4 Conclusions

We once more conclude that, in essence, the observed zero-field splittings (ZFS) in the high-spin Fe­(II) **1-X** series are not the result of a so-called “heavy-atom” effect of the halides, where an increase in intrinsic halide spin–orbit coupling (SOC) has been proposed previously to induce increases in the ZFS. In fact, the observed ZFS in the **1-X** series are exactly the opposite of what one would predict from a “heavy-atom effect”, in that **1-F** has the largest magnitude ZFS. Instead, the ZFS results from ligand-field effects, in agreement with our previous study on the analogous high-spin Co­(II) complexes,^42^ but here with the added complication of the **1-X** series exhibiting Jahn–Teller distortions. We conclude that halide identity *does* impact the ZFS, but in such highly ionic systems through mechanisms other than simply from intrinsic SOC contributed by the halide ligands. The magnitude, sign, and rhombicity of the ZFS are all difficult to predict a priori, but future work on analogous complexes with other metals and ancillary ligands will hopefully shed light on systematic trends.

## Supplementary Material



## Data Availability

All ASCII data sets, along with the AOM MATLAB scripts and LFT fitting software is included in the Edmond Open Research Data Repository at the following link: 10.17617/3.XNQCUE.

## Notes

Magnetometry data for **1-Cl** and **1-Br**, error plots of magnetometry fits, X-band EPR spectrum of **1**-**F**, HFEPR spectra of **1**-**Cl**, FIRMS spectra, powder X-ray diffraction data for **1**-**F**, fit 1 and fit 2 of the zero-field ^57^Fe Mössbauer spectrum for **1**-**F**, zero-field ^57^Fe Mössbauer spectra for **1**-**Br** and **1**-**I**, VTVH ^57^Fe Mössbauer data for **1**-**Cl**, **1**-**Br**, and **1**-**I**, alternate fit of VTVH ^57^Fe Mössbauer spectra for **1**-**F**, Gaussian deconvoluted NIR-absorption spectra, computed NIR-absorption spectra, experimental and computational methods, single-crystal X-ray diffraction parameters, magnetometry fit table, HFEPR/FIRMS data and fit tables, ^57^Fe Mössbauer fit tables, summary of bond distances and angles for DFT-optimized geometries, computed D⃡ -tensor contribution tables, comparison of spin-Hamiltonian parameters obtained from including only SOC vs SOC + SSC table, calculated effective one-electron spin–orbit coupling constants (ζ) table, AILFT 3*d*-orbital SALC tables, Gaussian deconvolution of NIR-absorption spectra table, AOM/LFT/AILFT tables, vibronic coupling and Jahn–Teller data table, and DFT geometry *xyz*-coordinate tables (PDF)

## ○

Waste Management, Inc., 17629 Cedar Springs Ln, Arlington, Oregon 97812, United States

## ◆

Los Alamos National Laboratory, Bikini Atoll Rd, Los Alamos, New Mexico 87545, United States.

## ††

PPD, Inc., 3230 Deming Way, Middleton, Wisconsin 53562, United States.
