# Supplementary material for: Magnetospectroscopic Studies of a Series of Fe(II) Scorpionate Complexes: Assessing the Relationship between Halide Identity and Zero-Field Splitting
Source: Inorg Chem. 2025 Jul 29;64(31):16135–51. doi: 10.1021/acs.inorgchem.5c02691 (PMC12344776; doi:10.1021/acs.inorgchem.5c02691)
Supplement: Supplementary file 1 [file ic5c02691_si_001.pdf]

Electronic Supporting Information for

Magnetospectroscopic Studies of a Series of Fe(II) Scorpionate Complexes: Assessing the Relationship between Halide Identity and Zero-Field Splitting

Daniel J. SantaLucia,[*,†] Laxmi Devkota,[‡,‡‡] Sergey V. Lindeman,[‡] Andrew Ozarowski,[§] J. Krzystek,[§] Mykhaylo Ozerov,[§] Samuel M. Greer,[§,||,§§] Daniel C. Cummins,[⊥,||||] Klaus H. Theopold,[⊥] **Mihail Atanasov,**[*,¶,◊] **Joshua Telser,**[*,††] and **Adam T. Fiedler**[*,‡]

[†] Max Planck Institute for Chemical Energy Conversion, Stiftstraße 34-36, Mülheim an der Ruhr, North Rhine-Westphalia D-45470, Germany

[‡] Department of Chemistry, Marquette University, 1414 W Clybourn St, Milwaukee, Wisconsin 53233, United States

[§] National High Magnetic Field Laboratory, Florida State University, 1800 E Paul Dirac Dr, Tallahassee, Florida 32310, United States

[||] Department of Chemistry and Biochemistry, Florida State University, 95 Chieftain Way, Tallahassee, Florida 32306, United States

[⊥] Department of Chemistry and Biochemistry, University of Delaware, Brown Laboratory, Newark, Delaware 19716, United States

[¶] Max-Planck-Institut für Kohlenforschung, Kaiser-Wilhelm-Platz 1, Mülheim an der Ruhr, North Rhine-Westphalia D-45470, Germany

[◊] Institute of General and Inorganic Chemistry, Bulgarian Academy of Sciences, Acad. Georgi Bonchev Street 11, BG-1113, Sofia, Bulgaria

[††] Department of Biological, Chemical and Physical Sciences, Roosevelt University, 430 S Michigan Ave, Chicago, Illinois 60605, United States

[‡‡] Current Address: Waste Management, Inc., 17629 Cedar Springs Ln, Arlington, Oregon 97812, United States

[§§] Current Address: Los Alamos National Laboratory, Bikini Atoll Rd, Los Alamos, New Mexico 87545, United States

[||||] Current Address: PPD, Inc., 3230 Deming Way, Middleton, Wisconsin 53562, United States

Email contact: daniel.santalucia@cec.mpg.de (D.J.S.), mihail.atanasov@kofo.mpg.de (M.A.), jtelser@roosevelt.edu (J.T.), adam.fiedler@marquette.edu (A.T.F.)
Fax number: (+1) 414-288-7066 (A.T.F.); (+1) 312-341-4358 (J. T.)

Contents

Supplementary Magnetometry Figures: S3
Supplementary HFEPR/FIRMS Figures: S6
Supplementary Powder X-ray Diffraction Pattern Figure: S10
Supplementary Mössbauer Figures: S11
Supplementary Electronic Structure Calculations Figures: S18
Supplementary NIR Absorption Spectra Figures: S20
Experimental and Computational Methods: S23
X-ray Crystallographic Data Tables: S38
Magnetometry Fit Table: S42
HFEPR/FIRMS Data and Fit Tables: S43
Mössbauer Fit Tables: S45
Electronic Structure Calculations Tables: S47
AOM/LFT/AILFT Tables: S62
Vibronic Coupling and Jahn Teller Analysis Table: S65
Tables of *xyz* Coordinates for DFT-Optimized Geometries: S66
References: S92

Supplementary Magnetometry Figures:

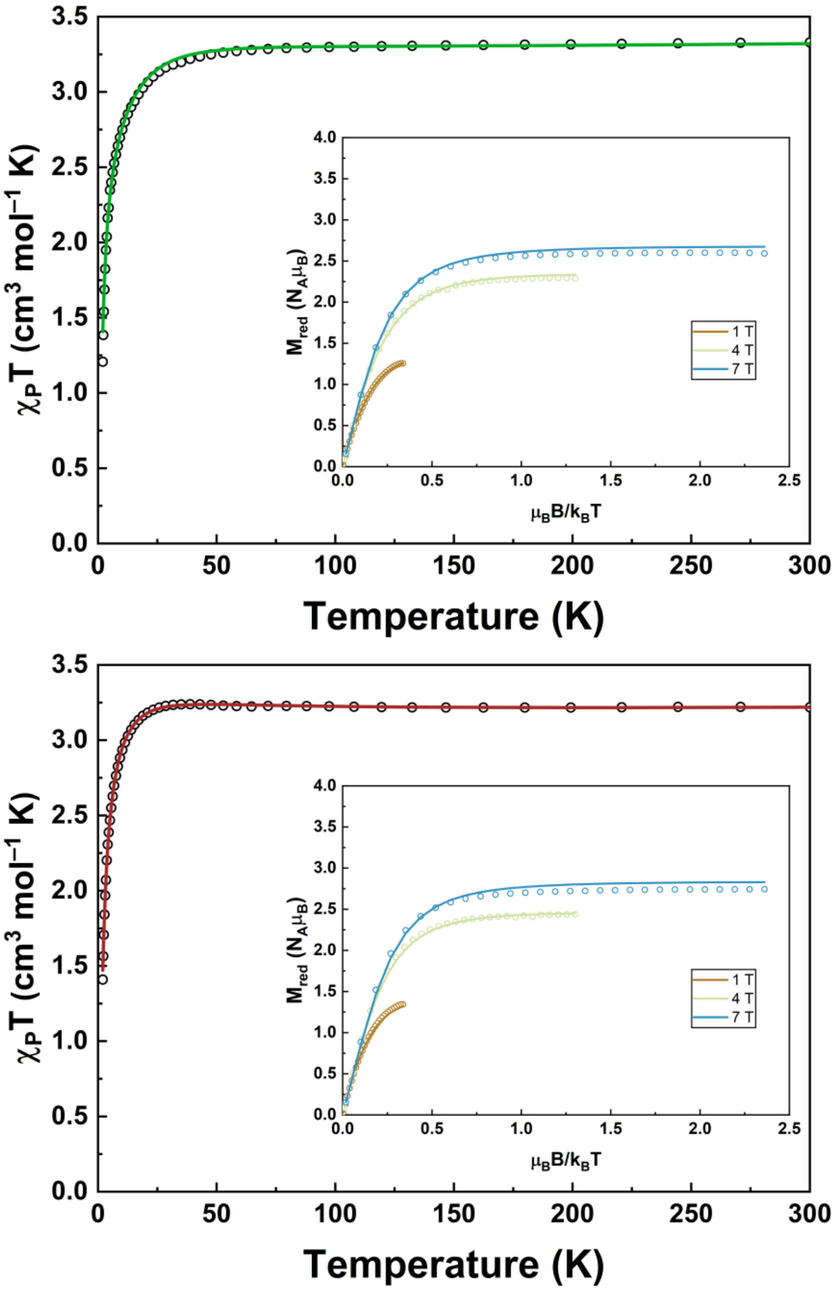

Figure S1. Paramagnetic susceptibility of **1-Cl** (top) and **1-Br** (bottom) plotted as $\chi_P T$ vs. T from 2 to 300 K. Insets: VTVH reduced magnetization measurements at 1, 4, and 7 T isofields. The solid lines are the fits generated from the spin-Hamiltonian, where simultaneous fitting to both the susceptibility and VTVH data was performed. The fit parameters are reported in Table S6.

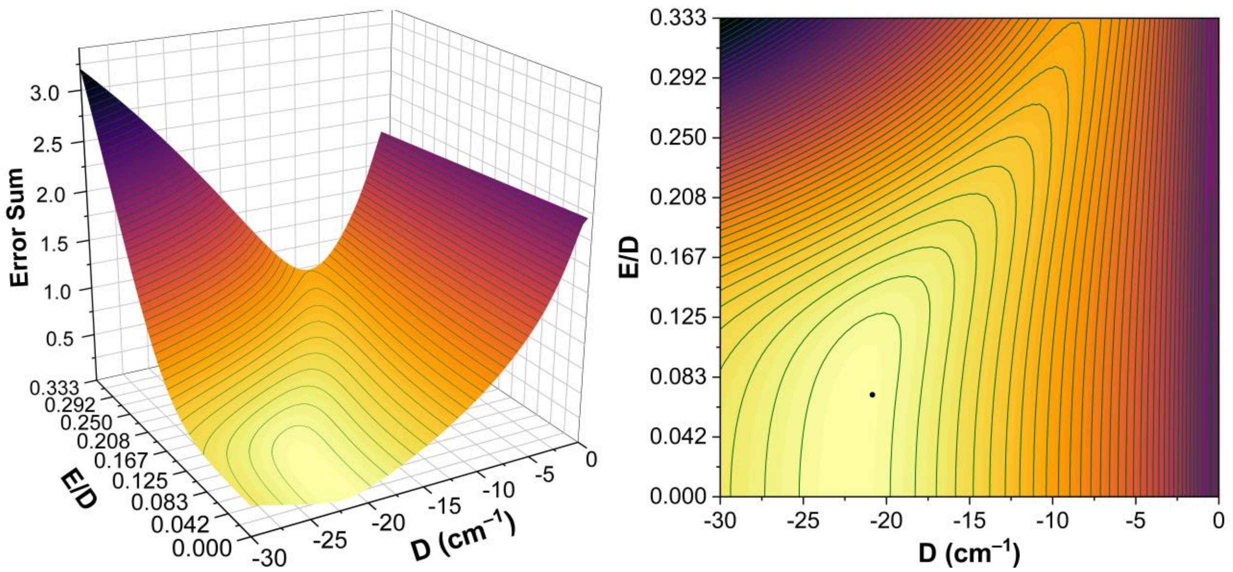

Figure S2. 3D error plot (left) and 2D contour plot (right) for the spin Hamiltonian fit of the paramagnetic susceptibility and VTVH reduced magnetization data for **1-F** over a range of possible D and E/D values. The point in the contour plot is where the fit converged.

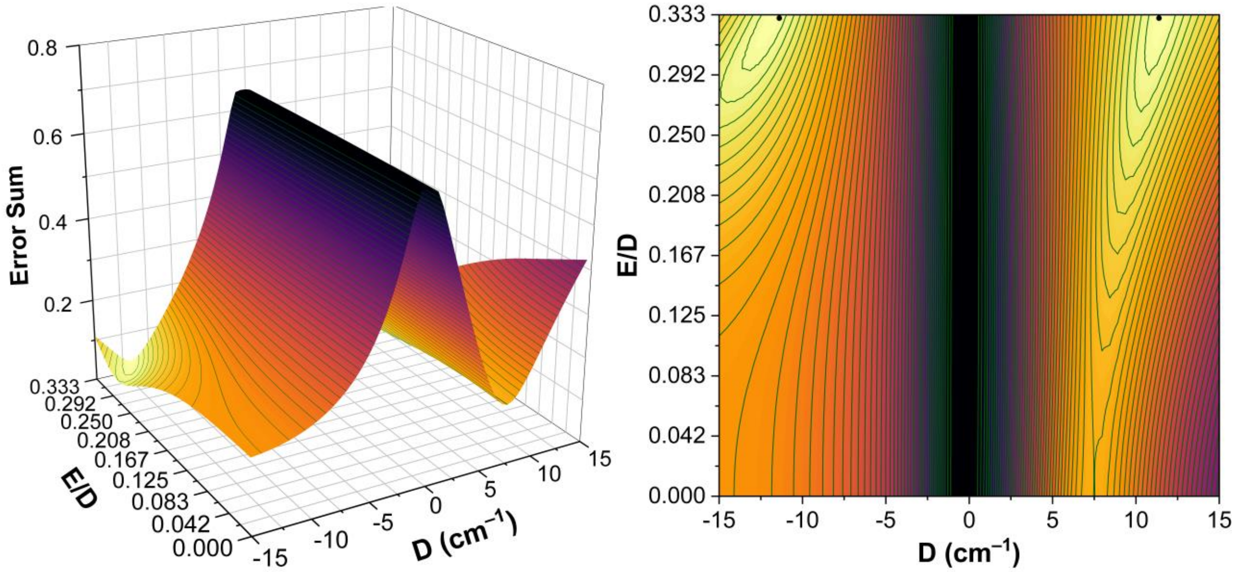

Figure S3. 3D error plot (left) and 2D contour plot (right) for the spin Hamiltonian fit of the paramagnetic susceptibility and VTVH reduced magnetization data for **1-Cl** over a range of possible D and E/D values. The points in the contour plot is where the fit converged (at the rhombic limit, the sign of D is irrelevant so both points are shown).

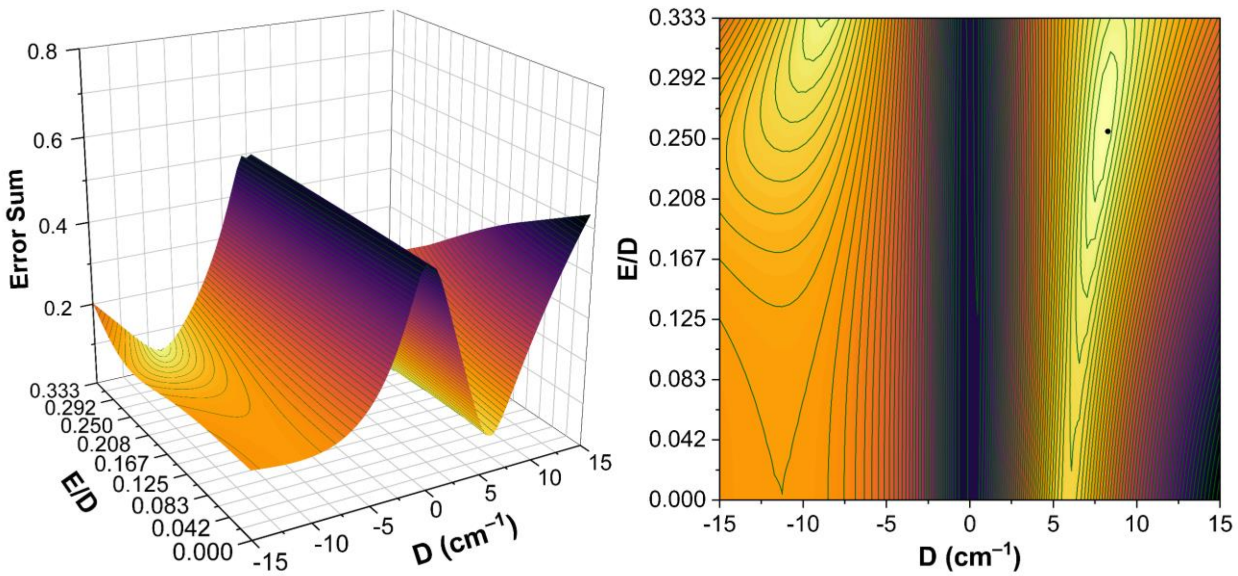

Figure S4. 3D error plot (left) and 2D contour plot (right) for the spin Hamiltonian fit of the paramagnetic susceptibility and VTVH reduced magnetization data for **1-Br** over a range of possible D and E/D values. The point in the contour plot is where the fit converged.

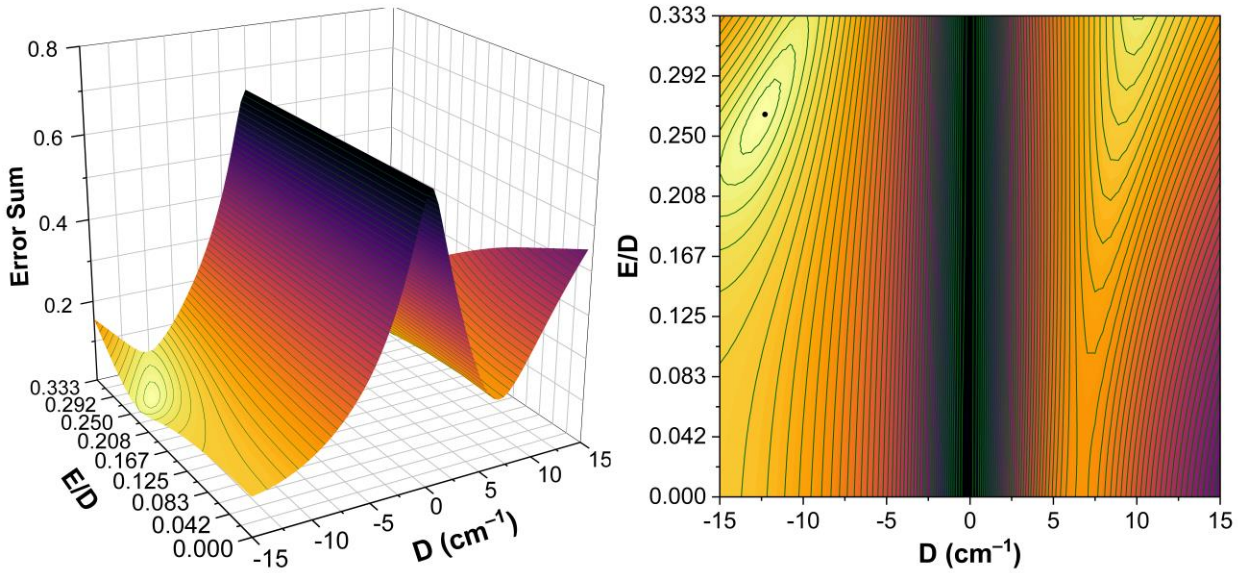

Figure S5. 3D error plot (left) and 2D contour plot (right) for the spin Hamiltonian fit of the paramagnetic susceptibility and VTVH reduced magnetization data for **1-I** over a range of possible D and E/D values. The point in the contour plot is where the fit converged.

Supplementary HFEPR/FIRMS Figures:

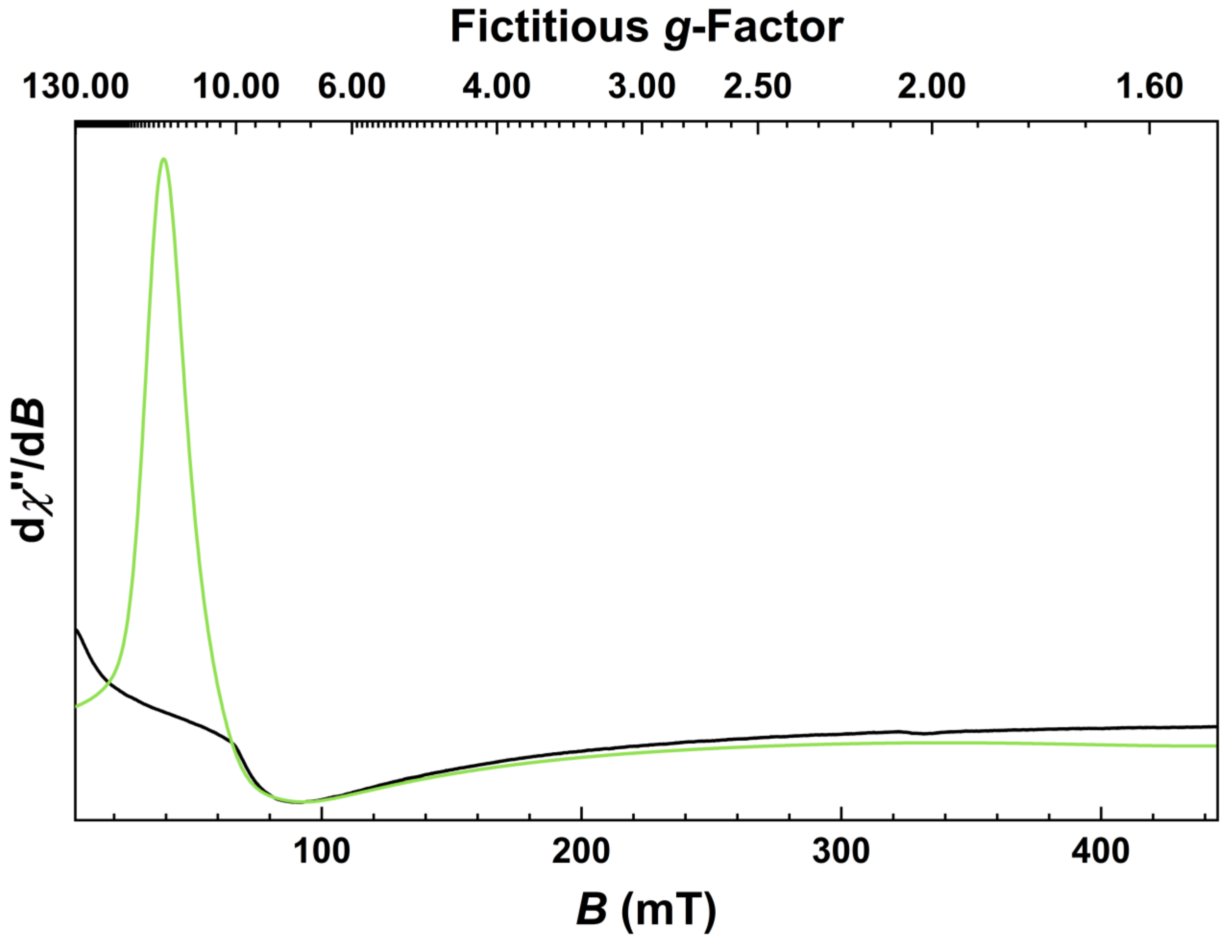

Figure S6. X-band EPR spectrum of a solution of **1-F** in 1:1 toluene:dichloromethane collected at 4 K at a frequency of 9.375 GHz (perpendicular mode). The experimental data are in black while the spin-Hamiltonian simulation is in yellow-green. Parameters for the spin Hamiltonian simulation were mostly fixed from the values obtained from simulating the magnetometry data (Table S6), while the E/D ratio was varied to obtain a good fit. The parameters for the simulation were: $S = 2$, $g_x = g_y =$ 2.028 (fixed), $g_z =$ 2.394 (fixed), $D =$ –20.8 cm^{-1} (fixed), and $E/D =$ 0.0655 (fit). The line shape was a pseudo-Voigt line shape (linear combination of Lorentzian and Gaussian) with a frequency-dependent linewidth of 25 • 10^{-4} cm^{-1} GHz^{-1}.

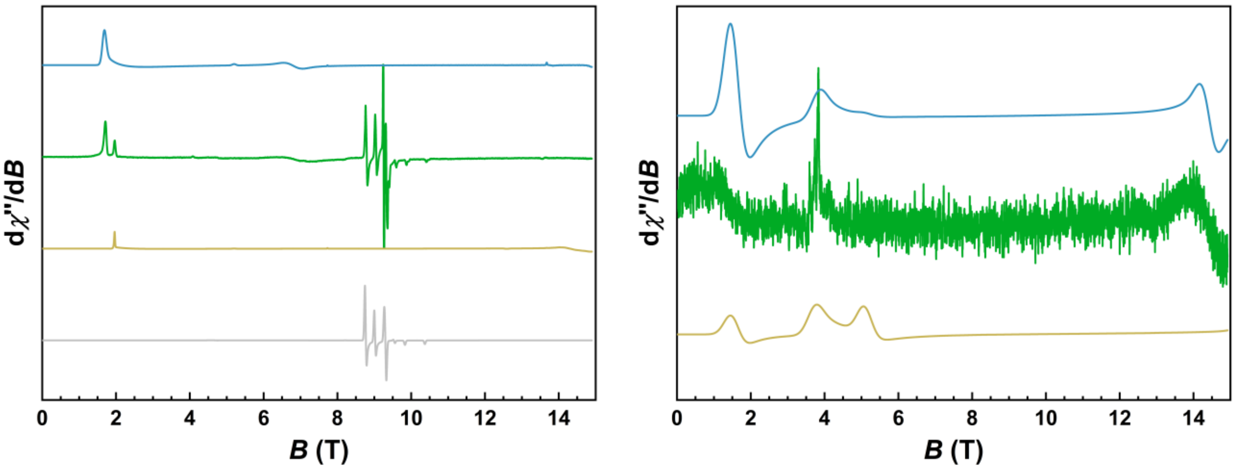

Figure S7. HFEPR spectra for **1-Cl** collected at 10 K with frequencies of 260 GHz (left) and 481 GHz (right). The experimental data are in green while spin Hamiltonian simulations using positive and negative ZFS parameters are in blue and gold, respectively. The spin Hamiltonian parameters for the simulation are found in Table S9. The features from ~8.7-10.4 T in the 260 GHz spectrum correspond to an Fe(III) impurity, and were simulated (gray trace) with the following parameters: $g_{\mathrm{avg}} = g_{\mathrm{e}} = 2.002$, $D = +0.252$ cm^{-1}, and $E/D = 0.000$.

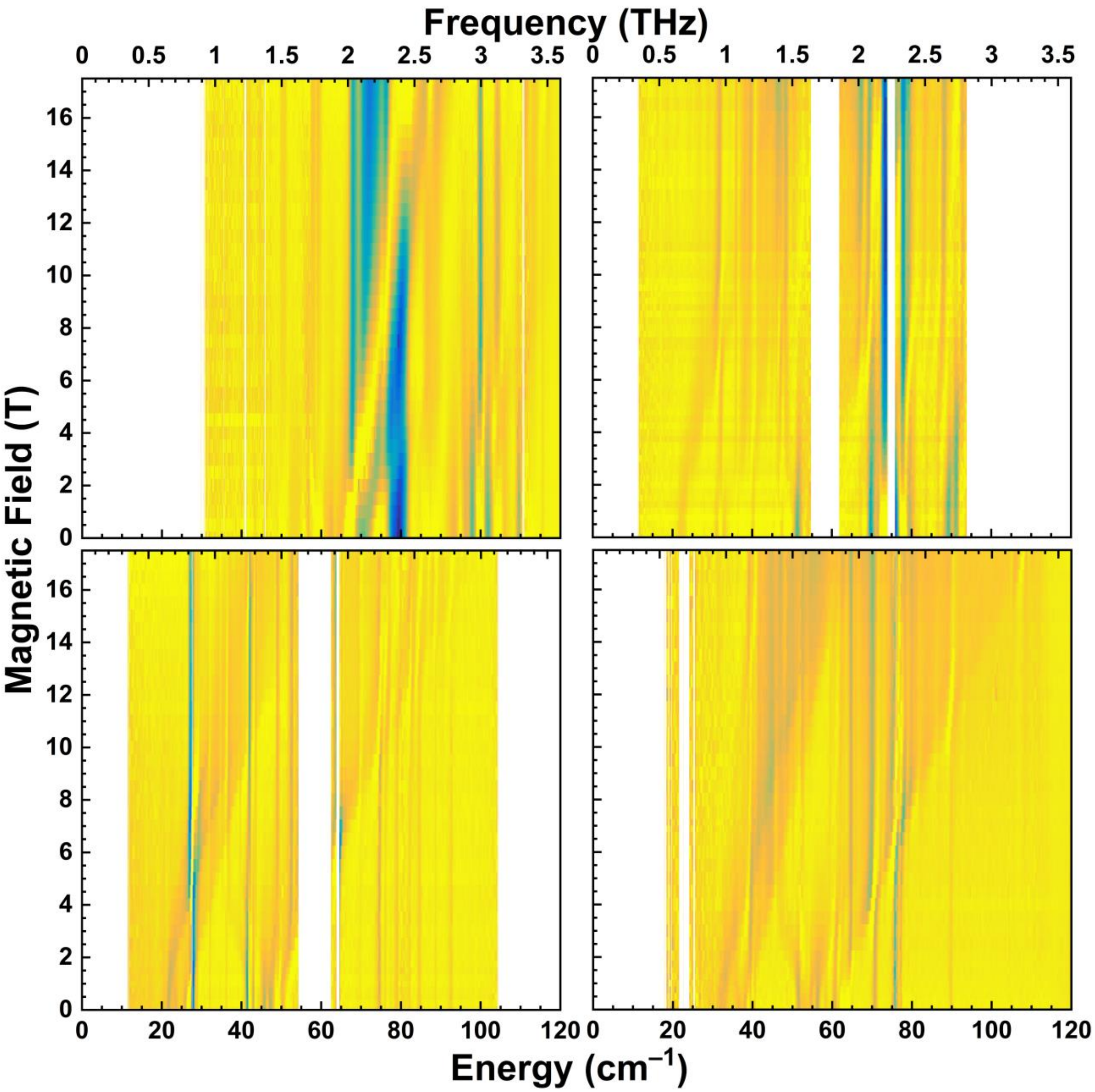

Figure S8. FIRMS data for the **1-X** series from 0-120 cm^{-1} as a function of applied magnetic field up to 17.5 T. Yellow and blue represent maximum and minimum transmittance, respectively. Top left: **1-F**; top right: **1-Cl**; bottom left: **1-Br**; and bottom right: **1-I**. Near vertical lines represent phonons and nonmagnetic vibrational modes while the energies of the magnetic dipole-allowed transitions exhibit field dependence. The zero-field transitions are reported in Table S7.

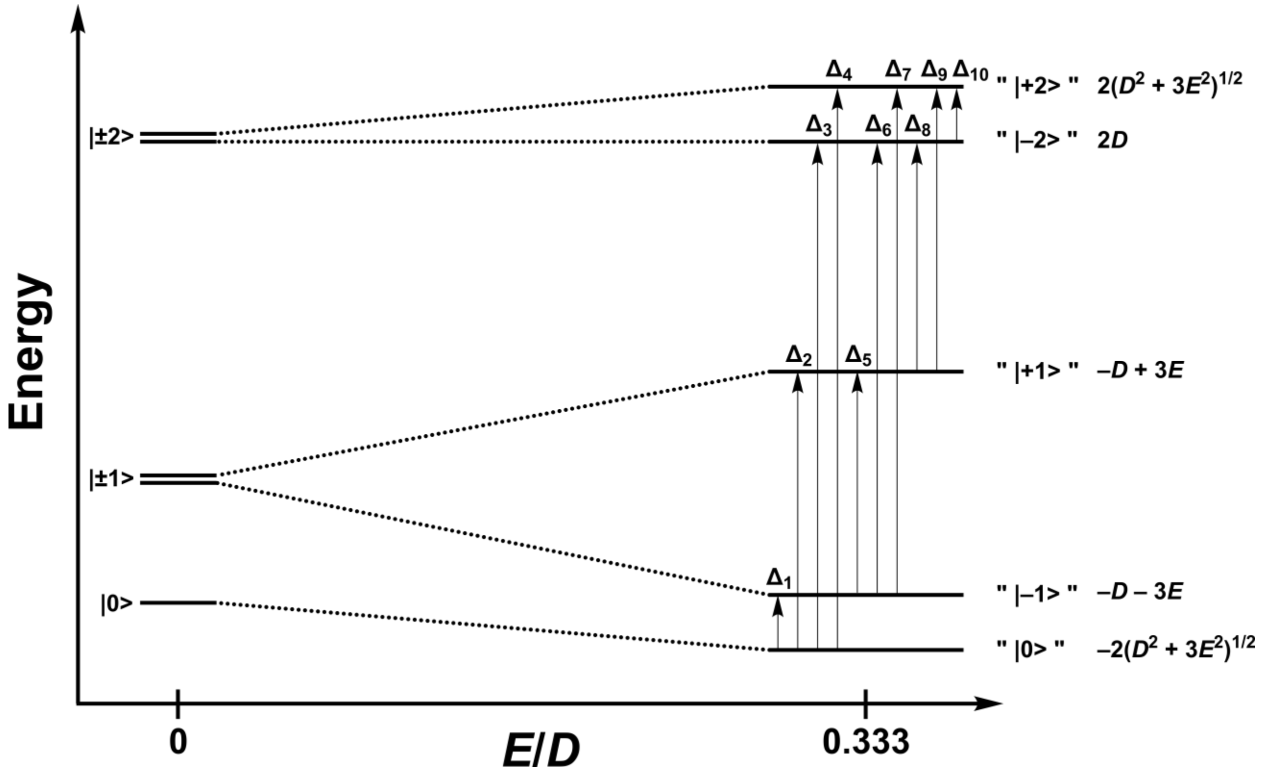

Figure S9. Relative energies of the M_S levels for an $S = 2$ system at the axial and rhombic limits for a positive D parameter (the fourth-order ZFS terms B_4^0 and B_4^3 are ignored here for simplicity). At the rhombic limit, the M_S levels are in quotations since they have mixed with one another and are no longer pure eigenfunctions. For a negative D parameter, the energetic ordering is reversed.

Supplementary Powder X-ray Diffraction Pattern Figure:

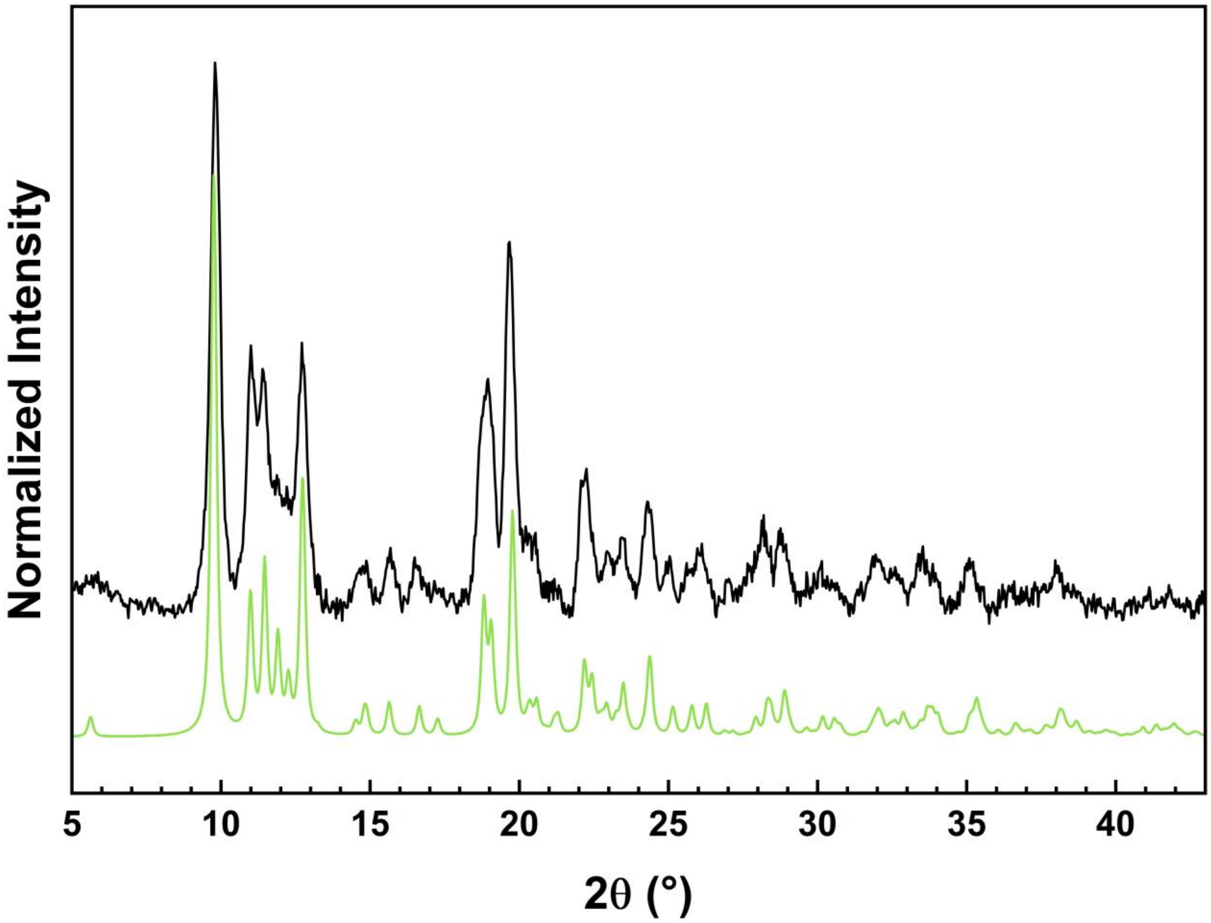

Figure S10. Normalized and baseline-corrected powder X-ray diffraction (PXRD) pattern of **1-F** collected at 298 K averaged over three scans (black trace) along with the simulated PXRD pattern from the single-crystal XRD data (yellow-green trace) collected at 100 K. The simulated data were shifted by 2θ = –0.20° to account for the differences in unit cell sizes due to the difference in temperature between both experiments leading to offset peak positions.

Supplementary Mössbauer Figures:

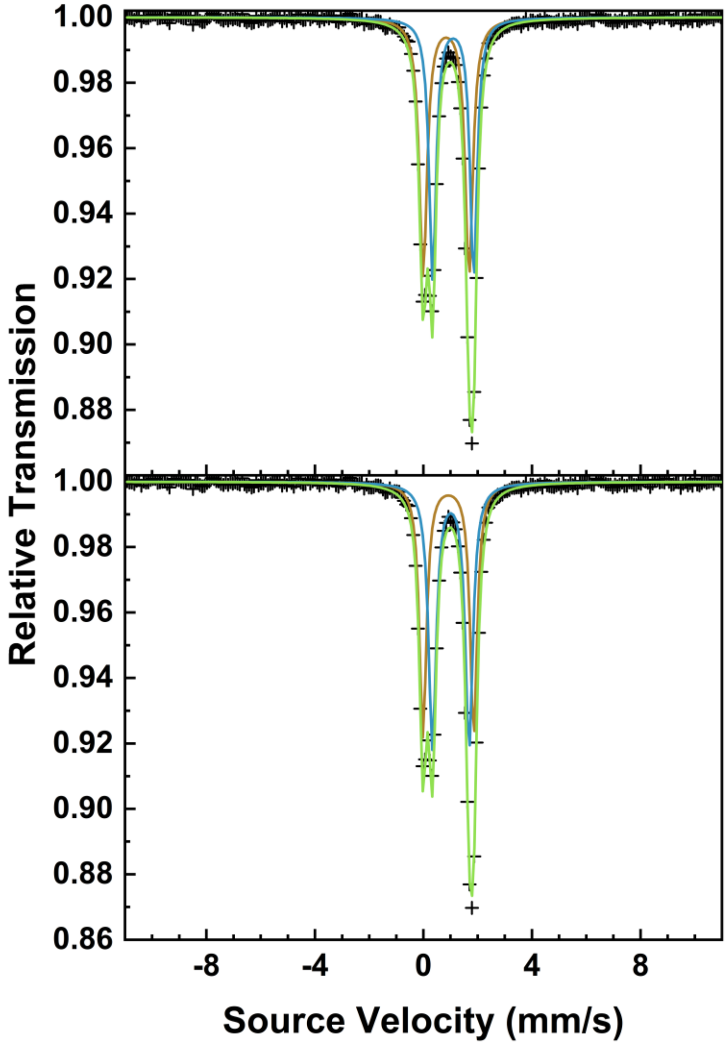

Figure S11. Zero-field Mössbauer spectrum of **1-F** with fit 1 (top) and fit 2 (bottom). The solid lines correspond to the spin-Hamiltonian model, where brown is the first subspectrum, blue the second subspectrum, and yellow-green the overall fit to the data. Fit parameters are reported in Table 1 of the main text.

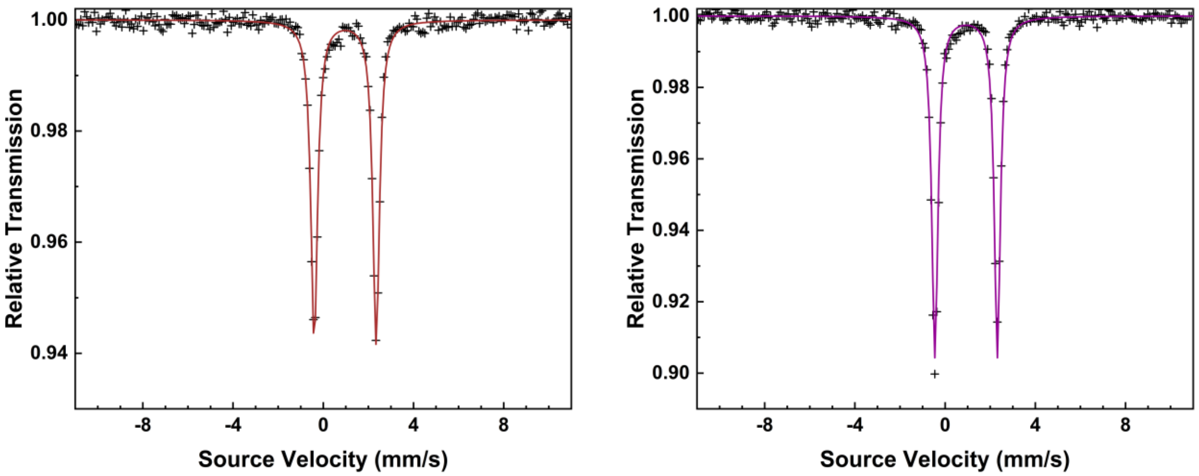

Figure S12. Zero-field (0.05 T) Mössbauer spectrum of **1-Br** (left), and **1-I** (right) with fits to the data. The solid maroon and purple lines correspond to the spin-Hamiltonian models to the data for **1-Br** and **1-I**, respectively. Fit parameters for all spectra are provided in Table 1 of the main text.

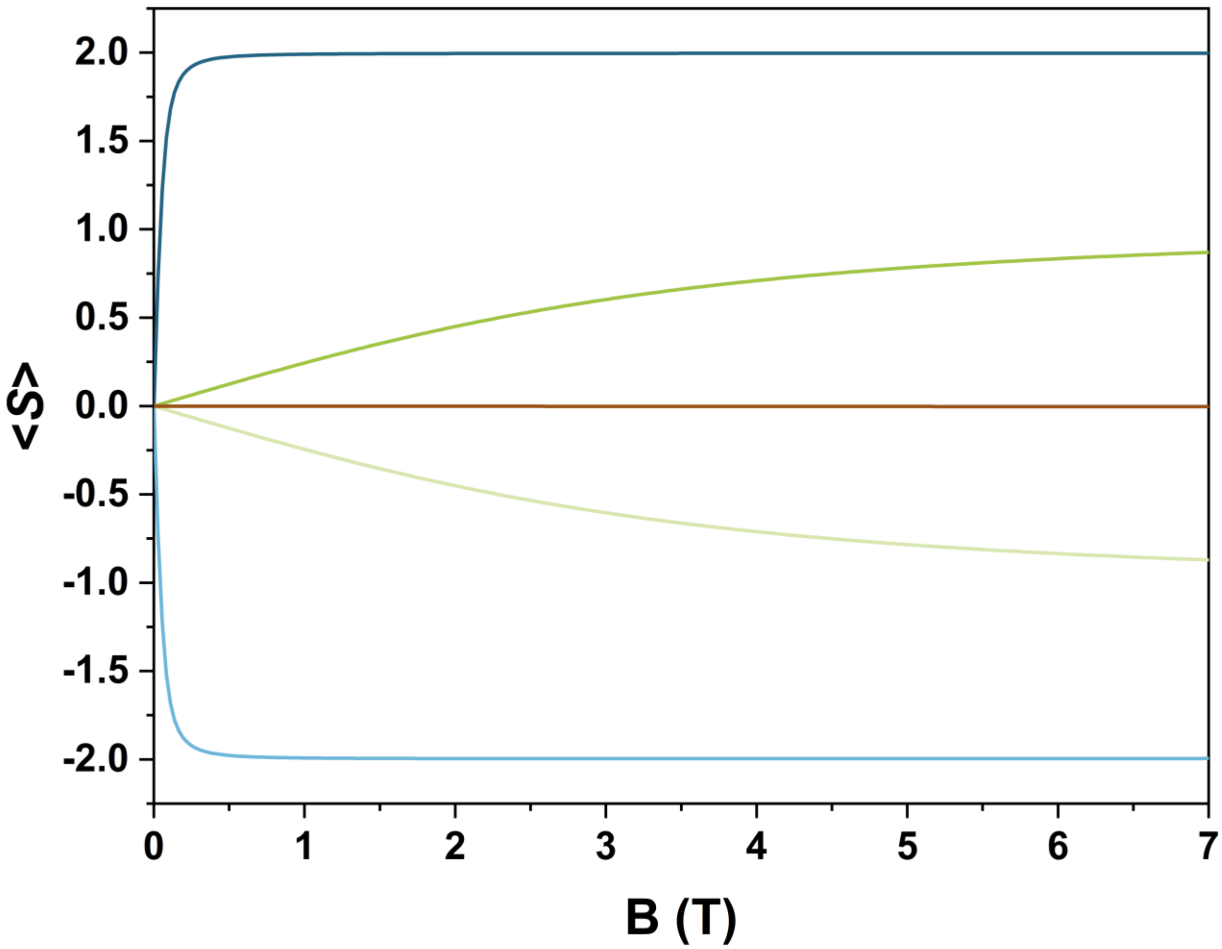

Figure S13. Spin expectation values $\langle S \rangle$ for each M_S level as a function of magnetic field for **1-F** at with varying magnetic field; $M_S = 0$ (brown), $M_S = +1$ and –1 (dark and light green, respectively), and $M_S = +2$ and –2 (dark and light blue, respectively).

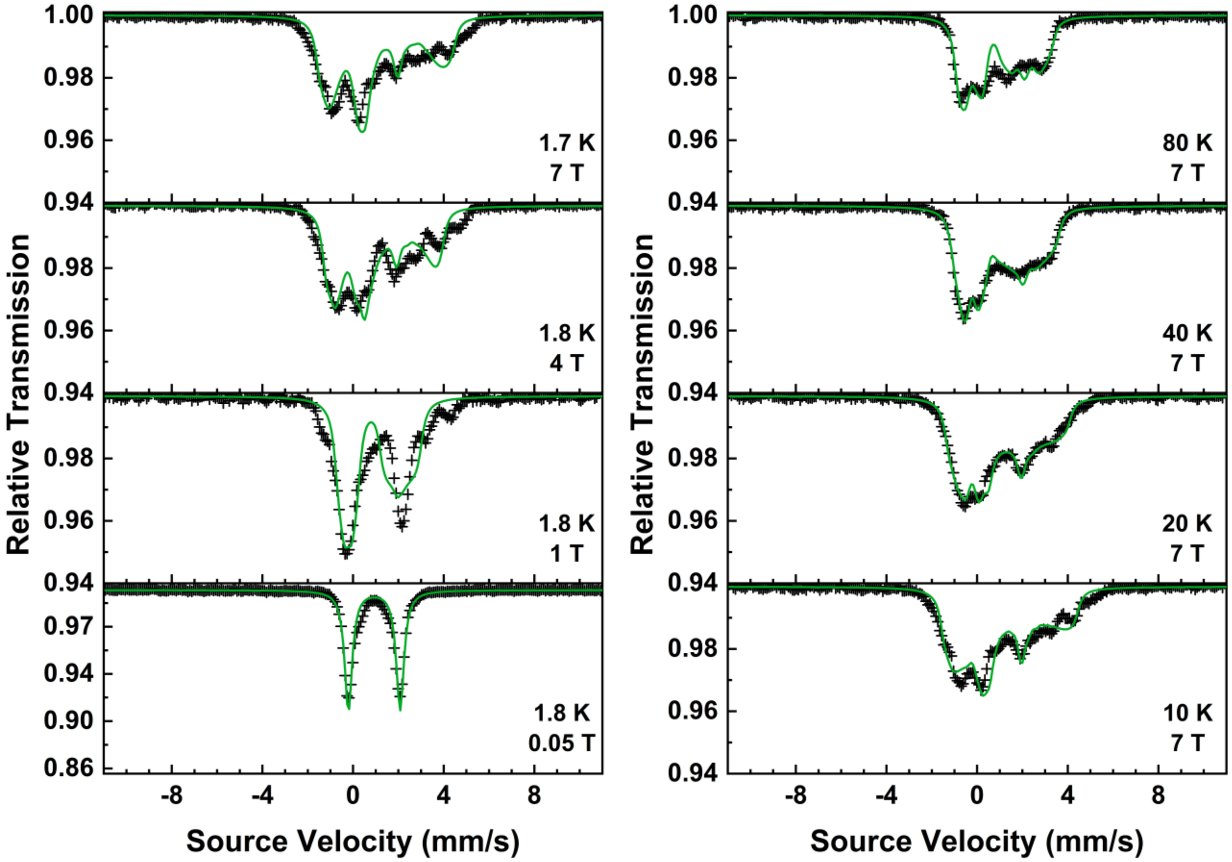

Figure S14. VTVH Mössbauer spectra of **1-Cl** with fit to the data including only the main subspectrum from the fit to the zero-field spectrum (bottom left). (Left): Bottom to top: 0.05 T at 1.8 K, 1 T at 1.8 K, 4 T at 1.8 K, and 7 T at 1.7 K. (Right): Bottom to top: 7 T at 10 K, 7 T at 20 K, 7 T at 40 K, and 7 T at 80 K. The solid green lines correspond to the spin-Hamiltonian fit to the data. Fit parameters are provided in Table S10.

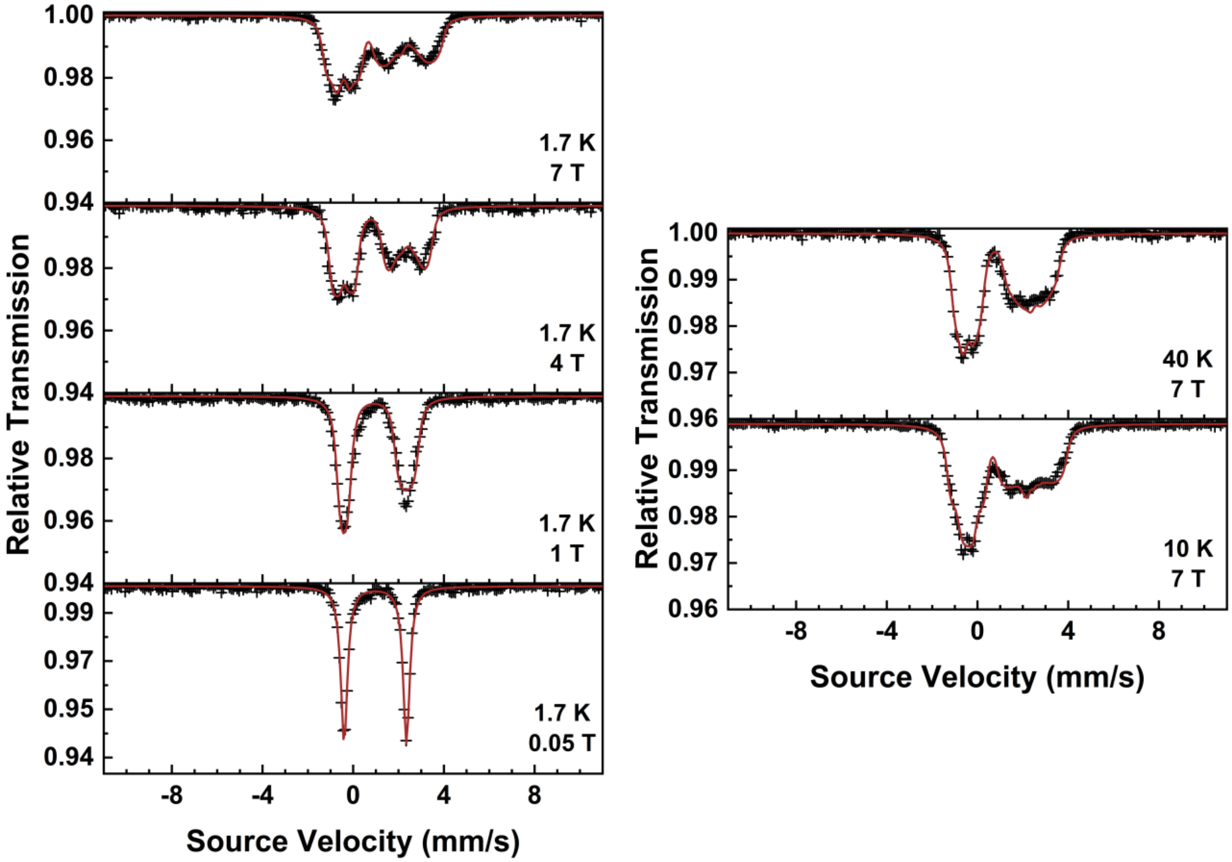

Figure S15. VTVH Mössbauer spectra of **1-Br** with fit to the data. (Left): Bottom to top: 0.05 T, 1 T, 4 T, and 7 T all at 1.7 K. (Right): Bottom to top: 7 T at 10 K and 7 T at 40 K. The solid maroon lines correspond to the spin-Hamiltonian fit to the data. Fit parameters are provided in Table S10.

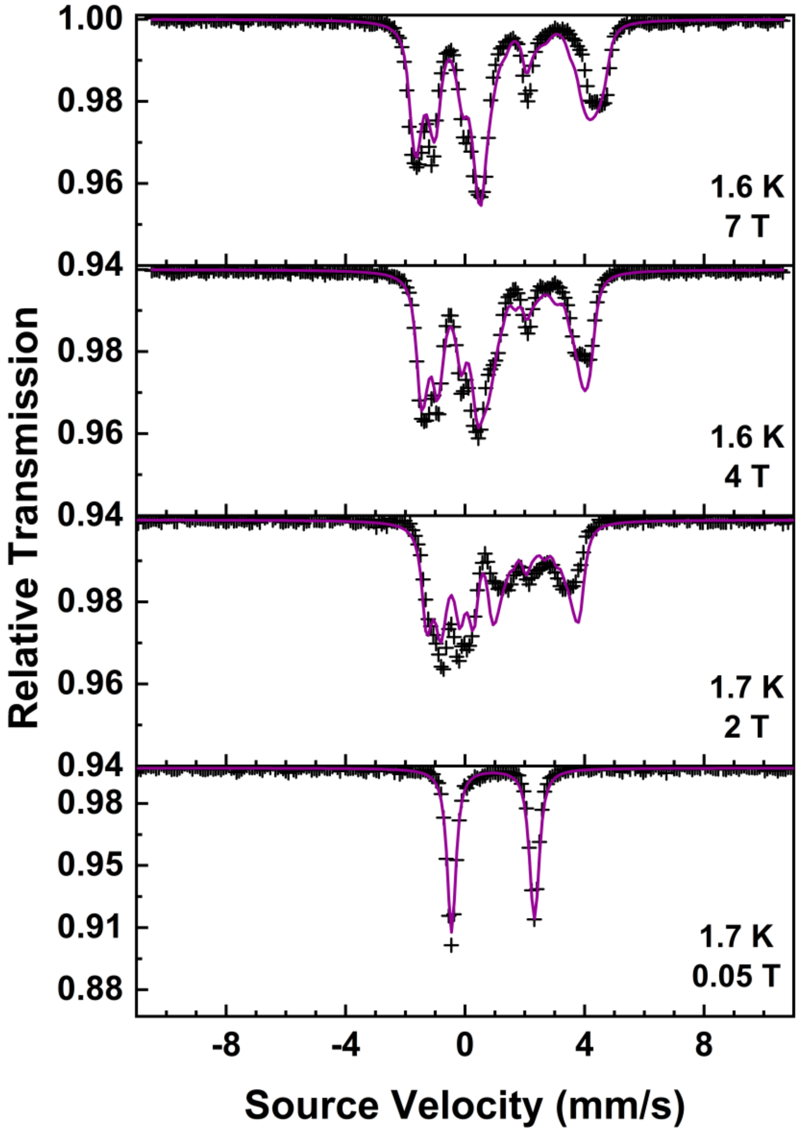

Figure S16. VTVH Mössbauer spectra of **1-I** with fit to the data. Bottom to top: 0.05 T at 1.7 K, 2 T at 1.7 K, 4 T at 1.6 K, and 7 T at 1.6 K. The solid purple lines correspond to the spin-Hamiltonian fit to the data. Fit parameters are provided in Table S10.

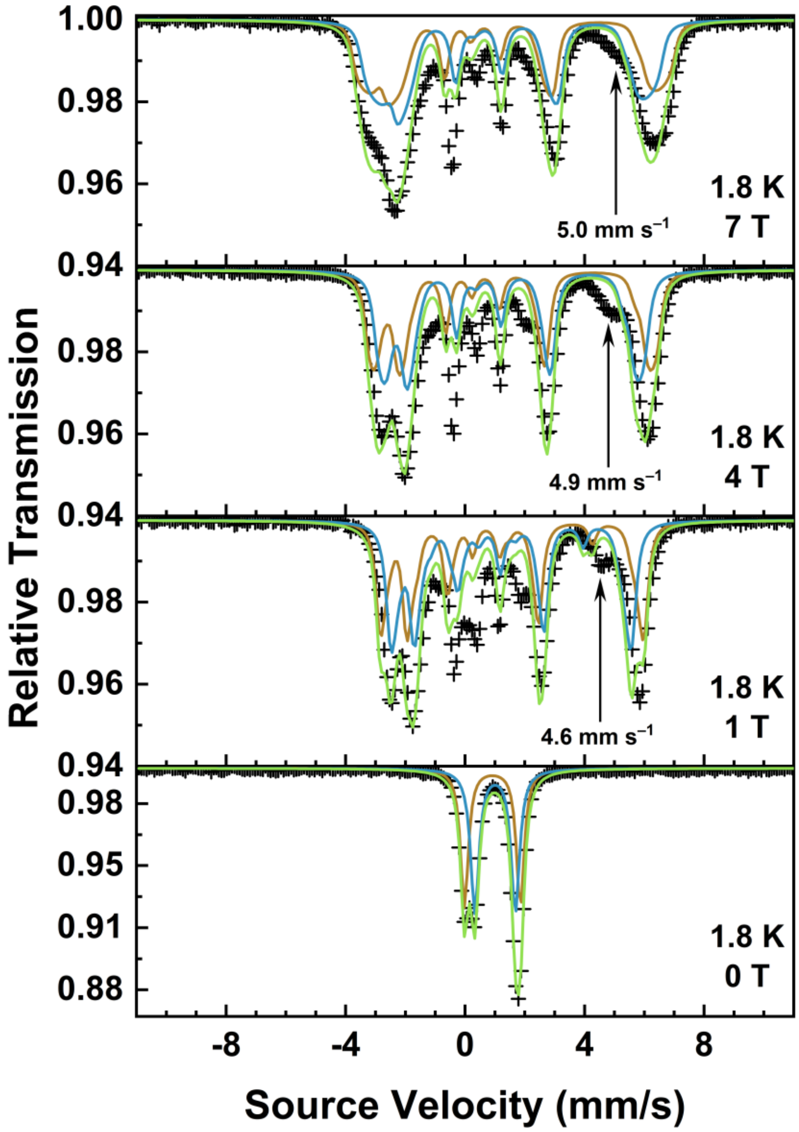

Figure S17. VTVH Mössbauer spectra of **1-F** with alternative fit to the data where the rhombicity parameter was fixed at $E/D = 0.071$ (based on the magnetometry results); both subspectra from fit 2 of the zero-field spectrum (bottom) are included. Bottom to top: 0 T, 1 T, 4 T, and 7 T, all at 1.8 K. The solid lines correspond to the spin-Hamiltonian model, where gold is the first subspectrum, blue the second subspectrum, and maroon the overall fit to the data. The fitted parameters are reported in Table S11. Arrows pointing to the features not captured by the fixed rhombicity parameter are included for clarity.

Supplementary Electronic Structure Calculations Figures:

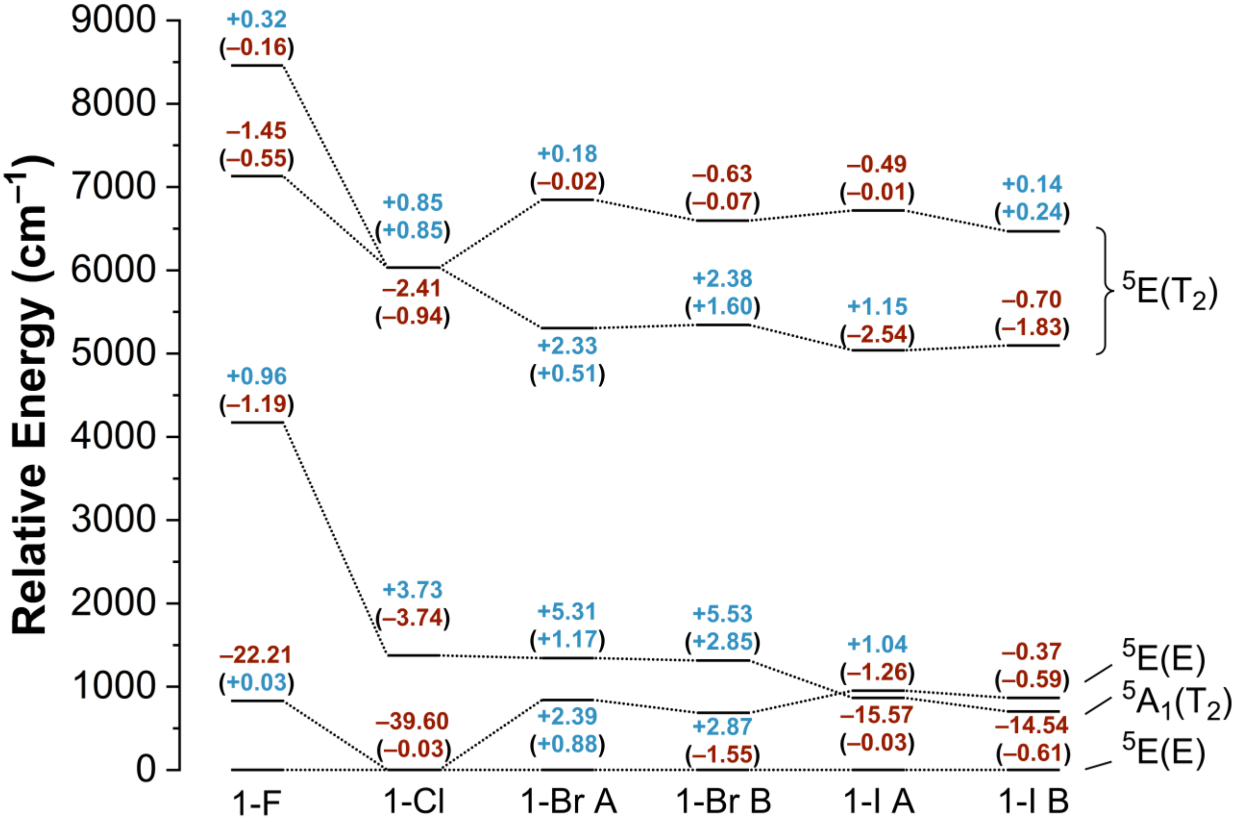

Figure S18. Energies of the five quintet states for each of the **1-X** complexes derived from the CASSCF/NEVPT2 calculations on the H-only optimized geometries. The state labels from C_{3v} symmetry are used merely for accounting (in actuality, the $^5A_1$ state maps to $^5A'$ while the ^5E states map to $^5A'$ and $^5A''$ in C_s symmetry). Note that the energetic ordering of the $^5A_1$ and first ^5E excited state changes for the **1-I** structures compared to the rest of the **1-X** series. The contributions of the four excited quintet states to the $\overleftrightarrow{\boldsymbol{D}}$ tensor are shown, with values outside and inside of parentheses corresponding to contributions to D and E, respectively. Positive contributions are shown in blue, while negative contributions are shown in maroon.

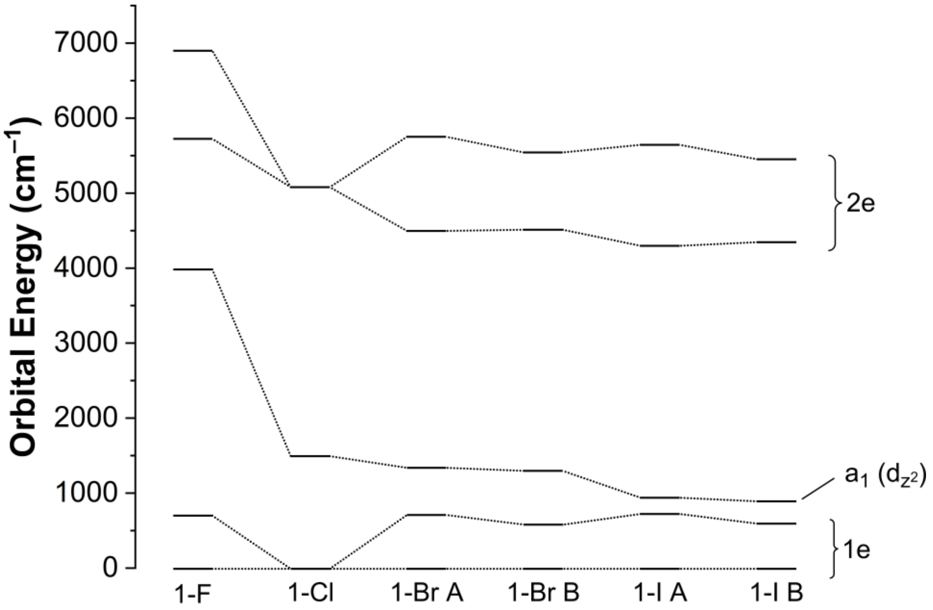

Figure S19. AILFT SALC 3*d*-orbital energies ground ^{5}E state for each of the **1-X** complexes derived from the CASSCF/NEVPT2 calculations on the H-only optimized geometries. The symmetry labels for the C_{3v} point group are used for clarity.

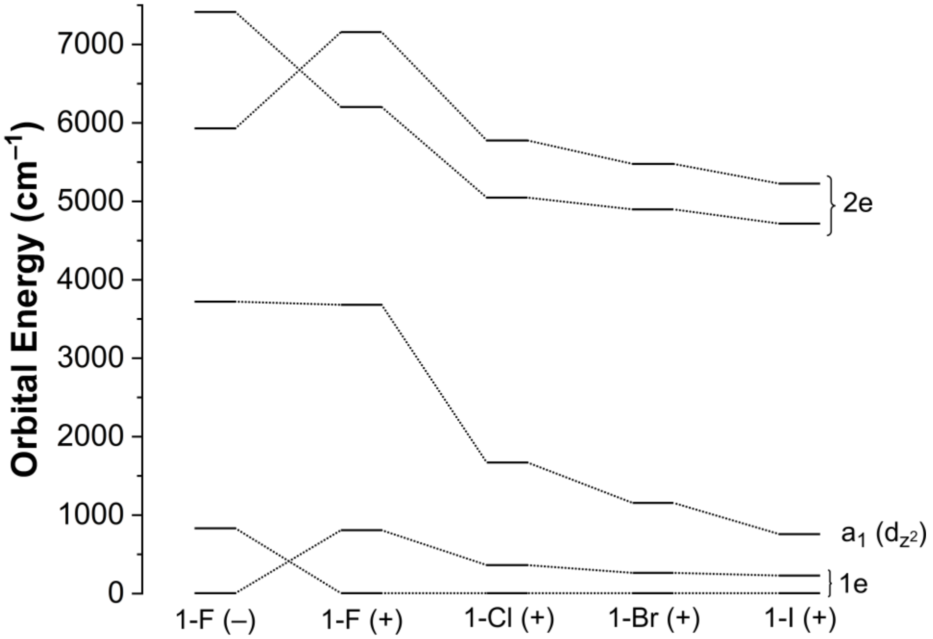

Figure S20. AILFT SALC 3*d*-orbital energies for the ground ^{5}E state for each of the **1-X** complexes derived from the CASSCF/NEVPT2 calculations on the Full Opt geometries. The symmetry labels for the C_{3v} point group are used for clarity.

Supplementary NIR Absorption Spectra Figures:

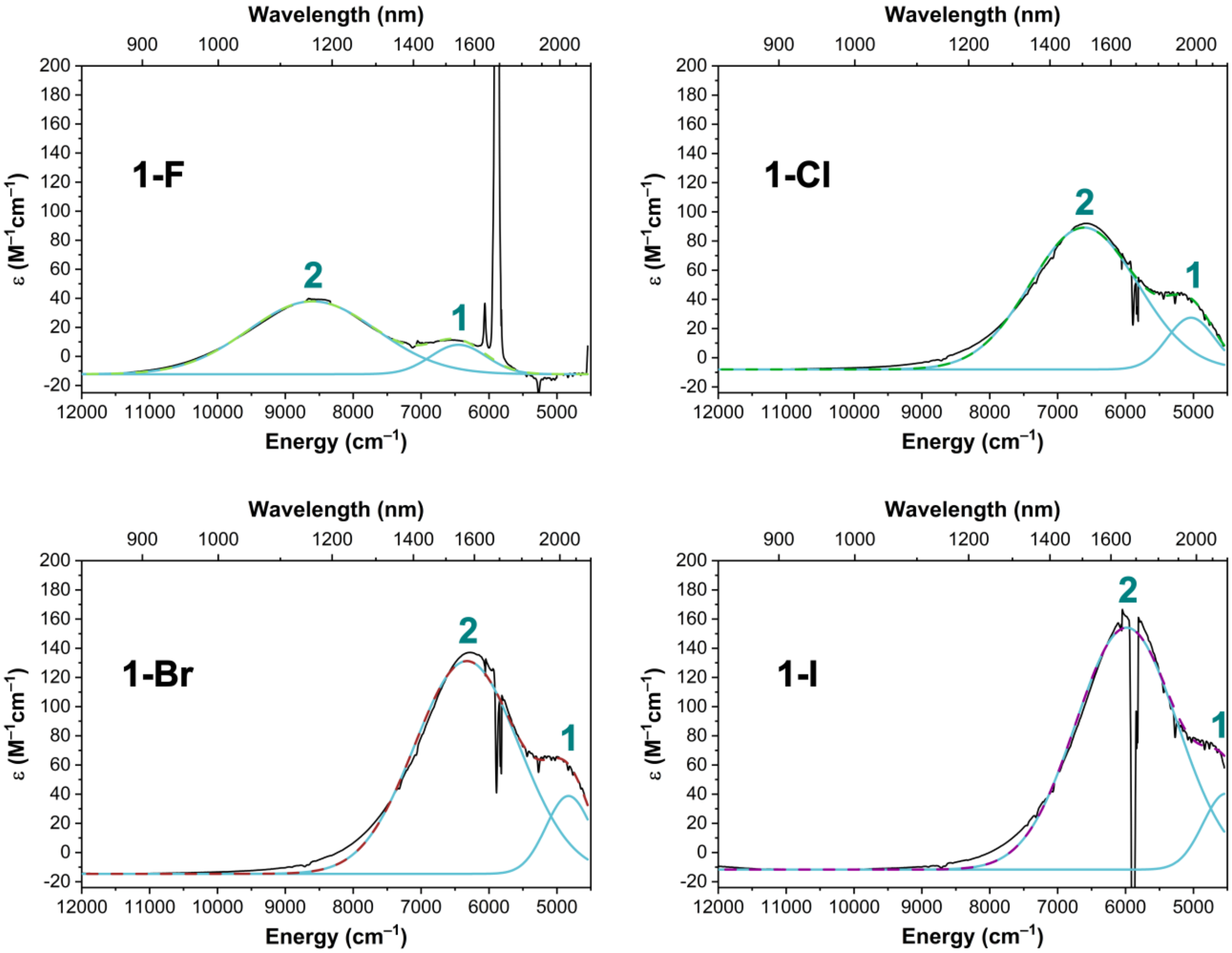

Figure S21. Gaussian deconvoluted NIR absorption spectra of the **1-X** series. Fit parameters for both peaks are given in Table S22.

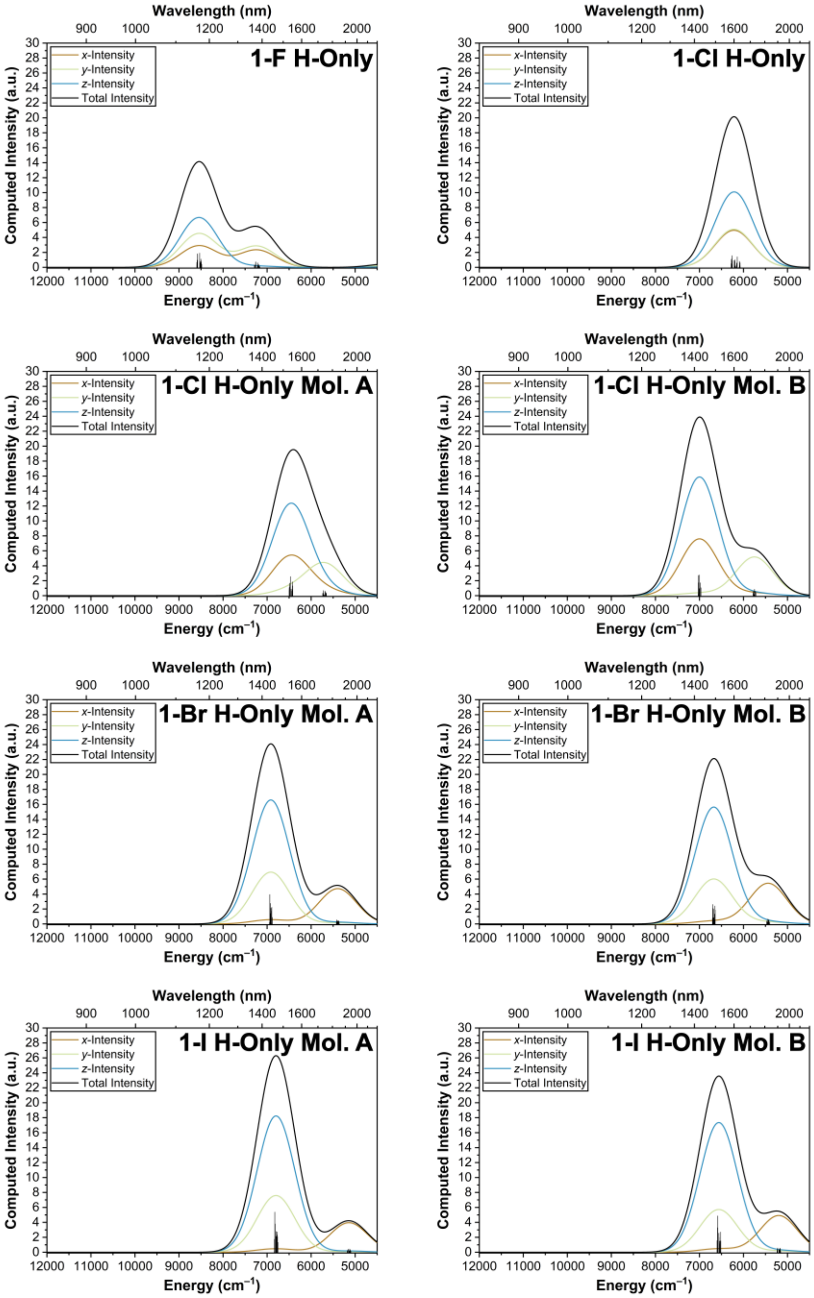

Figure S22. Calculated spin-orbit corrected NIR absorption spectra for the **1-X** series from the CASSCF/NEVPT2 calculations on the H-only optimized geometries. The calculated $^5E(T_2)$ bands are split into $^5A'$ and $^5A''$ bands due to the descent in molecular symmetry from C_{3v} to C_s in each complex except for **1-Cl**, where the crystallographic $R3m$ symmetry imposes an idealized C_{3v} symmetry leading to a single ^5E band (Scheme 2). The H-only geometries for both molecules of the previously-reported data[1] for **1-Cl** are also included.

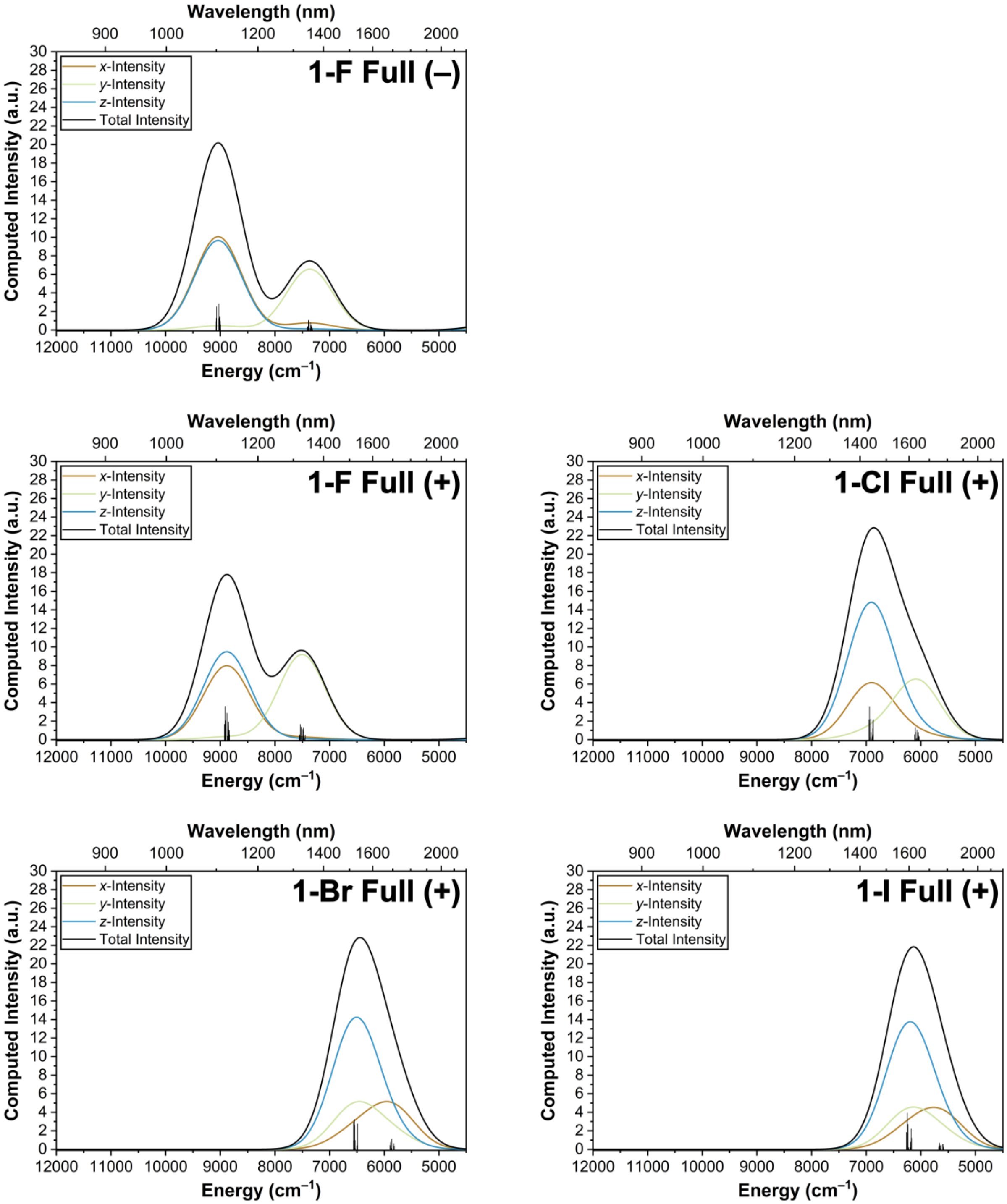

Figure S23. Calculated spin-orbit corrected NIR absorption spectra for the **1-X** series from the CASSCF/NEVPT2 calculations on the Full Opt (+) geometries (as well as the Full Opt (–) geometry for **1-F**). The calculated $^{5}E(T_2)$ bands are split into $^{5}A'$ and $^{5}A''$ bands due to the descent in molecular symmetry from C_{3v} to C_s in each complex (Scheme 2), which is most apparent as a separate peak for **1-F**, while showing up as a shoulder in the main band for **1-Cl**, **1-Br**, and **1-I**.

Experimental and Computational Methods:

General Synthetic Procedures. All solvents and reagents were purchased from commercial suppliers and not further purified (if not otherwise noted). Solvents were dried over sodium/benzophenone or CaH_2, followed by distillation and storage over activated molecular sieves. All Fe(II) complexes were prepared and handled in a Vacuum Atmospheres Omni-Lab glovebox under an inert nitrogen atmosphere. K($Tp^{tBu,Me}$) was prepared using an established procedure.[2] Complexes with the general formula [Fe^{II}X($Tp^{tBu,Me}$)] (**1-X**; X = Cl, Br, I) were prepared using a slightly modified version of the procedure reported by Theopold et al.[1] Equimolar solutions of K($Tp^{tBu,Me}$) (463 mg, 1.0 equiv) and FeX_2 (1.0 equiv) in THF were combined at room temperature and stirred overnight. After filtration and removal of solvent under vacuum, the resulting solid was washed five times with diethyl ether to remove impurities (the washing process involved stirring the solid in 30 mL of diethyl ether for 15 minutes, followed by filtration) before finally being dried. The isolated solid was then dissolved in 5.0 mL of CH_2Cl_2 and filtered again. After layering with 15 mL of pentane, small amounts of white precipitate formed upon sitting overnight at room temperature. The mother liquor was decanted and stored at –30 °C to generate X-ray quality crystals of the desired Fe(II) complexes. The crystals were collected and dried under vacuum to provide material for magnetometry and spectroscopic studies. Sample purity was confirmed using ^{1}H NMR and Mössbauer spectroscopies. For sample **1-F**, the purity was assessed further by elemental analysis (collected at Midwest Microlab, LLC, in Indianapolis, IN).

[Fe^{II}Cl($Tp^{tBu,Me}$)] (1-Cl). Yield = 144 mg (28%). ^{1}H NMR (400 MHz, $CDCl_3$): δ = 65.03 (s, 3H, 4-*H*-pz), 44.37 (br s, 1H, B-*H*), 39.40 (s, 9H, 5-Tp-CH_3), –8.99 ppm (s, 27H, –C(CH_3)$_3$).

[Fe^{II}Br($Tp^{tBu,Me}$)] (1-Br). Yield = 112 mg (20%). ^{1}H NMR (400 MHz, $CDCl_3$): δ = 66.50 (s, 3H, 4-*H*-pz), 34.85 (s, 9H, 5-Tp-CH_3), 20.28 (br s, 1H, B-*H*), –0.22 ppm (s, 27H, –C(CH_3)$_3$).

[Fe^{II}I($Tp^{tBu,Me}$)] (1-I). Yield = 181 mg (30%). ^{1}H NMR (400 MHz, $CDCl_3$): δ = 67.75 (s, 3H, 4-*H*-pz), 29.00 (s, 9H, 5-Tp-CH_3), 11.90 (s, 27H, –C(CH_3)$_3$), –12.23 ppm (br s, 1H, B-*H*).

[Fe^{II}F($Tp^{tBu,Me}$)] (1-F). *Warning: thallium compounds should be handled with extreme care due to their inherent toxicity, and proper PPE is an absolute necessity.*

Thallium fluoride (106 mg, 0.45 mmol, 2.5 equiv) was added to a solution of **1-Cl** (94 mg, 0.18 mmol, 1.0 equiv) in toluene (10 mL). The solution was then filtered after being allowed to stir overnight to remove the solvent, from which the remaining isolated residue was washed five times with diethyl ether to remove impurities (the washing process involved stirring the solid in

30 mL of diethyl ether for 15 minutes, followed by filtration) before finally being dried. The isolated solid was then dissolved in 5.0 mL of CH_2Cl_2 and filtered again. After layering with 15 mL of pentane, small amounts of white precipitate formed upon sitting overnight at room temperature. The mother liquor was decanted and stored at –30 °C to generate X-ray quality crystals. The crystals were collected and dried under vacuum. Yield = 67 mg (74%). ^{1}H NMR (400 MHz, $CDCl_3$): δ = 76.63 (br s, 1H, B-*H*), 61.00 (s, 3H, 4-*H*-pz), 42.19 (s, 9H, 5-Tp-CH_3), –19.44 (s, 27H, −C(CH_3)$_3$). Anal. Calc. for $C_{24}H_{40}BFFeN_6$: C, 57.85; H, 8.09; N, 16.87. Found: C, 56.16; H, 7.56; N, 15.39. The observed discrepancies between computed and experimental values is due to a small amount of Tl(Tp^{tBu,Me}) impurity. The diamagnetic correction inferred from magnetometry was 13.78 mole %, which if due *entirely* to the presence of Tl(Tp^{tBu,Me}) would give: C, 56.20; H, 7.86; N, 16.39, in better agreement with the found values.

X-ray Crystallography. Single-crystal X-ray diffraction data for the **1-X** series were collected using an Oxford Diffraction SuperNova kappa-diffractometer (Rigaku Corp.) equipped with both Cu and Mo K_α X-ray sources. X-rays were detected using an Atlas CCD detector, and the crystal temperature was maintained at 100 K with an open-flow Cryojet LN_2 cooling device (Oxford Instruments). Structures were solved using the SHELXS program[3, 4] and refined with the SHELXL program found within the Olex2 crystallographic package.[5] Details concerning the data collection, analysis, and crystallographic parameters are provided in Tables S1 and S2. Crystallographic data (CIF format) can be obtained from the Cambridge Crystallographic Data Centre under the following deposition numbers 2443125 (**1-F**), 2443126 (**1-Cl**), 2443127 (**1-Br**), and 2443128 (**1-I**).

Magnetometry Measurements. Variable-temperature direct current paramagnetic susceptibility and variable-temperature/-field (VTVH) magnetization data for each complex were measured in the Joint Workspace of the Max Planck Institute for Chemical Energy Conversion (MPI-CEC) and the Max-Planck-Institut für Kohlenforschung (MPI-KoFo) with a MPMS 3 Quantum Design SQUID magnetometer. Samples for each compound were prepared in flame-sealed NMR tubes containing ~10-20 mg of a given complex. For the **1-F** and **1-I** samples, orientation effects were observed in initial measurements, so ~20 mg of *n*-eicosane was also added to the NMR tubes and subsequently melted after flame sealing. The diamagnetic susceptibilities of the sample tubes were accounted for by measuring empty NMR tubes (or flame-sealed NMR-

tubes containing 20 mg of *n*-eicosane for **1-F** and **1-I**) under the same conditions, and subtracted from the measured susceptibilities of the samples during data processing.

Experimental data were corrected for intrinsic diamagnetism using Pascal's constants.[6] It should be noted that the value for λ(imidiazole) was used in the calculation for $\chi_{\mathrm{D}}(\mathrm{Tp})$ because λ(pyrazole) is not a tabulated value in the literature.

- Diamagnetic Susceptibility of $(\mathrm{Tp}^{\mathrm{tBu,Me}})^{-}$ Ligand:

$$\chi_{\mathrm{D}}(\mathrm{Tp}) = 3\left[3\chi_{\mathrm{D}}(\mathrm{C_{ring}}) + 2\chi_{\mathrm{D}}(\mathrm{N_{ring}}) + 5\chi_{\mathrm{D}}(\mathrm{C}) + 13\chi_{\mathrm{D}}(\mathrm{H}) + \lambda(\mathrm{imidiazole})\right] + \chi_{\mathrm{D}}(\mathrm{B}) + \chi_{\mathrm{D}}(\mathrm{H}) = -274.02 \cdot 10^{-6}\ \mathrm{cm^3\ mol^{-1}}$$

- Diamagnetic Susceptibility of **1-F**:

$$\chi_{\mathrm{D}} = \chi_{\mathrm{D}}(\mathrm{Tp}) + \chi_{\mathrm{D}}(\mathrm{Fe^{2+}}) + \chi_{\mathrm{D}}(\mathrm{F^{-}}) = -296.12 \cdot 10^{-6}\ \mathrm{cm^3\ mol^{-1}}$$

- Diamagnetic Susceptibility of **1-Cl**:

$$\chi_{\mathrm{D}} = \chi_{\mathrm{D}}(\mathrm{Tp}) + \chi_{\mathrm{D}}(\mathrm{Fe^{2+}}) + \chi_{\mathrm{D}}(\mathrm{Cl^{-}}) = -310.42 \cdot 10^{-6}\ \mathrm{cm^3\ mol^{-1}}$$

- Diamagnetic Susceptibility of **1-Br**:

$$\chi_{\mathrm{D}} = \chi_{\mathrm{D}}(\mathrm{Tp}) + \chi_{\mathrm{D}}(\mathrm{Fe^{2+}}) + \chi_{\mathrm{D}}(\mathrm{Br^{-}}) = -321.62 \cdot 10^{-6}\ \mathrm{cm^3\ mol^{-1}}$$

- Diamagnetic Susceptibility of **1-I**:

$$\chi_{\mathrm{D}} = \chi_{\mathrm{D}}(\mathrm{Tp}) + \chi_{\mathrm{D}}(\mathrm{Fe^{2+}}) + \chi_{\mathrm{D}}(\mathrm{I^{-}}) = -337.62 \cdot 10^{-6}\ \mathrm{cm^3\ mol^{-1}}$$

All data processing was done using mpView version 1.4.6 (written by Dr. Eckhard Bill and available by email to: daniel.santalucia@cec.mpg.de).[7]

Each sample was cooled to 2 K in the absence of an external applied magnetic field, and the magnetic moment was then measured subsequently in logarithmic increments up to 300 K in an external applied 10 kOe magnetic field, with the exception of **1-F**, whereby susceptibility data were obtained by measuring the magnetic moment from 300 K to 2 K in logarithmic increments in an applied 10 kOe magnetic field. Due to slight deviations in the $\chi_{\mathrm{P}}T$ vs. T datapoints from 65-119 K as well as at 300 K for **1-F**, these datapoints were excluded from modeling. VTVH data were collected for each complex at isofields of 10, 40, and 70 kOe while increasing the temperature from 2 to 300 K in increments with even spacing in $1/T$.

The susceptibility and VTVH data for each complex were modeled simultaneously using JulX20 version 1.4 (written by Dr. Eckhard Bill and available by email to: daniel.santalucia@cec.mpg.de).[8] All data were modeled by diagonalizing the spin-Hamiltonian, which is given by:

$$\hat{H} = \mu_B \vec{S} \overleftrightarrow{\boldsymbol{g}} \vec{B} + \vec{S} \overleftrightarrow{\boldsymbol{D}} \vec{S} = \mu_B \vec{S} \overleftrightarrow{\boldsymbol{g}} \vec{B} + D \left[\vec{S_z}^2 - \frac{1}{3} S(S+1) \right] + E \left[\vec{S_x}^2 - \vec{S_y}^2 \right]$$

where μ_B is the Bohr magneton, $\vec{B}$ is the applied external magnetic field, and $\vec{S_j}$, $\overleftrightarrow{\boldsymbol{g}}$, and $\overleftrightarrow{\boldsymbol{D}}$ are the (effective) spin vector ($j = x$-, y-, and z-directions), electron g-tensor, and zero-field splitting (ZFS) tensors (parameterized with the axial and rhombic ZFS parameters D and E, respectively; the rhombicity parameter is defined such that $0 \leq E/D \leq 0.333$), respectively; $S = 2$. All compounds were modeled with rhombic $\overleftrightarrow{\boldsymbol{g}}$ ($g_x \neq g_y \neq g_z$) and nearly rhombic $\overleftrightarrow{\boldsymbol{D}}$ ($D = \frac{3}{2} D_z$; $E/D = \frac{1}{3} \frac{(D_x - D_y)}{D_z} \approx 0.333$) tensors except for **1-F**, which was modeled using an axial $\overleftrightarrow{\boldsymbol{g}}$-tensor ($g_z = g_\parallel$; $g_x = g_y = g_\perp$) and a $\overleftrightarrow{\boldsymbol{D}}$-tensor with only mild rhombicity ($E/D = 0.071$). Temperature-independent paramagnetism (TIP)[9] was used in the spin-Hamiltonian models for the magnetometry data for each compound in order to correct the values at high temperatures. The models of the susceptibility and VTVH data also included a percentage of a diamagnetic impurity (except for **1-Cl**), around 9-16 mol %, which was necessary to arrive at a consistent set of parameters between both datasets for each compound. The origins of these diamagnetic impurities are likely due to imperfect diamagnetic corrections of the *n*-eicosane (when applicable) and/or the sample holders as well as, in the case of **1-F**, to the presence of residual reactant Tl($Tp^{tBu,Me}$).

HFEPR/FIRMS Measurements. HFEPR spectra were collected at frequencies of 70-641 GHz with fields up to 14.9 T at temperatures of 6-10 K using a transmission spectrometer, described in its original configuration by Hassan et al.,[10] based on a 15/17-T warm-bore superconducting magnet (Oxford Instruments). The microwaves were produced by a phase-locked generator (8-20 GHz) followed by a chain of frequency multipliers (Virginia Diodes Inc.). FIRMS data were measured using a Bruker Vertex 80v FTIR spectrometer coupled with a 17 T vertical-bore superconducting magnet in a Voigt configuration (light propagation perpendicular to the applied field). Broadband THz radiation emitted by a Hg arc lamp was transmitted through the sample and detected by a composite silicon bolometer (Infrared Laboratories) mounted at the end of the quasi-optical transmission line. Both the sample and bolometer were cooled by low-pressure helium gas to a temperature of 5.5 K. Each sample was fixated with n-eicosane to prevent orientation effects. Each sample was measured in the region between ~10-120 cm^{-1} (0.30-3.6 THz) with a resolution of 0.3 cm^{-1} (9 GHz) with applied fields up to 17.5 T. All spectra were normalized by dividing by the reference spectrum, which is the average spectrum collected at all magnetic

fields, allowing magnetic absorptions and nonmagnetic vibrational features to be distinguished from one another.

Analysis of the FIRMS data was performed by calculating the energies of the M_S levels (from the spin-Hamiltonian) and subsequently simulating the turning point branches in the *x*-, *y*-, and *z*-directions and comparing their agreement with the FIRMS data. Simulations of the HFEPR turning points for **1-Cl** and **1-Br** were conducted using the program SPIN (written by Andrew Ozaroswki and available by email to: ozarowsk@magnet.fsu.edu). The spin Hamiltonian used for simulating the HFEPR spectra is given by:

$$\hat{H} = \mu_{\mathrm{B}}\vec{S}\,\overleftrightarrow{\boldsymbol{g}}\,\vec{B} + D\left[\vec{S}_z^{\,2} - \frac{1}{3}S(S+1)\right] + E\left[\vec{S}_x^{\,2} - \vec{S}_y^{\,2}\right] + B_4^0\hat{O}_4^0 + B_4^3\hat{O}_4^3$$

where μ_{B} is the Bohr magneton, $\vec{S}_j$ is the (effective) spin vector (j = *x*-, *y*-, and *z*-directions), $\overleftrightarrow{\boldsymbol{g}}$ is the electron g-tensor, $\vec{B}$ is the applied external magnetic field, and the zero-field splitting (ZFS) tensor is parameterized with the axial and rhombic ZFS parameters D and E, respectively (the rhombicity parameter is defined such that $0 \leq E/D \leq 0.333$), as well as the fourth-order ZFS parameters B_4^0 and B_4^3 appropriate for trigonal symmetry, where $\hat{O}_4^0 = 35\vec{S}_z^{\,4} - 30\,S(S+1)\vec{S}_z^{\,2} + 25\vec{S}_z^{\,2} - 6S(S+1) + 3S^2(S+1)^2$ and $\hat{O}_4^3 = \frac{1}{2}\left[\vec{S}_z\left(\vec{S}_x^{\,3} - 3\vec{S}_x\vec{S}_y^{\,2}\right) + \left(\vec{S}_x^{\,3} - 3\vec{S}_x\vec{S}_y^{\,2}\right)\vec{S}_z\right]$ are their corresponding Stevens operators; $S = 2$. The $B_4^3\hat{O}_4^3$ term proved unnecessary since it did not improve fits to the data, and was thus excluded from the models.

^{57}Fe Mössbauer Spectroscopy. In the Joint Workspace of the Max Planck Institute for Chemical Energy Conversion (MPI-CEC) and the Max-Planck-Institut für Kohlenforschung (MPI-KoFo), both zero-field and variable-temperature/-field (VTVH) Mössbauer spectra were measured using a Mössbauer spectrometer employing a constant acceleration Doppler drive (in-house construction) with an attached ^{57}Co/Rh (50 mCi, i.e., 1.85 GBq) γ-ray source. The source was kept at room temperature while situated inside the magnetic coils via a reentrant tube, and adjusted to be ~9 cm away (where there were nearly zero residual magnetic field lines) from the Mössbauer sample. The sample was kept at a constant temperature in a mini Cryogen-Free Measurement System (Cryogenic Ltd.) closed-cycle cryostat in conjunction with a RP-082B2S 4 Kelvin pulse tube cryocooler and cold head (Sumitomo Heavy Industries Ltd.). The mini Cryogen-Free Measurement System features a split pair superconducting 7-Tesla magnet, and includes an integrated top-loading variable-temperature insert. The cryostat allows for sample temperatures in

the range of 1.6-80 K to be achieved. The Doppler drive was oriented in a configuration relative to the magnetic coils such that the external magnetic field was perpendicular to the γ-rays. The γ-ray detector was a Vitus H150 Si drift detector (170 mm^2 collimated to 150 mm^2) from the AXAS-M system with 512 channels (Ketek GmbH). The detector was mounted to the tip of a 200 mm diameter stainless-steel finger that formed an airtight seal inside the magnet in the cryostat such that it was in close proximity to the Mössbauer sample where the γ-ray beam had a wider aperture.

The spectra were collected in the velocity range of ±11 mm/s (with the exceptions of the 1.6 K 4 & 7 T spectra for **1-I**, which had a velocity range of ±10.5 mm/s); all spectra were calibrated to 12.5 μm α-Fe foil at 298 K. The minimum experimental linewidth was 0.25 mm/s based on the observed minimum linewidths in the six-line calibration spectra. The zero-field Mössbauer spectra were modeled with quadrupole doublets with Lorentzian linewidths (i.e., assuming the thin-absorber approximation) with the in-house software mf2 version 2.7.3 (written by Eckhard Bill and available by email to: daniel.santalucia@cec.mpg.de),[11] while the VTVH spectra were simulated with subspectra with Lorentzian linewidths for each subspectrum using the in-house software mx version 1.8.4 (written by Eckhard Bill and available by email to: daniel.santalucia@cec.mpg.de).[12] The spin-Hamiltonian was used for modeling all subspectra, and is given by:

$$\hat{H} = \mu_{\mathrm{B}}\vec{S}\,\overleftrightarrow{\boldsymbol{g}}\,\vec{B} + \vec{S}\,\overleftrightarrow{\boldsymbol{D}}\,\vec{S} + \vec{S}\,\overleftrightarrow{\boldsymbol{A}}\,\vec{I} - g_{\mathrm{N}}\mu_{\mathrm{N}}\vec{I}\cdot\vec{B} + \vec{I}\,\overleftrightarrow{\boldsymbol{Q}}\,\vec{I}$$

$$= \mu_{\mathrm{B}}\vec{S}\boldsymbol{g}\vec{B} + D\left[\vec{S}_z^{\,2} - \frac{1}{3}S(S+1)\right] + E\left[\vec{S}_x^{\,2} - \vec{S}_y^{\,2}\right] - g_{\mathrm{N}}\mu_{\mathrm{N}}\left(\frac{-\langle\vec{S}\rangle_i\,\overleftrightarrow{\boldsymbol{A}}}{g_{\mathrm{N}}\mu_{\mathrm{N}}} + \vec{B}\right)\cdot\vec{I}$$

$$+ \frac{eQV_{zz}}{4I(2I-1)}\left[3\vec{I}_z^{\,2} - I(I+1) + \eta\left(\vec{I}_x^{\,2} - \vec{I}_y^{\,2}\right)\right]$$

where μ_{B} is the Bohr magneton, $\vec{S}_j$ is the (effective) spin vector (j = *x*-, *y*-, and *z*-directions), $\overleftrightarrow{\boldsymbol{g}}$ is the electron g-tensor, $\vec{B}$ is the applied external magnetic field, $\overleftrightarrow{\boldsymbol{D}}$ is the zero-field splitting (ZFS) tensor (parameterized with the axial and rhombic ZFS parameters D and E, respectively; the rhombicity parameter is defined such that $0 \leq E/D \leq 0.333$), $S = 2$, $\overleftrightarrow{\boldsymbol{A}}$ is the electron-nuclear hyperfine coupling tensor (which can be modeled axially, where $A_{xx} = A_{yy} \neq A_{zz}$, or rhombically, where $A_{xx} \neq A_{yy} \neq A_{zz}$), g_{N} is the nuclear g-factor, μ_{N} is the nuclear magneton, $\vec{I}_i$ is the nuclear spin vector (i = *x*-, *y*- and *z*-directions), $\overleftrightarrow{\boldsymbol{Q}}$ is the quadrupole interaction tensor, $I = \frac{3}{2}$, $\frac{-\langle\vec{S}\rangle_i\overleftrightarrow{\boldsymbol{A}}}{g_{\mathrm{N}}\mu_{\mathrm{N}}}$ is the internal field at the nucleus (which incorporates the individual spin-expectation

values $\langle \vec{S} \rangle_i$ for each electron spin vector (for $i = 1, \ldots, 2S + 1$), which effectively decouples the electronic and nuclear parts of the Hamiltonian from one another), e is the elementary charge of a proton, Q is the nuclear quadrupole moment, V_{zz} is the main/z-component of the electric-field gradient (efg) tensor, and η is the asymmetry parameter (defined as $\eta = \frac{V_{xx} - V_{yy}}{V_{zz}}$, where V_{xx} and V_{yy} are the x- and y-components of the efg tensor, respectively; the efg components are defined such that $0 \leq \eta \leq 1$). The β Euler angle for the efg tensor was also modeled for **1-F**. The isomer shift was not modeled with the spin-Hamiltonian because it shifts all energies by the same amount.[13] The errors in the fitted spin-Hamiltonian parameters were estimated based on their ability to substantially impact the models to the spectra.[14] All VTVH Mössbauer spectra collected at 1.6-1.8 K were modeled with slow relaxation, while the spectra at higher temperatures (10, 20, 40, & 80 K for **1-Cl** and 10 & 40 K for **1-Br**) were modeled in the fast relaxation regime.[13]

To prevent overparameterization, the spin-Hamiltonian parameters derived from the zero-field Mössbauer spectra and from the models to the magnetometry or HFEPR/FIRMS data (i.e., δ, $|\Delta E_{\mathrm{Q}}|$, Γ, g_x, g_y, g_z, D, and E/D) were used as fixed values, while the parameters describing the electron-nuclear hyperfine coupling were allowed to refine freely. This is further justified because, in general, Mössbauer data are not very sensitive to electron g-values, and also because the zero-field splitting parameters determined by Mössbauer spectroscopy generally have errors of about 15-20%.[13] Therefore, fixing these parameters allowed for more precise determinations of the fitted hyperfine coupling tensor components. The parameters obtained from modeling the magnetometry data were used as fixed parameters for modeling the VTVH Mössbauer data of **1-F** and **1-I**, while the parameters obtained from modeling the HFEPR/FIRMS data were used as fixed parameters for modeling the VTVH Mössbauer data of **1-Cl** and **1-Br**. This was done because the parameters obtained by modeling HFEPR/FIRMS data generally are more precise and accurate than those obtained by modeling magnetometry alone, while parameters obtained by modeling magnetometry data are more precise than those obtained by modeling FIRMS alone. The rhombicity parameter, E/D, for **1-F** was allowed to refine freely, because the feature at ~4.6, 4.9, and 5.0 mm s^{-1} in the 1, 4, and 7 T spectra, respectively, is only captured when the rhombicity is 0.10-0.11. An alternative fit where the rhombicity parameter for **1-F** was not allowed to refine freely and instead fixed at 0.071 (the value obtained from fitting the magnetometry data) is shown in Figure S17, with fit parameters reported in Table S11.

Although the physical origins of η and the rhombicity parameter E/D are different (η comes from the ground state[13] while ZFS for transition metals primarily originates from spin-orbit coupling to excited states with multiplicities the same as or differing by ±1 relative to the ground state[15]), it was reasoned that they both are related to the electronic symmetries for these systems, and hence that they should coincide with one another (i.e. for rhombic systems, η should be closer to the limit of 1, while for axial systems it should be near 0). Thus, in most cases, the value for η was also fixed, either to 0 to coincide with the mildly rhombic electronic symmetry of **1-F**, or to 1 to coincide with the near-rhombic electronic symmetric of **1-Br** and **1-I**. However, the value for η was allowed to freely refine for **1-Cl**. The sign of ΔE_{Q} was determined for **1-F** and **1-Cl**, whereas the sign is irrelevant for **1-Br** and **1-I** since they were modeled at the limit of $\eta = 1$.

The electron-nuclear hyperfine coupling tensor, $\overleftrightarrow{\boldsymbol{A}}$, was modeled axially for **1-F**, and rhombically for **1-Cl**, **1-Br**, and **1-I**, consistent with the electronic symmetries of each system. As explained below, certain components of $\overleftrightarrow{\boldsymbol{A}}$ were fixed at –21.0 T depending on the sign of D. In general, for systems with a negative D-parameter (e.g. **1-F**), fits are more sensitive to A_{zz} than A_{xx} and A_{yy} due to the easy-axis of magnetization; for systems with easy-plane magnetization, the data are instead more sensitive to A_{xx} and A_{yy} than to A_{zz}. Thus, for **1-F**, the values of A_{xx} and A_{yy} were fixed to be the value typically obtained from the Fermi contact contribution to the hyperfine tensor (typically about –21.0 T for high-spin Fe(III) and Fe(II) complexes[16]), whereas instead the value for A_{zz} was fixed for **1-Br**. It was found that the value for A_{zz} for **1-Cl** also needed to be fixed because it tended to inflate to unreasonably large values if allowed to fit freely. For **1-I**, all three values were allowed to refine freely, though the data were nonetheless most sensitive to perturbations in A_{zz}, as expected for a negative D-parameter.

In general, the fits to the Mössbauer data capture the overall intensities of the experimental data relatively well, and the peak positions show that the fits to the data correctly capture the energetic perturbations of the system. The exception for this is the model to the data for **1-Cl**, where multiple peaks observed experimentally are not captured by the model. This is easily explained by a couple different effects. For the zero-field Mössbauer data, two quadrupole doublets (corresponding to two separate species) were modeled to the data. In the VTVH model, only the main subspectrum was used, since the hyperfine splitting for the species corresponding to only 15% of the total peak area was difficult to model due to it splitting into relatively low intensity

peaks, many of which are obfuscated by the peaks of the main subspectrum. Although not all of the peaks are modeled, the peak positions and intensities for the major component are accounted for properly. Additionally, the two Jahn-Teller geometries could lead to a distribution of electron-nuclear hyperfine coupling parameters and, hence, a distribution of internal fields, leading ultimately to a distribution in magnetic splittings, none of which were able to be accounted for in this model.

Thus, the set of spin-Hamiltonian parameters derived from fitting the magnetometry and HFEPR/FIRMS data for the **1-X** series are compatible with and able to adequately model the VTVH Mössbauer data, allowing for precise determinations of the electron-nuclear hyperfine coupling tensors for each system and giving confidence that they represent a set of unique parameters that adequately describe the electronic structure of **1-X**. Furthermore, the model to the VTVH Mössbauer spectra for **1-F** allowed for its E/D parameter to be determined, and was found to be consistent with those modeled from the magnetometry and X-band EPR data.

Standard Spectroscopic Methods. NIR absorption spectra were measured at room temperature in CH_2Cl_2 solution (1.0 mM concentrations) with an Agilent Cary Series UV-vis-NIR spectrophotometer. Deconvolutions of the NIR absorption spectra were conducted by fitting Gaussian peaks to the observed low-energy features. The Gaussian expression for modeling the peaks is given by:

$$\varepsilon = b_0 + \frac{A}{\Gamma\sqrt{\frac{\pi}{4\ln 2}}} \cdot e^{\frac{-4\ln 2(E-E_\mathrm{c})^2}{\Gamma^2}}$$

where ε is the molar absorptivity (in units of M^{-1} cm^{-1}), b_0 is the baseline offset, $A = \int_\mathrm{Band} \varepsilon(\tilde{\nu})d\tilde{\nu}$ is the area under the Gaussian peak (in units of M^{-1} cm^{-2}), Γ is the linewidth (full width at half maximum, in units of cm^{-1}), E is energy (units of cm^{-1}), and E_c is the energy of the transition (the center of the Gaussian function). A table of the deconvoluted peaks is given in Table S22, with the peaks 1 and 2 being defined by the transitions in Scheme 2. Oscillator strengths for each transition were obtained from the areas of the Gaussian fit bands, and are given by:

$$f_\mathrm{osc} = \frac{4m_\mathrm{e}c\epsilon_0}{N_\mathrm{A}e^2}B_{12}$$

$$B_{12} = 1000 \cdot c \cdot \ln(10)\int_\mathrm{Band} \varepsilon(\tilde{\nu})d\tilde{\nu}$$

$$f_{\mathrm{osc}} = 1000 \cdot \frac{4m_{\mathrm{e}}c^2\epsilon_0}{N_{\mathrm{A}}e^2} \cdot \ln(10) \int_{\mathrm{Band}} \varepsilon(\tilde{\nu})d\tilde{\nu} = 4.319 \cdot 10^{-9} \int_{\mathrm{Band}} \varepsilon(\tilde{\nu})d\tilde{\nu}$$

where B_{12} is the Einstein photon absorption coefficient, $m_{\mathrm{e}}c^2$ = 4121484489 cm^{-1} is the invariant mass of an electron (c = 299792458 m s^{-1} is the speed of light in a vacuum), ϵ_0/e^2 = 68.51800012 cm^{-2} is the vacuum permittivity constant (e = 1.602176634 • 10^{-19} C is the elementary charge), N_{A} = 6.02214076 • 10^{23} mol^{-1} is Avogadro's constant, $\int_{\mathrm{Band}} \varepsilon(\tilde{\nu})d\tilde{\nu}$ is the area under a given Gaussian band (in units of L mol^{-1} cm^{-2}), and the factor of 1000 cm^3 L^{-1} makes f_{osc} dimensionless.

^{1}H NMR spectra were collected at room temperature using a Varian 400 MHz NMR spectrometer (except for **1-F**, whose NMR data was collected using a Varian 600 MHz NMR spectrometer instead) in $CDCl_3$ using the "s2pul" pulse sequence. All spectra were collected with 512 scans (except for **1-Br**, where 3328 scans were used) over a bandwidth of 240 ppm (except for **1-F**, which was collected over a bandwidth of 245 ppm) centered at 25 ppm, with an acquisition time of 0.0852 seconds (except for **1-F**, which was collected with an acquisition time of 0.1114 seconds) and relaxation delay of 0.01 seconds. All free induction decays (FIDs) were processed using an 11.7371 Hz (1/0.0852 s) exponential apodization (first point 0.50), except for the **1-F** complex, where instead an 8.9767 Hz exponential apodization (first point 0.50) was used. Forward linear prediction filling of the FIDs was conducted out to 131072 datapoints (262144 datapoints for **1-F**) because the original FID size was 8192 points (16384 points for **1-F**), i.e. four times. The Zhu-Bax linear prediction algorithm was used with 15 coefficients and 8176 basis points (16368 basis points for **1-F**). The Fourier-transformed (FT) spectra were baseline corrected via multipoint baseline correction with cubic splines. Peaks were appropriately phase-corrected, and the spectra were referenced to 7.26 ppm ($CHCl_3$ residual solvent peak). All integrals were normalized to the 27 proton singlet corresponding to the *t*Bu group protons ($–C(CH_3)_3$) in the $(Tp^{tBu,Me})^-$ ligand for each spectrum.

Perpendicular detection mode X-band EPR spectra were recorded on a Bruker ESP 300 spectrometer equipped with an Oxford Instruments ESR 900 liquid helium flow cryostat. Frozen solution samples of **1-F** were prepared at ~ 1 mM in toluene:dichloromethane (1:1 v/v) and collected at 4 K using a microwave frequency of 9.375 GHz, microwave power of 0.2 mW, field modulation of 100 KHz with 1 mT amplitude, time constant 320 ms, and averaged over two 90 s scans.

Powder XRD data for **1-F** were measured using a Rigaku Miniflex II diffractometer with a Cu K_α radiation source. Three scans were collected and averaged, with the baseline correction performed using Origin. Simulated PXRD data were obtained using Mercury.

Ligand-Field Theory Methods. Electronic structure analysis was performed using classical ligand-field theory (LFT) with the full d^6 basis set and the angular overlap model (AOM).[17, 18] NIR absorption bands were fitted using this model by the in-house software DDNFIT (written by Joshua Telser and available in the open-access SI). Analysis of the *ab initio* ligand field theory (AILFT) matrices were conducted using a MATLAB script, TpFeX_Fit_AOM_Script.m (written by Mihail Atanasov and edited by Daniel J. SantaLucia; available in the open-access SI).

The deconvoluted NIR absorption spectra band energies were used in the classical LFT fitting and are reported in Table S23. Fits using the AOM[17, 18] were conducted assuming that the most intense main bands observed in the spectra for the **1-X** series correspond to the $^5E(E) \rightarrow {}^5E(T_2)$ transition while the shoulders correspond to the $^5E(E) \rightarrow {}^5A_1(T_2)$ transition (implying that the compounds have idealized C_{3v} symmetry, Scheme 2). The experimental average X–Fe–N_{Tp} angles from SC-XRD for θ (see Tables S3-S5) and the ideal ϕ values (0, 120, 240°) were used along with two AOM bonding parameters (ε_σ(X) and ε_σ(N), thus assuming all pyrazolyl N-atom donors are identical), affording fits to the two observed bands for all four **1-X** complexes. The resulting parameters are given in Table S26 (first C_{3v} symmetry model).

Although the σ-bonding only models (i.e. only using ε_σ(X) and ε_σ(N) parameters) led to perfect fits of the experimental data, the models are inherently flawed due to their exclusion of π-bonding effects from both the halide and the pyrazolyl N-atom donors. While a π-bonding ε_π(X) parameter for the halide ligands could be added to the LFT models, the use of three AOM parameters (ε_σ(X), ε_π(X), and ε_σ(N)) led to overparameterization for fitting only two bands in each spectrum; it was therefore necessary to keep the ε_σ(N) parameters for the pyrazolyl N-atom donors fixed at constant values when fitting with the halide π-bonding model (second C_{3v} symmetry model in Table S26). Inclusion of an ε_π(N) parameter for the pyrazolyl N-atom donors was neglected because the π-bonding is unlikely to be cylindrical and the use of four parameters in the model would lead to meaningless results. (We stress that all of the AOM models here are underdetermined, and that the obtained AOM parameters should be taken with caution). The AOM parameters obtained from fitting the NIR absorption spectra capture physically-relevant trends across the **1-X** series. With the exception of **1-F**, the ε_σ(N) values are relatively similar to one another; if ε_σ(N) is fixed in the

halide π-bonding models for **1-Cl**, **1-Br**, and **1-I** at the average of the ε_σ(N) values obtained from the σ-bonding only model (4216 cm^{-1}), then no fits adequately model the data, as ε_π(X) converges to zero (except for **1-Cl** since the resultant ε_σ(N) value is sufficiently smaller than in the σ-only model). If ε_σ(N) is fixed at 4000 cm^{-1}, then the fit for **1-Br** models the NIR absorption data well, while fixing the ε_σ(N) parameter at 4000 cm^{-1} leads to physically less-reasonable models for the **1-Cl** and **1-I** data with ε_π(Cl) = 1583 cm^{-1} and ε_π(I) = 26.20 cm^{-1}, respectively. Therefore, the ε_σ(N) values were varied slightly for the models of **1-Cl** and **1-I**, with ε_σ(N) = 4100 cm^{-1} and ε_σ(N) = 3900 cm^{-1}, respectively. For **1-F**, fixing ε_σ(N) = 4000 cm^{-1} led to ε_σ(F) = 7434 cm^{-1} and ε_π(F) = 4577 cm^{-1}; this value for ε_π(F) is too large and thus unphysical, so it was necessary to instead fit the data fixing ε_σ(N) = 5000 cm^{-1} to obtain a physically reasonable value for ε_π(F). Thus, there is likely π-donation from the halide ligands, but it cannot be easily quantified from absorption spectroscopy alone. The σ-bonding parameters are reasonable based on results for other scorpionate complexes of general type $Tp^{R,R'}M(X,L)$.

Another assignment is possible based on the results of *ab initio* ligand field theory (AILFT). The above assignment of $^5E(E) \rightarrow {}^5A_1(T_2)$ cannot be used, and instead the observed bands are assigned only to the $^5E(E) \rightarrow {}^5E(T_2)$ transition, with the observed splitting of this transition into two bands due to the actual molecular geometries descending from C_{3v} to C_s symmetry (Scheme 2). This assignment is consistent with the vibronic coupling analysis and calculated spin-orbit corrected NIR absorption spectra from the CASSCF/NEVPT2 results (Figures S21-S22). If only C_s symmetry is assumed, then the ground $^5E(E)$ and excited $^5E(T_2)$ states each split into $^5A'$ and $^5A''$ states (Scheme 2, the relative order of each is irrelevant since all transitions in C_s symmetry are electric dipole allowed, whether in the *x*-,*y*-, or *z*-directions). The $^5A_1(T_2)$ excited state maps to $^5A'(A_1,T_2)$ (Scheme 2) and, importantly, is predicted to be very low in energy based on the CASSCF/NEVPT2 calculations (usually < 4000 cm^{-1}), suggesting that the $^5A'(E,E)/^5A''(E,E) \rightarrow {}^5A'(A_1,T_2)$ transition is not observed experimentally. In order to remove the degeneracies of the ^5E states, individual θ values (both from the Full Opt and H-atom only DFT optimized geometries) were used rather than average θ values (the ideal ϕ values were retained). The resulting fits, in contrast to the above classical LFT models, do *not* yield perfect fits to the spectra, but do resemble the AILFT values, as shown in Tables S25 and S26. Comparing the LFT and AILFT fits suggests that the splittings in the observed NIR bands are much larger than what would be expected from

deviation from C_{3v} symmetry due to packing effects alone. This discrepancy is a manifestation of the Jahn-Teller distortion energy, which cannot be reproduced via classical LFT arguments, consistent with the vibronic coupling analysis given in the main text.

Supplementary Equations for the JT-Analysis Including Quadratic Vibronic Coupling. Including quadratic vibronic coupling is essential for systems with large barriers, δ, between energetic minima and saddle points on a sombrero PES. This approach is outlined in more detail by Bersuker.[19] To assess the possibility of quadratic vibronic coupling in the **1-X** series, we used the **1-F** example since the DFT Full Opt (–) and (+) q_{bend} geometries may be considered instances of a minimum and saddle point, respectively. Equation S1 defines this barrier, δ, as:

$$\delta = \frac{4 \cdot \Delta \bar{E}_{\text{JT}} |G_{\text{bend}}|}{\bar{K}_{\text{bend}} + 2 \cdot |G_{\text{bend}}|} \quad \text{(S1)}$$

where $\bar{E}_{\text{JT}}$ (units of cm^{-1}) is the average of the Jahn-Teller stabilization energies of the (–) minimum and the (+) saddle point, $\bar{K}_{\text{bend}}$ (units of cm^{-1} Å^{-2}) is the average harmonic restoring force constant of the (–) minimum and the (+) saddle point, and G_{bend} (units of cm^{-1} Å^{-2}) is the quadratic vibronic coupling constant that leads to the barrier, δ (units of cm^{-1}). Equation S2 solves for G_{bend}:

$$|G_{\text{bend}}| = \frac{\delta \cdot \bar{K}_{\text{bend}}}{4 \cdot \Delta \bar{E}_{\text{JT}} - 2 \cdot \delta} \quad \text{(S2)}$$

For **1-F**: $\Delta \bar{E}_{\text{JT}} = 250.2$ cm^{-1}, $\bar{K}_{\text{bend}} = 3322.5$ cm^{-1} Å^{-2}, and $\delta = 11.00$ cm^{-1} (see Table 4 in the main text). Thus, $|G_{\text{bend}}| = 37.34$ cm^{-1} Å^{-2}. This extremely weak quadratic vibronic coupling and the small barrier between the minimum and saddle point suggest that the sombrero potentials are sufficient for describing the **1-X** series, and that quadratic vibronic coupling may be neglected.

Computational Methods. All quantum chemical theory (QCT) calculations were done using the ORCA quantum chemistry software package (versions 5.0.2, 5.0.3, 5.0.4, and an in-house developer version of 5.0) developed by Dr. F. Neese and coworkers (MPI-KoFo).[20, 21] All calculations used the default numerical integration grid setting (DEFGRID2).[22] Geometry optimizations and single point calculations were conducted at the density functional theory (DFT) level and utilized atom-pairwise dispersion correction with Becke-Johnson damping.[23, 24] Geometries used for calculations were derived from the crystallographic structures. Two separate sets of geometry optimizations were carried out to tight convergence criteria, one where only the positions of the hydrogen atoms were geometry optimized, and the other where all atom positions were geometry optimized.[22] A notable exception is the full optimization for the (–) isomer of **1-F**,

which instead was located with the default convergence criteria. The geometry optimizations utilized Becke's 1988 functional for exchange and Perdew's 1986 functional for correlation (BP86).[25, 26] Geometry optimizations utilized the resolution of identity (RI) approximation[27, 28] as well as the valence triple-ζ Karlsruhe basis set with polarization functions on main-group and transition-metal elements, def2-TZVP,[29] on all atoms as well as the def2/J Coulomb auxiliary basis set.[30] For the set of geometry optimizations where all atoms positions were geometry optimized, numerical frequency calculations were done to verify that no negative frequencies were present and that the geometries were energetic minima. DFT Single point calculations employed Becke's three-parameter hybrid functional for exchange and the Lee-Yang-Parr correlation functional (B3LYP).[31, 32] Tables of *xyz* coordinates for the optimized geometries are provided in Tables S28-S40.

Single point and CASSCF/NEVPT2 calculations accounted for scalar relativistic effects using the second-order Douglas-Kroll-Hess (DKH2) treatment.[33-37] The valence triple-ζ Karlsruhe basis set recontracted for DKH with polarization functions on main-group and transition-metal elements (DKH-def2-TZVP) was used on all atoms.[29] However, for the iodine atom in **1-I**, the SARC-DKH-TZVP basis set instead was used.[38-42] In addition to the RI approximation, the chain of sphere approximation was also used (RIJCOSX),[43] as was the SARC/J Coulomb[30, 38-41] and def2-TZVP/C exchange auxiliary basis sets.[44, 45] The quasi-restricted orbitals obtained from the single point DFT calculations were used as initial guesses for the first step of state-averaged complete active space self-consistent field (CASSCF) calculations, whereas the orbitals generated from the first step of the CASSCF calculations were used as initial guesses for the second step. (Note that in the first step of the CASSCF calculations, the basis sets DKH-def2-TZVP and def2-TZVP/C were replaced with DKH-def2-SVP and def2-SVP/C, respectively; the exception was the iodine atom of **1-I**, where SARC-DKH-TZVP was still used). The CAS(6,5) active space was comprised of six electrons in the five Fe 3*d*-orbitals; five quintet and 45 triplet states of the d^6 configuration were calculated. In the second step of the CASSCF calculations, dynamic electron correlation was accounted for by using strongly contracted *N*-electron valence state second-order perturbation theory (SC-NEVPT2).[46] Spin-Hamiltonian parameters, namely the *g*-values and ZFS parameters, were computed using the multiconfigurational CASSCF wavefunctions and their corresponding energies adjusted with NEVPT2. The effective Hamiltonian approach was used to calculate the *g*-

values, D, and E parameters for all complexes.[47] *Ab initio* ligand field theory (AILFT)[48, 49] was used to compute energies for the Fe 3*d*-orbitals.

X-ray Crystallographic Data Tables:

Table S1. Summary of X-ray Crystallographic Data Collection and Structure Refinement for **1-F** and **1-Cl**.[a]

	$[FeF(Tp^{tBu,Me})]$ (**1-F**)		$[FeCl(Tp^{tBu,Me})]$ (**1-Cl**)		
	**This Work**	**INUWAQ**	**This Work**	**INUTUH**	**INUTUH01**
Empirical Formula	$C_{24}H_{40}BN_6FFe$	$C_{24}H_{40}BN_6FFe$	$C_{24}H_{40}BN_6ClFe$	$C_{24}H_{40}BN_6ClFe$	$C_{24}H_{40}BN_6ClFe$
Formula Weight (g mol^{-1})	498.279	498.279	514.731	514.731	514.731
Crystal System	monoclinic	monoclinic	trigonal	trigonal	orthorhombic
Space Group	$P2_1/n$	$P2_1/n$	*R*3*m*	*R*3*m*	*Fdd*2
a (Å)	9.4292(3)	9.4515(12)	15.9131(5)	15.898(2)	32.512(8)
b (Å)	30.3263(8)	30.304(7)	15.9131(5)	15.898(2)	33.348(6)
c (Å)	9.4931(3)	9.5089(14)	9.4331(9)	9.422(3)	20.580(4)
α (°)	90	90	90	90	90
β (°)	101.040(3)	100.745(13)	90	90	90
γ (°)	90	90	120	120	90
Volume (Å^3)	2664.33(14)	2675.8(8)	2068.69(17)	2062.3(7)	22313(8)
Z	4	4	3	3	32
ρ_{calc} (g cm^{-3})	1.242	1.237	1.240	1.243	1.226
λ (Å)	1.54184	0.71073	0.71073	0.71073	0.71073
T (K)	100.4(8)	120(2)	100(2)	120(2)	120(2)
μ (mm^{-1})	4.768	0.593	0.666	0.668	0.659
F(000)	1064	1064	822	822	8768
2θ Range (°)	4.966-70.538	2.28-28.35	3.662-29.615	2.56-28.17	1.75-28.34
Reflections Collected	23422	22939	4407	3947	31031
Independent Reflections	5069	6327	1216	1141	11845
R_{int}	0.0917	0.0704	0.0237	0.0482	0.0668
Data / Restraints / Parameters	5069 / 0 / 310	6327 / 30 / 323	1216 / 1 / 69	1141 / 43 / 81	11845 / 1 / 625
Goodness-of-Fit on F^2	1.030	1.012	1.079	1.086	1.014
R_1 / wR_2 Indices [I >2σ(I)]	0.0551 / 0.1242	0.0510 / 0.1129	0.0238 / 0.0578	0.0408 / 0.0890	0.0555 / 0.1140
R_1 / wR_2 Indices [all data]	0.0827 / 0.1410	0.0939 / 0.1271	0.0244 / 0.0582	0.0466 / 0.0913	0.0841 / 0.1277
Largest Diff. Peak, Hole (e Å^{-3})	1.7, –0.77	0.47, –0.23	0.20, –0.20	0.54, –0.30	0.93, –0.60

[a] Previously-reported X-ray structures were published Theopold et al.[1]

Table S2. Summary of X-ray Crystallographic Data Collection and Structure Refinement for **1-Br** and **1-I**.[a]

	[FeBr(Tp^{tBu,Me})] **(1-Br)**		[FeI(Tp^{tBu,Me})] **(1-I)**		
	**This Work**	**INUVET**	**This Work**	**INUVIX**	**INUVUJ**
Empirical Formula	$C_{24}H_{40}BN_6BrFe$	$C_{24}H_{40}BN_6BrFe$	$C_{24}H_{40}BN_6IFe$	$C_{24}H_{40}BN_6IFe$	$C_{24}H_{40}BN_6IFe$
Formula Weight (g mol^{-1})	559.185	559.185	606.181	606.181	606.181
Crystal System	orthorhombic	orthorhombic	orthorhombic	monoclinic	monoclinic
Space Group	$Pca2_1$	$Pca2_1$	$Pca2_1$	$P2_1/c$	$P2_1/n$
a (Å)	18.82817(19)	18.804(3)	19.0102(4)	16.039(5)	10.215(6)
b (Å)	9.78756(9)	9.7659(16)	9.8779(3)	18.649(5)	19.386(11)
c (Å)	29.5953(3)	29.612(5)	29.6941(7)	20.174(6)	15.891(9)
α (°)	90	90	90	90	90
β (°)	90	90	90	112.710(6)	90.936(9)
γ (°)	90	90	90	90	90
Volume (Å^3)	5453.87(9)	5438.0(15)	5575.9(2)	5566(3)	3147(3)
Z	8	8	8	8	4
ρ_{calc} (g cm^{-3})	1.362	1.366	1.444	1.447	1.415
λ (Å)	1.54184	0.71073	0.71073	0.71073	0.71073
T (K)	100.6(3)	120(2)	99.9(3)	120(2)	120(2)
μ (mm^{-1})	6.317	2.047	1.671	1.674	1.488
F(000)	2336	2336	2480	2480	1376
2θ Range (°)	4.518-70.568	2.09-28.31	3.433-29.667	1.76-28.33	2.10-28.28
Reflections Collected	26387	57761	33298	36300	29718
Independent Reflections	9079	12644	11744	12714	7209
R_{int}	0.0482	0.0491	0.0363	0.103	0.0492
Data / Restraints / Parameters	9079 / 1 / 620	12644 / 1 / 625	11744 / 1 / 620	12714 / 0 / 625	7209 / 0 / 359
Goodness-of-Fit on F^2	1.075	1.039	1.062	1.052	1.078
R_1 / wR_2 Indices [I >2σ(I)]	0.0413 / 0.1072	0.0533 / 0.1444	0.0376 / 0.0850	0.0689 / 0.1349	0.0578 / 0.1497
R_1 / wR_2 Indices [all data]	0.0420 / 0.1081	0.0623 / 0.1506	0.0438 / 0.0898	0.1415 / 0.1685	0.0735 / 0.1591
Largest Diff. Peak, Hole (e Å^{-3})	2.6, –0.40	1.2, –1.5	1.3, –0.63	1.2, –0.83	2.4, –0.84

[a] Previously-reported X-ray structures were published by Theopold et al.[1]

Table S3. Selected bond lengths (Å) and bond angles (°) for **1-F** and **1-Cl**.

	**1-F**		**1-Cl**			
	**This Work**	**INUWAQ** [b]	**This Work**	**INUTUH** [b]	**INUTUH01** [a,b] **A**	**INUTUH01** [a,b] **B**
Fe1–X1	1.814(2)	1.8595(15)	2.2330(12)	2.2271(17)	2.2275(11)	2.2282(11)
Fe1–N_{Tp}	2.079(3)<br>2.113(3)<br>2.108(3)	2.0600(19)<br>2.092(2)<br>2.096(2)	2.075(2)	2.073(3)	2.104(3)<br>2.065(3)<br>2.097(3)	2.092(3)<br>2.084(3)<br>2.060(3)
X1–Fe1–N_{Tp}	132.42(12)<br>118.35(10)<br>122.92(12)	130.87(8)<br>123.68(8)<br>118.27(7)	123.65(6)	123.79(9)	120.44(10)<br>126.53(9)<br>122.38(9)	118.37(10)<br>120.71(10)<br>129.72(9)
N_{Tp} –Fe1–N_{Tp}	90.93(11)<br>90.03(10)<br>91.40(11)	91.47(8)<br>90.51(7)<br>91.72(8)	92.26(9)	92.07(12)	94.21(11)<br>92.92(12)<br>91.86(12)	96.37(11)<br>92.07(13)<br>91.38(12)

[a] Unit cell consists of two symmetry-independent, yet chemically equivalent, molecules. [b] X-ray structure reported by Theopold et al.[1]

Table S4. Selected bond lengths (Å) and bond angles (°) for **1-Br**.

	**1-Br (This Work)** [a]		**INUVET** [a,b]	
	**A**	**B**	**A**	**B**
Fe1–Br1	2.3904(9)	2.3891(9)	2.3672(8)	2.3535(9)
Fe1–N_{Tp}	2.058(5)<br>2.098(5)<br>2.110(5)	2.060(5)<br>2.088(5)<br>2.119(5)	2.091(4)<br>2.058(4)<br>2.110(4)	2.093(4)<br>2.066(4)<br>2.119(4)
Br1–Fe1–N_{Tp}	130.57(13)<br>120.04(12)<br>119.17(12)	128.43(13)<br>122.75(13)<br>117.85(12)	120.38(10)<br>130.80(11)<br>118.99(10)	122.59(10)<br>128.99(11)<br>117.72(10)
N_{Tp} –Fe1–N_{Tp}	89.03(18)<br>92.47(18)<br>97.13(17)	90.03(18)<br>94.07(17)<br>95.41(18)	97.13(14)<br>88.84(15)<br>92.13(14)	90.02(14)<br>95.16(14)<br>93.90(14)

[a] Unit cell consists of two symmetry-independent, yet chemically equivalent, molecules. [b] X-ray structure reported by Theopold et al.[1]

Table S5. Selected bond lengths (Å) and bond angles (°) for **1-I**.

	**1-I (This Work)** [a]		**INUVIX** [a,b]		
	**A**	**B**	**A**	**B**	**INUVUJ** [b]
Fe1–I1	2.5954(8)	2.5989(8)	2.5944(12)	2.5983(11)	2.6330(12)
$Fe1–N_{Tp}$	2.063(5)	2.056(5)	2.090(5)	2.099(5)	2.107(4)
	2.087(5)	2.087(5)	2.123(5)	2.095(5)	2.091(3)
	2.114(5)	2.122(5)	2.065(5)	2.071(5)	2.090(4)
$I1–Fe1–N_{Tp}$	128.51(14)	128.86(14)	120.12(15)	119.39(15)	120.38(10)
	121.53(13)	120.32(13)	119.89(15)	119.48(15)	125.08(9)
	117.79(14)	119.42(14)	128.74(16)	129.44(15)	123.36(9)
N_{Tp} –Fe1–N_{Tp}	89.4(2)	88.9(2)	88.9(2)	99.7(2)	95.68(13)
	94.5(2)	92.45(19)	99.4(2)	90.0(2)	92.44(13)
	97.36(19)	98.98(19)	91.9(2)	91.1(2)	91.64(13)

[a] Unit cell consists of two symmetry-independent, yet chemically equivalent, molecules. [b] X-ray structure reported by Theopold et al.[1]

Magnetometry Fit Table:

Table S6. Summary of the spin-Hamiltonian fitting parameters derived by modeling simultaneously the paramagnetic susceptibility and VTVH data for each **1-X** complex.[a]

Parameter	1-F	1-Cl	1-Br	1-I
g_x	2.028(4)	*2.002*	2.031(4)	2.010(4)
g_y	*2.028*	$g_{y/z}$ = 2.235(4)	2.386(5)	2.381(5)
g_z	2.394(5)	$g_{z/y}$ = *2.002*	2.016(4)	2.306(5)
D (cm^{-1})	–20.8(2)	±11.39(11) [d]	+8.28(8)	–12.29(12)
E/D	0.000-0.105 [c]	*0.333*	0.255(4)	0.265(4)
TIP (cm^3 mol^{-1})	0.000238(12)	0.000169(8)	0.000101(5)	0.000360(18)
Diamagnetic Impurity (mol %) [b]	13.8(7)	------	8.6(4)	15.7(8)

[a] Italicized values are parameters that were fixed, and hence are reported with no uncertainties.

[b] The diamagnetic impurities result from error in determining the contribution from the sample holder, *n*-eicosane fixative, and/or (in the case of **1-F**) the presence of Tl($Tp^{tBu,Me}$).

[c] The error plots for **1-F** (Figure S2) show that rhombicity values between the range of 0.000-0.105 model the data well; the fitted value was 0.071(14), where the much higher error (~20%) in the rhombicity reflects the relative insensitivity of the fit to rhombicity values in this range.

[d] The sign of D does not affect the model at the rhombic limit; the values for g_y and g_z are swapped with each other depending on the sign of the D parameter used in the model.

HFEPR/FIRMS Data and Fit Tables:

Table S7. Table of experimentally-observed transitions at zero applied field in the FIRMS spectra for each complex. All energies are in cm^{-1}.

1-F	1-Cl	1-Br	1-I
62.53	22.42	21.79	32.39
65.18	31.10	23.57	36.52
70.45	32.91	25.86	38.03
79.58	47.55	45.80	51.59
	51.23	47.31	56.29
	54.00	50.32	60.63

Table S8. Table of predicted energies (cm^{-1}) for all possible ΔM_S transitions derived from the spin Hamiltonian parameters reported in Table S9.

Transition	1-F	1-Cl	1-Br	1-I
$\Delta_1 = -D - 3E + 2\sqrt{D^2 + 3E^2}$	63.5	4.21	4.37	55.2
$\Delta_2 = -D + 3E + 2\sqrt{D^2 + 3E^2}$	55.8	21.7	21.6	37.2
$\Delta_3 = 2D + 2\sqrt{D^2 + 3E^2}$	0.254	45.1	45.3	1.77
$\Delta_4 = 4\sqrt{D^2 + 3E^2}$	79.7	47.3	47.5	62.7
$\lvert\Delta_5\rvert = \lvert 6E\rvert$	7.78	17.5	17.2	18.0
$\lvert\Delta_6\rvert = \lvert 3D + 3E\rvert$	63.3	40.9	41.0	53.4
$\Delta_7 = 2\sqrt{D^2 + 3E^2} + D + 3E$	16.2	43.1	43.2	7.57
$\lvert\Delta_8\rvert = \lvert 3D - 3E\rvert$	55.5	23.3	23.8	35.4
$\Delta_9 = 2\sqrt{D^2 + 3E^2} + D - 3E$	23.9	25.6	26.0	25.6
$\Delta_{10} = 2\sqrt{D^2 + 3E^2} - 2D$	79.5	2.27	2.17	61.0

Table S9. The spin-Hamiltonian parameters derived from simulating the HFEPR and FIRMS data for each **1-X** complex.[a]

Parameter	1-F [b]	1-Cl	1-Br	1-I
g_x	*2.028*	2.159(4)	2.238(4)	*2.010*
g_y	*2.028*	2.248(15)	2.257(8)	*2.381*
g_z	*2.394*	2.337(18)	2.22(2)	*2.306*
D (cm^{-1})	–19.8(4)	+12.12(3)	+10.741(18)	–14.8(9)
E/D	0.0655(2)	0.2406(7)	0.2669(6)	0.20(2) [c]
B_4^0 (cm^{-1})	---	–0.0016(3)	–0.0013(3)	---

[a] The fourth-order zero-field splitting term B_4^3 was ignored; attempts to include it did not improve the resulting fits. The signs of the D values were taken from simulations of the magnetometry and Mössbauer data. Italicized values are parameters that were fixed, and hence are reported with no uncertainties.

[b] The g-values and D parameter obtained from the fit to the magnetometry data were used as fixed parameters in a spin-Hamiltonian fit for the X-band EPR spectrum of **1-F** (Figure S6), affording a reliable value for the E/D ratio. This E/D ratio was then fixed and used to obtain a D parameter consistent with the FIRMS data.

[c] The magnitude of the D parameter for **1-I** is apparently larger and the E/D parameter is therefore smaller than obtained by other methods due to the difficulty of simulating accurate spin-Hamiltonian parameters based on the FIRMS data alone. The relatively large uncertainties reflect the lack of precision.

Mössbauer Fit Tables:

Table S10. Summary of the spin-Hamiltonian fitting parameters derived by modeling the VTVH Mössbauer data for each **1-X** complex.[a]

Parameter	1-F (sub 1) [b]	1-F (sub 2) [b]	1-Cl	1-Br	1-I
δ (mm s^{-1})	*0.910*	*1.000*	*0.930*	*0.970*	*0.930*
ΔE_{Q} (mm s^{-1})	*+1.87*	*+1.36*	*–2.28*	*±2.75*	*±2.77*
η	*0.00*	*0.00*	**0.72(7)**	*1.00* [c]	*1.00* [c]
β (°)	**16(3)**	**16(3)**			
Γ (mm s^{-1})	*0.31*	*0.34*	*0.40*	*0.35*	*0.33*
g_x	*2.028*	*2.028*	*2.159*	*2.238*	*2.010*
g_y	*2.028*	*2.028*	*2.248*	*2.257*	*2.381*
g_z	*2.394*	*2.394*	*2.337*	*2.22*	*2.306*
D (cm^{-1})	*–20.8*	*–20.8*	*+12.12*	*+10.741*	*–12.29*
E/D	**0.10(2)**	**0.11(2)**	*0.2406*	*0.2669*	*0.265*
$\frac{A_{xx}}{g_{\mathrm{N}}\mu_{\mathrm{N}}}$ (T)	*–21.0*	*–21.0*	**–6.1(9)**	**–6.8(1.0)**	**–24.8(1.2)**
$\frac{A_{yy}}{g_{\mathrm{N}}\mu_{\mathrm{N}}}$ (T)	*–21.0*	*–21.0*	**+4.6(7)**	**+1.8(3)**	**–26.4(1.3)**
$\frac{A_{zz}}{g_{\mathrm{N}}\mu_{\mathrm{N}}}$ (T)	**+12.9(2)**	**+12.2(2)**	**–28.1(1.4)**	**–22.5(1.1)**	**+4.51(0.11)**

[a] Italicized values are parameters that were fixed, and hence are reported with no uncertainties. Bold values are those that were allowed to refine freely.

[b] An alternative fit to the data for **1-F** fixing the rhombicity parameter as $E/D = 0.071$ from the magnetometry results is included in Figure S17 and Table S11, showing a worse fit to the data.

[c] The sign of the quadrupole splitting is irrelevant at the limit of $\eta = 1$.

Table S11. Summary of the alternative spin-Hamiltonian fitting parameters derived by modeling the VTVH Mössbauer data for **1-F** where E/D was instead a fixed parameter (Figure S17). Italicized values are parameters that were fixed, and hence are reported with no uncertainties. Bold values are those that were allowed to refine freely.

Parameter	1-F (sub 1)	1-F (sub 2)
δ (mm s^{-1})	*0.910*	*1.000*
ΔE_Q (mm s^{-1})	*+1.87*	*+1.36*
η	*0.00*	*0.00*
β (°)	**16(3)**	**16(3)**
Γ (mm s^{-1})	*0.31*	*0.34*
g_x	*2.028*	*2.028*
g_y	*2.028*	*2.028*
g_z	*2.394*	*2.394*
D (cm^{-1})	*–20.8*	*–20.8*
E/D	*0.071*	*0.071*
$A_{xx}/g_N\mu_N$ (T)	*–21.0*	*–21.0*
$A_{yy}/g_N\mu_N$ (T)	*–21.0*	*–21.0*
$A_{zz}/g_N\mu_N$ (T)	**+12.8(2)**	**+12.0(2)**

Electronic Structure Calculations Tables:

Table S12. Summary of Fe–N_{Tp} bond distances (R, in Å) and X–Fe–N_{Tp} bond angles (θ, in °) from the H-only optimized and Full Opt (+) and Full Opt (–) geometries. Previously reported X-ray data for **1-Cl** (INUTUH01 reported by Theopold et al.[1]) were used for the H-only geometry instead of the X-ray data reported herein due to the crystallographically-imposed trigonal symmetry.

	**1-F**			**1-Cl**		**1-Br**		**1-I**	
	H-only	Full (–)	Full (+)	H-only INUTUH01 Mol. A(B)	Full (+)	H-only Mol. A(B)	Full (+)	H-only Mol. A(B)	Full (+)
R_{FeX}	1.814	1.827	1.827	2.228(2.228)	2.212	2.390(2.389)	2.367	2.599(2.595)	2.567
R_{FeN_1}	2.113	2.081	2.043	2.104(2.092)	2.049	2.110(2.119)	2.052	2.122(2.114)	2.059
R_{FeN_3}	2.108	2.065	2.042	2.097(2.084)	2.049	2.098(2.088)	2.051	2.087(2.087)	2.058
R_{FeN_5}	2.079	2.034	2.092	2.065(2.060)	2.091	2.058(2.060)	2.088	2.056(2.063)	2.091
$R_{FeN_{avg}}$	2.100	2.060	2.059	2.089(2.079)	2.063	2.089(2.089)	2.064	2.088(2.088)	2.069
θ_{XFeN_1}	118.4	117.4	127.9	120.4(118.4)	125.0	119.2(117.9)	124.1	119.4(117.8)	123.6
θ_{XFeN_3}	122.9	121.0	128.1	122.4(120.7)	125.7	120.0(122.8)	124.7	120.3(121.5)	124.0
θ_{XFeN_5}	132.4	133.2	115.4	126.5(130.0)	119.1	130.6(128.4)	120.0	128.9(128.5)	120.5
$\theta_{XFeN_{avg}}$	124.6	123.9	123.8	123.1(122.9)	123.3	123.3(123.0)	123.0	122.9(122.6)	122.7

[a] For the purpose of the "LFT using AILFT" model (Table S26), the θ_{XFeN} values used were averaged between molecules A and B from the H-only optimized geometries for **1-Br** and **1-I**. For **1-Br**, the values were: 118.6°, 121.4°, and 129.5°. For **1-I**, the values were: 118.6°, 120.9°, and 128.7°.

Table S13. Contribution of individual quintet and triplet roots to the $\overleftrightarrow{\boldsymbol{D}}$ tensor for **1-F** using the effective Hamiltonian approach based on the NEVPT2-corrected SA-CASSCF transition energies for the roots. All D and E values are in cm^{-1}.

		**1-F**					
		H-Only		Full (–)		Full (+)	
Mult	Root	*D*	*E*	*D*	*E*	*D*	*E*
5	1	–22.206	0.032	–15.972	0.089	–16.237	–0.279
5	2	0.961	–1.190	1.116	–1.288	0.255	–1.300
5	3	–1.450	0.548	–1.827	0.549	–1.835	–0.199
5	4	0.320	–0.162	0.291	–0.152	0.584	0.583
Sum	1-4	–22.375	–0.772	–16.392	–0.802	–17.233	–1.195
3	0	–0.190	–0.624	–0.151	–0.636	0.004	0.557
3	1	–0.398	0.436	–0.527	0.462	–0.630	–0.622
3	2	–0.001	–0.023	–0.022	–0.046	–0.065	–0.065
3	3	0.035	0.000	0.046	–0.002	0.116	0.018
3	4	–0.004	–0.007	–0.000	–0.001	–0.010	–0.010
3	5	–0.126	–0.194	–0.077	–0.078	–0.170	–0.169
3	6	–0.049	0.024	–0.107	–0.036	–0.006	0.059
3	7	–0.059	0.053	–0.049	0.043	–0.050	–0.049
3	8	0.151	–0.073	0.092	–0.083	–0.040	0.222
3	9	–0.019	0.011	–0.020	0.019	–0.020	–0.020
3	10	0.019	0.009	0.053	0.002	0.096	0.004
3	11	–0.209	–0.201	–0.220	–0.218	–0.158	–0.158
3	12	–0.090	0.074	–0.025	0.022	–0.093	0.093
3	13	–0.360	0.266	–0.072	0.058	–0.015	–0.015
3	14	–0.566	0.511	–0.152	0.045	–0.455	0.466
3	15	–0.376	–0.080	–0.814	0.644	–0.451	–0.451
3	16	0.076	0.010	0.001	–0.033	–0.203	0.317
3	17	–0.068	–0.045	–0.151	–0.028	–0.131	0.141
3	18	–0.088	–0.006	–0.142	0.057	–0.104	–0.104
3	19	–0.020	0.011	–0.007	–0.000	–0.010	0.017
3	20	–0.339	–0.388	–0.278	–0.414	–0.441	–0.441
3	21	0.793	–0.023	0.675	–0.062	0.847	0.001
3	22	–0.039	–0.041	0.011	–0.007	–0.009	–0.009
3	23	0.000	–0.000	0.000	0.001	–0.000	–0.000
3	24	–0.021	–0.011	–0.041	–0.038	–0.008	0.017

3	25	–0.016	0.004	–0.018	0.001	–0.034	–0.034
3	26	–0.057	–0.030	–0.048	0.046	–0.034	0.046
3	27	–0.045	0.037	–0.027	–0.039	–0.055	–0.055
3	28	–0.003	0.002	–0.001	0.001	–0.006	–0.009
3	29	0.013	–0.007	0.015	–0.005	0.019	–0.001
3	30	–0.098	0.109	–0.125	0.132	–0.131	0.147
3	31	0.118	–0.019	0.139	–0.029	0.184	0.001
3	32	0.000	–0.000	–0.000	0.001	–0.002	–0.002
3	33	–0.034	0.001	–0.018	0.003	–0.061	–0.061
3	34	0.374	–0.012	0.370	–0.017	0.407	0.029
3	35	–0.000	–0.000	–0.000	–0.000	–0.000	–0.000
3	36	0.001	–0.002	0.001	–0.002	–0.005	0.008
3	37	–0.007	0.005	–0.007	0.006	–0.003	–0.002
3	38	0.008	–0.001	0.005	–0.000	0.000	–0.000
3	39	0.016	–0.001	0.014	–0.002	0.024	0.000
3	40	–0.005	–0.005	–0.004	–0.005	–0.008	–0.008
3	41	0.000	0.000	–0.000	0.000	0.008	0.000
3	42	–0.010	–0.009	–0.015	–0.015	–0.023	–0.023
3	43	–0.062	0.008	–0.055	0.013	–0.052	–0.052
3	44	–0.037	–0.012	–0.033	–0.010	–0.041	0.063
Sum	0-44	–1.792	–0.243	–1.784	–0.250	–1.819	–0.154
Total Sum	All	–24.167	–1.015	–18.176	–1.052	–19.052	–1.349

Table S14. Contribution of individual quintet and triplet roots to the $\overleftrightarrow{\boldsymbol{D}}$ tensor for **1-Cl** using the effective Hamiltonian approach based on the NEVPT2-corrected SA-CASSCF transition energies for the roots. All D and E values are in cm^{-1}. H-only optimized geometries for previously reported X-ray data for **1-Cl** (INUTUH01 reported by Theopold et al.[1]) are also included.

		**1-Cl**							
		H-Only		INUTUH01 (Mol. A)		INUTUH01 (Mol. B)		Full (+)	
Mult	Root	*D*	*E*	*D*	*E*	*D*	*E*	*D*	*E*
5	1	–39.602	–0.027	–15.585	0.011	4.332	4.327	–13.970	–0.646
5	2	3.728	–3.739	3.514	–3.730	4.181	–3.607	2.638	–3.469
5	3	–2.405	–0.938	–3.768	0.593	1.583	2.385	–3.831	–0.374
5	4	0.847	0.847	0.634	–0.386	0.353	–0.419	0.742	0.717
Sum	1-4	–37.432	–3.857	–15.205	–3.512	10.449	2.686	–14.421	–3.772
3	0	0.471	0.422	0.114	–0.338	1.072	–0.171	–0.008	0.613
3	1	–0.455	–0.455	–0.450	0.334	–0.410	0.436	–0.429	–0.411
3	2	–0.142	–0.142	–0.124	–0.142	0.285	–0.010	–0.196	–0.197
3	3	–0.013	–0.013	0.145	–0.005	–0.044	–0.067	0.198	0.011
3	4	0.182	0.016	–0.009	0.007	–0.002	0.003	–0.008	–0.007
3	5	–0.105	0.105	–0.065	0.069	–0.060	0.052	–0.068	0.081
3	6	–0.255	–0.255	–0.238	–0.243	0.348	–0.020	–0.211	–0.211
3	7	–0.076	–0.076	–0.082	0.061	–0.077	0.083	–0.058	–0.055
3	8	–0.007	0.073	–0.063	–0.055	0.223	0.002	–0.108	0.137
3	9	–0.049	–0.049	–0.033	0.021	–0.013	0.015	–0.061	–0.059
3	10	0.134	0.044	0.059	–0.048	0.036	–0.046	0.132	0.009
3	11	–0.225	–0.225	–0.112	–0.138	–0.056	0.052	–0.031	–0.029
3	12	0.380	0.067	–0.110	–0.037	0.402	–0.022	–0.294	–0.293
3	13	–0.076	–0.077	0.185	–0.141	0.045	–0.071	0.199	–0.008
3	14	–0.231	0.232	–0.304	0.323	–0.220	0.245	–0.195	0.267
3	15	–0.183	0.256	–0.199	–0.154	–0.268	0.272	–0.308	0.333
3	16	–0.263	–0.263	–0.273	0.231	0.358	0.006	–0.224	–0.226
3	17	–0.232	0.233	–0.221	0.233	0.022	0.050	–0.265	0.288
3	18	–0.038	0.066	–0.015	–0.014	–0.091	0.166	0.009	–0.001
3	19	–0.063	–0.063	–0.181	–0.200	0.300	–0.021	–0.001	0.000
3	20	–0.222	–0.222	–0.048	0.003	0.013	0.008	–0.391	–0.391
3	21	–0.298	–0.298	–0.320	–0.327	0.577	–0.013	–0.206	–0.206
3	22	0.723	0.015	0.698	–0.016	–0.220	–0.337	0.690	0.042

3	23	–0.021	–0.021	–0.014	0.012	–0.030	0.026	–0.026	–0.025
3	24	0.002	0.005	0.000	0.001	0.009	0.001	–0.001	0.009
3	25	–0.005	–0.005	–0.005	0.005	–0.002	0.003	–0.005	–0.005
3	26	–0.014	0.014	–0.016	0.017	–0.016	0.014	–0.009	0.015
3	27	–0.018	–0.018	–0.029	–0.030	0.073	–0.002	–0.029	–0.029
3	28	–0.010	–0.010	–0.003	0.002	–0.000	0.000	–0.007	–0.007
3	29	–0.000	0.010	0.005	–0.005	0.002	–0.008	0.005	0.002
3	30	0.327	0.000	0.257	–0.003	–0.084	–0.100	0.298	0.000
3	31	0.000	–0.000	–0.158	0.161	–0.211	0.194	–0.209	0.209
3	32	–0.195	0.195	–0.038	0.039	–0.000	0.000	–0.000	0.000
3	33	0.349	0.113	0.491	–0.059	–0.224	–0.288	0.522	0.042
3	34	–0.101	–0.101	–0.075	0.053	–0.057	0.054	–0.085	–0.081
3	35	–0.002	–0.002	–0.002	–0.002	0.003	–0.000	–0.002	–0.001
3	36	–0.001	0.005	0.001	0.001	–0.001	–0.000	–0.005	0.007
3	37	–0.005	–0.005	–0.004	0.004	–0.003	0.002	–0.001	–0.001
3	38	–0.011	–0.011	–0.009	–0.010	0.012	–0.004	–0.008	–0.008
3	39	–0.000	–0.000	–0.003	–0.001	–0.001	0.005	–0.004	–0.004
3	40	0.034	0.000	0.026	–0.000	–0.012	–0.012	0.022	0.000
3	41	–0.014	0.014	–0.004	0.004	–0.001	0.001	0.003	0.004
3	42	–0.038	–0.038	–0.023	–0.023	0.006	0.003	–0.035	–0.034
3	43	–0.058	–0.058	–0.067	0.039	–0.041	0.055	–0.059	–0.055
3	44	0.030	0.054	–0.019	–0.047	0.167	–0.010	–0.024	0.071
Sum	0-44	–0.794	–0.468	–1.335	–0.418	1.809	0.546	–1.493	–0.204
Total Sum	All	–38.226	–4.325	–16.540	–3.930	12.258	3.232	–15.914	–3.976

Table S15. Contribution of individual quintet and triplet roots to the $\overleftrightarrow{\boldsymbol{D}}$ tensor for **1-Br** using the effective Hamiltonian approach based on the NEVPT2-corrected SA-CASSCF transition energies for the roots. All D and E values are in cm^{-1}.

**1-Br**							
		H-Only (Mol. A)		H-Only (Mol. B)		Full (+)	
Mult	Root	*D*	*E*	*D*	*E*	*D*	*E*
5	1	2.385	0.880	2.874	–1.549	6.794	3.090
5	2	5.314	1.171	5.531	2.847	5.652	–3.060
5	3	2.327	0.512	2.383	1.595	2.796	2.635
5	4	0.183	–0.021	–0.631	–0.068	–1.558	–0.005
Sum	1-4	10.209	2.542	10.157	2.825	13.684	2.660
3	0	0.879	0.289	–0.067	0.626	–0.950	0.641
3	1	–0.326	0.070	0.045	0.064	0.729	0.001
3	2	0.458	–0.051	0.531	–0.017	0.450	–0.001
3	3	–0.063	–0.016	–0.086	–0.051	–0.117	–0.117
3	4	–0.001	–0.000	0.001	0.003	0.014	0.000
3	5	–0.047	–0.017	–0.057	–0.027	–0.082	0.051
3	6	0.281	0.011	0.306	–0.000	0.391	–0.000
3	7	–0.063	–0.014	0.043	0.001	0.097	0.000
3	8	0.136	0.031	–0.026	0.065	–0.124	0.125
3	9	0.002	0.013	0.024	0.014	0.112	0.001
3	10	0.070	–0.015	0.047	–0.019	–0.055	–0.015
3	11	–0.067	0.008	0.024	0.001	0.112	0.001
3	12	0.095	0.015	–0.159	0.013	–0.208	–0.089
3	13	0.505	–0.098	0.800	–0.012	–0.340	0.332
3	14	–0.213	0.217	–0.328	0.305	0.674	0.000
3	15	–0.157	0.004	–0.140	0.033	–0.228	0.227
3	16	0.175	0.005	0.166	0.009	0.306	0.001
3	17	0.054	–0.036	–0.146	–0.099	–0.236	0.161
3	18	–0.247	–0.043	–0.049	–0.016	0.089	–0.009
3	19	0.187	0.007	0.171	0.036	0.004	–0.024
3	20	0.629	0.025	0.622	0.020	0.280	0.000
3	21	–0.013	–0.013	0.176	–0.011	0.731	–0.000
3	22	–0.077	0.152	–0.297	0.203	–0.408	–0.120
3	23	–0.030	–0.005	0.025	–0.008	0.085	0.000
3	24	0.009	0.004	–0.004	–0.002	–0.010	0.000
3	25	–0.001	–0.002	0.007	–0.002	0.001	0.000

3	26	–0.012	–0.009	–0.014	–0.012	–0.011	0.002
3	27	0.069	0.001	0.074	0.000	0.058	–0.000
3	28	–0.001	–0.000	0.001	0.000	0.007	0.000
3	29	–0.007	–0.000	–0.015	–0.000	–0.024	–0.013
3	30	–0.060	0.005	–0.099	0.005	–0.148	–0.133
3	31	–0.202	–0.015	–0.146	–0.001	0.000	0.000
3	32	–0.011	–0.001	–0.067	–0.005	–0.221	0.192
3	33	–0.286	–0.081	–0.294	–0.186	–0.340	–0.327
3	34	–0.024	0.013	0.060	0.008	0.165	0.000
3	35	0.002	0.000	0.000	0.000	0.002	0.000
3	36	–0.002	0.002	–0.003	0.002	–0.005	0.005
3	37	–0.001	–0.000	–0.001	–0.001	0.001	0.000
3	38	0.009	–0.004	0.016	–0.002	0.036	–0.000
3	39	–0.003	0.001	–0.001	–0.001	–0.005	0.005
3	40	–0.011	0.002	0.002	–0.000	0.002	–0.000
3	41	0.001	–0.000	–0.010	–0.002	–0.013	–0.013
3	42	–0.042	0.007	0.028	0.005	0.090	0.000
3	43	0.005	0.004	0.040	0.004	0.081	0.000
3	44	0.169	0.022	0.029	0.066	–0.107	0.079
Sum	0-44	1.768	0.488	1.229	1.009	0.885	0.963
Total Sum	All	11.977	3.030	11.386	3.834	14.569	3.623

Table S16. Contribution of individual quintet and triplet roots to the $\overleftrightarrow{\boldsymbol{D}}$ tensor for **1-I** using the effective Hamiltonian approach based on the NEVPT2-corrected SA-CASSCF transition energies for the roots. All D and E values are in cm^{-1}.

**1-I**							
		H-Only (Mol. A)		H-Only (Mol. B)		Full (+)	
Mult	Root	*D*	*E*	*D*	*E*	*D*	*E*
5	1	–15.566	–0.033	–14.542	–0.613	4.527	–4.145
5	2	1.037	–1.261	–0.366	–0.586	8.695	8.635
5	3	1.148	–2.536	–0.697	–1.828	3.076	–0.340
5	4	–0.494	–0.014	0.138	0.236	–1.639	0.005
Sum	1-4	–13.875	–3.844	–15.467	–2.791	14.659	4.155
3	0	–0.823	–0.435	–0.740	0.051	–0.953	0.407
3	1	0.159	0.005	0.092	0.079	0.602	–0.004
3	2	0.028	–0.223	–0.240	–0.338	0.482	0.001
3	3	–0.038	0.067	0.014	0.048	–0.113	0.065
3	4	–0.000	–0.000	0.000	–0.000	0.008	–0.000
3	5	0.040	–0.003	0.063	0.000	–0.065	–0.063
3	6	0.046	–0.015	–0.033	–0.059	0.376	0.000
3	7	–0.068	–0.066	–0.064	–0.076	0.048	–0.001
3	8	–0.028	–0.064	0.050	–0.017	–0.105	–0.067
3	9	–0.020	–0.001	–0.019	–0.000	0.054	–0.000
3	10	–0.025	–0.032	–0.007	–0.017	–0.008	0.009
3	11	0.100	0.026	–0.009	–0.007	0.286	–0.001
3	12	–0.115	–0.111	–0.172	–0.010	–0.322	0.322
3	13	–0.274	–0.061	–0.285	–0.131	–0.375	–0.043
3	14	0.010	–0.078	–0.079	0.043	0.624	0.000
3	15	0.101	–0.036	0.161	–0.003	–0.149	–0.107
3	16	–0.014	–0.016	–0.026	–0.029	0.203	–0.001
3	17	–0.073	0.010	–0.044	–0.042	0.116	0.000
3	18	–0.080	0.046	0.095	0.010	–0.174	–0.164
3	19	0.407	0.004	0.241	0.033	–0.104	–0.018
3	20	–0.428	–0.296	–0.454	–0.341	0.029	–0.000
3	21	–0.021	–0.025	0.001	–0.011	0.932	0.000
3	22	–0.438	0.108	–0.448	0.232	–0.395	0.376
3	23	0.102	–0.001	0.014	–0.022	0.119	–0.000
3	24	–0.000	–0.001	0.012	–0.000	–0.009	–0.009

3	25	0.008	–0.001	0.004	–0.001	0.000	–0.000
3	26	0.020	0.000	0.022	0.000	–0.007	–0.007
3	27	–0.041	–0.012	–0.043	–0.035	0.065	0.000
3	28	0.001	0.001	0.001	0.007	0.003	–0.000
3	29	–0.040	0.013	–0.022	0.027	–0.057	0.052
3	30	–0.062	0.054	–0.047	0.054	–0.131	0.102
3	31	0.158	0.011	0.056	0.007	0.000	–0.000
3	32	0.188	0.015	0.262	0.032	–0.227	–0.192
3	33	–0.093	0.241	0.083	0.190	–0.344	0.075
3	34	0.063	0.004	–0.010	–0.019	0.150	–0.000
3	35	–0.000	–0.001	0.001	–0.000	0.001	0.000
3	36	0.002	–0.000	0.002	0.000	–0.003	–0.003
3	37	0.001	–0.000	–0.000	–0.001	–0.000	–0.000
3	38	–0.011	0.003	–0.009	–0.005	0.045	–0.000
3	39	0.005	0.000	0.003	0.000	–0.004	–0.004
3	40	0.003	0.002	0.002	0.001	–0.008	0.005
3	41	–0.008	0.007	–0.002	0.004	0.000	0.000
3	42	0.051	0.007	0.001	–0.008	0.117	–0.001
3	43	–0.007	0.006	–0.010	0.017	–0.119	0.048
3	44	–0.118	–0.098	–0.105	–0.045	0.039	0.001
Sum	0-44	–1.332	–0.946	–1.688	–0.382	0.627	0.778
Total Sum	All	–15.207	–4.790	–17.155	–3.173	15.286	4.933

Table S17. Comparison of spin-Hamiltonian parameters obtained from CASSCF/NEVPT2 calculations of the **1-X** series with only spin-orbit coupling (SOC) contributions considered or with both SOC and spin-spin coupling (SSC) contributions considered.

Complex	Method	g_x	g_y	g_z	g_{avg} [b]	D (cm^{-1})	E/D
**1-F**	SOC (H-Only)	2.034	2.004	2.447	2.171	–23.19	0.049
	SOC+SSC (H-Only)	2.034	2.004	2.447	2.171	–23.51	0.051
	SOC (Full Opt (–))	2.015	2.047	2.346	2.141	–17.33	0.068
	SOC+SSC (Full Opt (–))	2.015	2.047	2.346	2.141	–17.64	0.070
	SOC (Full Opt (+))	2.053	2.006	2.360	2.145	–18.10	0.078
	SOC+SSC (Full Opt (+))	2.053	2.006	2.360	2.145	–18.42	0.079
**1-Cl**	SOC (H-Only)	1.746	1.725	2.585	2.058	–38.28	0.154
	SOC+SSC (H-Only)	1.745	1.726	2.585	2.058	–38.43	0.156
	SOC (H-only Mol. A) [a]	2.002	2.125	2.358	2.167	–15.48	0.301
	SOC+SSC (H-only Mol. A) [a]	2.002	2.125	2.358	2.167	–15.68	0.304
	SOC (H-only Mol. B) [a]	2.010	2.160	2.261	2.146	+11.95	0.217
	SOC+SSC (H-only Mol. B) [a]	2.010	2.160	2.262	2.147	+12.28	0.217
	SOC (Full Opt (+))	2.134	1.997	2.345	2.163	–14.74	0.318
	SOC+SSC (Full Opt (+))	2.134	1.997	2.345	2.163	–14.96	0.318
**1-Br**	SOC (H-Only Mol. A)	2.167	2.015	2.258	2.149	+11.42	0.239
	SOC+SSC (H-Only Mol. A)	2.167	2.015	2.258	2.149	+11.75	0.238
	SOC (H-Only Mol. B)	2.168	2.018	2.261	2.151	+11.27	0.287
	SOC+SSC (H-Only Mol. B)	2.168	2.018	2.262	2.151	+11.53	0.292
	SOC (Full Opt (+))	2.001	2.194	2.299	2.168	+15.86	0.134
	SOC+SSC (Full Opt (+))	2.001	2.193	2.299	2.168	+16.09	0.138
**1-I**	SOC (H-Only Mol. A)	2.352	1.996	2.142	2.168	–14.84	0.278
	SOC+SSC (H-Only Mol. A)	2.352	1.996	2.142	2.168	–15.07	0.285
	SOC (H-Only Mol. B)	2.369	2.012	2.119	2.172	–16.53	0.177
	SOC+SSC (H-Only Mol. B)	2.369	2.012	2.120	2.172	–16.81	0.180
	SOC (Full Opt (+))	2.022	2.324	2.179	2.179	+17.19	0.271
	SOC+SSC (Full Opt (+))	2.022	2.324	2.179	2.179	+17.37	0.273

[a] H-only geometries from a previously-reported orthorhombic structure (INUTUH01) were also considered for **1-Cl** (reported by Theopold et al.[1]).

[b] $g_{avg} = \sqrt{\frac{(g_x{}^2+g_y{}^2+g_z{}^2)}{3}}$, which is the powder average.

Table S18. Calculated effective one-electron SOC constants (ζ) for the **1-X** series.

	**1-F**			**1-Cl** [a]		**1-Br**			**1-I**		
	H-Only	Full (–)	Full (+)	H-Only	Full (+)	H-Only (Mol. A)	H-Only (Mol. B)	Full (+)	H-Only (Mol. A)	H-Only (Mol. B)	Full (+)
ζ (cm^{-1})	400.57	399.37	399.30	399.24	398.37	396.65	396.55	395.19	391.59	391.39	390.01

[a] The calculated values for ζ for the INUTUH01 molecule A and B H-only optimized structures (previously reported by Theopold et al.[1]) were 399.57 and 399.25 cm^{-1}, respectively.

Table S19. Linear combinations of the canonical *d*-orbitals to form trigonal symmetry-adapted linear-combination (SALC) orbitals in a C_{3v} point group.

**Symmetry**	***d*-Orbital SALC**
a_1	$\lvert z^2\rangle$
1e	$\sqrt{1/3}\,\lvert xz\rangle + \sqrt{2/3}\,\lvert x^2-y^2\rangle$<br>$-\sqrt{1/3}\,\lvert yz\rangle + \sqrt{2/3}\,\lvert xy\rangle$
2e	$\sqrt{2/3}\,\lvert xz\rangle - \sqrt{1/3}\,\lvert x^2-y^2\rangle$<br>$\sqrt{2/3}\,\lvert yz\rangle + \sqrt{1/3}\,\lvert xy\rangle$

Table S20. AILFT 3*d*-MO energies (cm^{-1}, first column) and orbital compositions (second column) from CASSCF/NEVPT2 calculations of the **1-X** series using the H-only optimized geometries. H-only optimized geometries for previously reported X-ray data for **1-Cl** (INUTUH01 reported by Theopold et al.[1]) are also included.

**1-F H-only**	
0.0	$-0.473\|yz\rangle + 0.307\|x^2-y^2\rangle + 0.805\|xy\rangle$
701.9	$0.361\|xz\rangle + 0.862\|x^2-y^2\rangle - 0.324\|xy\rangle$
3964.4	$1\|z^2\rangle$
5734.1	$0.792\|xz\rangle + 0.448\|yz\rangle - 0.310\|x^2-y^2\rangle$
6887.6	$0.427\|xz\rangle - 0.744\|yz\rangle - 0.424\|xy\rangle$
**1-Cl H-only**	
0.0	$-0.597\|yz\rangle + 0.802\|xy\rangle$
2.2	$-0.597\|xz\rangle - 0.802\|x^2-y^2\rangle$
1468.6	$1\|z^2\rangle$
5076.7	$-0.802\|xz\rangle + 0.597\|x^2-y^2\rangle$
5079.3	$-0.802\|yz\rangle - 0.597\|xy\rangle$
**1-Cl H-only (INUTUH01 Mol. A)**	
0.0	$-0.619\|xz\rangle - 0.769\|x^2-y^2\rangle$
345.7	$-0.581\|yz\rangle + 0.800\|xy\rangle$
1657.3	$1\|z^2\rangle$
4695.3	$-0.800\|yz\rangle - 0.587\|xy\rangle$
5342.2	$0.772\|xz\rangle - 0.624\|x^2-y^2\rangle$
**1-Cl H-only (INUTUH01 Mol. B)**	
0.0	$0.660\|xz\rangle + 0.738\|x^2-y^2\rangle$
580.4	$-0.554\|yz\rangle + 0.827\|xy\rangle$
1733.0	$1\|z^2\rangle$
4778.2	$+0.828\|yz\rangle + 0.555\|xy\rangle$
5802.3	$0.746\|xz\rangle - 0.659\|x^2-y^2\rangle$
**1-Br H-only (Mol. A)**	
0.0	$0.392\|xz\rangle - 0.562\|yz\rangle + 0.527\|x^2-y^2\rangle + 0.486\|xy\rangle$
715.2	$-0.483\|xz\rangle - 0.312\|yz\rangle - 0.538\|x^2-y^2\rangle + 0.616\|xy\rangle$
1322.4	$1\|z^2\rangle$
4499.2	$0.685\|xz\rangle + 0.445\|yz\rangle - 0.412\|x^2-y^2\rangle + 0.404\|xy\rangle$
5745.5	$0.376\|xz\rangle - 0.623\|yz\rangle - 0.503\|x^2-y^2\rangle - 0.462\|xy\rangle$
**1-Br H-only (Mol. B)**	
0.0	$0.473\|xz\rangle - 0.486\|yz\rangle + 0.578\|x^2-y^2\rangle + 0.445\|xy\rangle$
584.1	$-0.441\|xz\rangle - 0.405\|yz\rangle - 0.481\|x^2-y^2\rangle + 0.628\|xy\rangle$
1286.0	$1\|z^2\rangle$
4516.7	$0.580\|xz\rangle + 0.551\|yz\rangle - 0.377\|x^2-y^2\rangle + 0.465\|xy\rangle$
5538.9	$0.494\|xz\rangle - 0.542\|yz\rangle - 0.541\|x^2-y^2\rangle - 0.410\|xy\rangle$

**1-I H-only (Mol. A)**	
0.0	$-0.408\|xz\rangle + 0.605\|yz\rangle - 0.527\|x^2 - y^2\rangle - 0.427\|xy\rangle$
729.2	$0.499\|xz\rangle + 0.339\|yz\rangle + 0.473\|x^2 - y^2\rangle - 0.615\|xy\rangle$
931.5	$1\|z^2\rangle$
4297.3	$-0.651\|xz\rangle - 0.442\|yz\rangle + 0.388\|x^2 - y^2\rangle - 0.479\|xy\rangle$
5642.7	$-0.382\|xz\rangle + 0.566\|yz\rangle + 0.569\|x^2 - y^2\rangle + 0.455\|xy\rangle$
**1-I H-only (Mol. B)**	
0.0	$0.485\|xz\rangle - 0.542\|yz\rangle + 0.557\|x^2 - y^2\rangle + 0.387\|xy\rangle$
599.1	$-0.479\|xz\rangle - 0.422\|yz\rangle - 0.438\|x^2 - y^2\rangle + 0.632\|xy\rangle$
880.7	$1\|z^2\rangle$
4347.4	$0.584\|xz\rangle + 0.501\|yz\rangle - 0.383\|x^2 - y^2\rangle + 0.509\|xy\rangle$
5448.8	$0.439\|xz\rangle - 0.525\|yz\rangle - 0.590\|x^2 - y^2\rangle - 0.426\|xy\rangle$

Table S21. AILFT 3*d*-MO energies (cm^{-1}, first column) and orbital compositions (second column) from CASSCF/NEVPT2 calculations of the **1-X** series using the Full Opt geometries.

**1-F Full (–)**	
0.0	$-0.512\|xz\rangle - 0.842\|x^2-y^2\rangle$
840.2	$0.442\|yz\rangle - 0.887\|xy\rangle$
3717.9	$1\|z^2\rangle$
5930.7	$0.891\|yz\rangle + 0.441\|xy\rangle$
7415.9	$-0.850\|xz\rangle + 0.492\|x^2-y^2\rangle$
**1-F Full (+)**	
0.0	$-0.519\|yz\rangle + 0.855\|xy\rangle$
801.2	$-0.446\|xz\rangle - 0.885\|x^2-y^2\rangle$
3681.0	$1\|z^2\rangle$
6204.8	$-0.875\|xz\rangle + 0.400\|x^2-y^2\rangle$
7151.0	$-0.855\|yz\rangle - 0.519\|xy\rangle$
**1-Cl Full (+)**	
0.0	$-0.619\|yz\rangle + 0.783\|xy\rangle$
358.8	$0.593\|xz\rangle + 0.795\|x^2-y^2\rangle$
1653.4	$1\|z^2\rangle$
5057.2	$0.804\|xz\rangle - 0.591\|x^2-y^2\rangle$
5768.9	$-0.785\|yz\rangle - 0.619\|xy\rangle$
**1-Br Full (+)**	
0.0	$0.549\|xz\rangle - 0.351\|yz\rangle + 0.650\|x^2-y^2\rangle + 0.390\|xy\rangle$
265.4	$-0.343\|xz\rangle - 0.536\|yz\rangle - 0.394\|x^2-y^2\rangle + 0.656\|xy\rangle$
1137.2	$1\|z^2\rangle$
4907.9	$0.410\|xz\rangle + 0.652\|yz\rangle - 0.324\|x^2-y^2\rangle + 0.549\|xy\rangle$
5468.8	$0.642\|xz\rangle - 0.404\|yz\rangle - 0.562\|x^2-y^2\rangle - 0.331\|xy\rangle$
**1-I Full (+)**	
0.0	$0.578\|xz\rangle - 0.375\|yz\rangle + 0.624\|x^2-y^2\rangle + 0.369\|xy\rangle$
222.2	$-0.368\|xz\rangle - 0.566\|yz\rangle - 0.374\|x^2-y^2\rangle + 0.631\|xy\rangle$
733.1	$1\|z^2\rangle$
4724.1	$0.395\|xz\rangle + 0.622\|yz\rangle - 0.339\|x^2-y^2\rangle + 0.585\|xy\rangle$
5219.9	$0.612\|xz\rangle - 0.389\|yz\rangle - 0.596\|x^2-y^2\rangle - 0.345\|xy\rangle$

Table S22. Table of deconvoluted NIR absorption peaks using Gaussian functions.

	Peak 1				
	b_0 (M^{-1} cm^{-1})	E_c (cm^{-1})	Γ (cm^{-1})	A (M^{-1} cm^{-2})	f_{osc}
**1-F**	–12.2	6449(7)	963(15)	20715(368)	0.0000895(16)
**1-Cl**	–8.0	5037(5)	822(14)	31008(674)	0.000134(3)
**1-Br**	–14.6	4829(7)	782(22)	44411(1503)	0.000192(6)
**1-I**	–11.7	4520(9)	*800* [a]	44364(622)	0.000192(3)

	Peak 2				
	b_0 (M^{-1} cm^{-1})	E_c (cm^{-1})	Γ (cm^{-1})	A (M^{-1} cm^{-2})	f_{osc}
**1-F**	–12.2	8599(4)	2223(12)	118638(515)	0.000512(2)
**1-Cl**	–8.0	6611(4)	1857(10)	192173(860)	0.000830(4)
**1-Br**	–14.6	6326(5)	1804(12)	280067(1558)	0.001210(7)
**1-I**	–11.7	5984(2)	1759(6)	310460(848)	0.001341(4)

[a] Value was fixed at an estimated value in order to obtain a reasonable fit.

AOM/LFT/AILFT Tables:

Table S23. Two possible term assignments of the experimental bands used in LFT fitting (values in cm^{-1}). Note that the term assignments for the C_{3v} model are inconsistent with the AILFT results (*vide infra*).

Model	Term Number	1-F	1-Cl	1-Br	1-I	Weighting
C_{3v} Symmetry	1 [^{5}E(E)]	0	0	0	0	1
	6 [^{5}E(E)]	0	0	0	0	0
	11 [$^5A_1(T_2)$]	6449	5037	4829	4520	1
	16 [$^5E(T_2)$]	8599	6611	6326	5984	0.5
	21 [$^5E(T_2)$]	8599	6611	6326	5984	0.5
C_s Symmetry	1 [^{5}E(E)] ^{a}	0	0	0	0	1
	6 [^{5}E(E)]	---	---	---	---	0
	11 [$^5A_1(T_2)$]	---	---	---	---	0
	16 [$^5E(T_2)$]	6449	5037	4829	4520	1
	21 [$^5E(T_2)$]	8599	6611	6326	5984	1

^{a} Assignment for use of parameters derived from AILFT analysis. In C_s symmetry, the $^5A_1$ term becomes $^5A'$ while all the ^{5}E terms become either $^5A'$ or $^5A''$ (which is which is inconsequential, Scheme 2). Symmetry labels corresponding to C_{3v} symmetry are retained for clarity only.

Table S24. AOM parametrization of the Fe–ligand interactions in the **1-X** series for H-only optimized geometries (NEVPT2 energies) and the corresponding Racah parameters.^{a}

	1-F	1-Cl ^{c}		1-Br		1-I	
	H-Only	H-Only (Mol. A)	H-Only (Mol. B)	H-Only (Mol. A)	H-Only (Mol. B)	H-Only (Mol. A)	H-Only (Mol. B)
ε_σ(X)	5261	2667	2768	2237	2063	2016	1566
ε_π(X)	2091	202.1	265.1	–59.0	–118.1	–451.7	–537.8
ε_σ(N)	3749	3947	4070	4034	3921	4242	3950
$\varepsilon_{\pi s}$(N)	508.7	759.5	811.8	877.5	774.7	1137	854.9
σ_{SD}	104.7	97.08	149.6	183.9	161.8	191.1	172.3
B	935.1	922.8	923.0	926.6	925.8	926.7	925.8
C	3651	3646	3637	3647	3648	3646	3647
C/B	3.904	3.951	3.941	3.936	3.941	3.934	3.939
β_B ^{b}	0.917	0.905	0.905	0.908	0.908	0.909	0.908
β_C ^{b}	0.921	0.920	0.917	0.920	0.920	0.920	0.920

^{a} All values are in cm^{-1}, except C/B and β ratios, which are dimensionless.

^{b} $\beta_B = B_{\mathrm{complex}}/B_{\mathrm{free-ion}}$ and $\beta_C = C_{\mathrm{complex}}/C_{\mathrm{free-ion}}$ are the nephelauxetic ratios. The free-ion Racah parameters for Fe(II) are: $B_{\mathrm{free-ion}}$ = 1020 cm^{-1} and $C_{\mathrm{free-ion}}$ = 3965 cm^{-1}.[50]

^{c} No AOM parameters are obtainable for the trigonal symmetry crystal structure of **1-Cl**, so both molecules from a previously-reported[1] X-ray structure (INUTUH01) were used instead for comparison. The Racah parameters for the H-only optimized geometry (from the X-ray data for **1-Cl** reported herein) were: B = 921.7 cm^{-1}, C = 3636 cm^{-1}, C/B = 3.945, β_B = 0.904, and β_C = 0.917.

Table S25. Fit to NIR bands using AOM parameters derived from AILFT analysis; all values in cm^{-1}. Fit parameters are given in "LFT using AILFT" section in Table S26.

**Term Number**	**1-F** [a]	**1-Cl**	**1-Br**	**1-I**
6 [$^5E(E)$ in C_{3v}]				
Expt.	(Not Observed)			
Calc.	179.6(145.7)	108.4	77.07	56.27
11 [$^5A_1(T_2)$ in C_{3v}]				
Expt.	(Not Observed)			
Calc.	5542(5514)	5406	5471	5243
16 [$^5E(T_2)$ in C_{3v}]				
Expt.	6449	5037	4829	4520
Calc.	6449(6449)	5921	5943	5808
21 [$^5E(T_2)$ in C_{3v}]				
Expt.	8599	6611	6326	5984
Calc.	8599(8599)	6611	6326	5983

[a] Values in parentheses are for the **1-F** (–) isomer, while all other values are for the (+) isomers.

[b] This complex has crystallographically-imposed three-fold symmetry, so the real and ideal θ angles are the same.

Table S26. Comparison of classical LFT fits (from fitting the deconvoluted NIR absorption spectra band energies) assuming idealized C_{3v} symmetry with fits derived from AILFT (NEVPT2) on the Full Opt geometries (C_s symmetry). Italicized values were fixed, while all other values were refined freely. Values for H-only optimized geometries are included in the captions.

Model	AOM parameter	1-F [a]	1-Cl	1-Br	1-I
**(C_{3v} Symmetry)**	**Classical LFT**				
	ε_σ(X)	6444	5017	4795	4475
σ-Bonding Only	ε_σ(N)	5734	4414	4229	4004
	ε_σ(X)	7137	5420	5135	4668
Halide π-Bonding	ε_π(X)	2486	1273	1018	540.0
	ε_σ(N) [b]	*5000*	*4100*	*4000*	*3900*
**(C_s Symmetry)**	**AILFT (NEVPT2)**				
	ε_σ(X)	4983(5090)	2474	1945	1584
σ-Bonding and	ε_π(X)	1912(1929)	180.6	–242.5	–626.3
π-Bonding	ε_σ(N)	4104(4160)	4084	4121	4157
	$\varepsilon_{\pi s}$(N)	530.4(603.1)	638.8	727.3	867.9
**(C_s Symmetry)**	**LFT using AILFT**				
	ε_σ(X)	8003(7735) [d]	5261 [e]	5075 [f]	4688 [g]
Halide π-Bonding	ε_π(X)	706.5(1294)	–1582	–1526	–1450
	ε_σ(N) [b, c]	*4100*	*4100*	*4100*	*4000*

[a] Values in parentheses are for the **1-F** (–) isomer, while all other values are for the (+) isomers.

[b] No π-bonding by the pyrazolyl N-atom donors was included for the classical LFT models as this is overparameterization given the simple NIR absorption spectrum with only two bands.

[c] The value for ε_σ(N) was fixed at 4100 cm^{-1}, given the AILFT results produced values that were relatively constant across the **1-X** series and close to this value. The exception is for **1-I**, where the value for ε_σ(N) was fixed instead at 4000 cm^{-1}, since a value of 4100 cm^{-1} led to physically unreasonable values for ε_σ(I) and ε_π(I).

[d] The H-only optimized geometry for **1-F** fit gave ε_σ(F) = 7971 cm^{-1} and ε_π(F) = 1034 cm^{-1}.

[e] Only the higher energy band (6611 cm^{-1}) was fitted; the lower energy band (5037 cm^{-1}) value converged to a value that was too high by 884.4 cm^{-1} (18%). The fit for the H-only optimized geometry for **1-Cl** converged to values of ε_σ(Cl) = 4891 cm^{-1} and ε_π(Cl) = –1717 cm^{-1} by modeling the average band energy (5824 cm^{-1}).

[f] Only the higher energy band (6326 cm^{-1}) was fitted; the lower energy band (4829 cm^{-1}) value converged to a value that was too high by 1114 cm^{-1} (23%). The fit for the H-only optimized geometry (average of each pair of θ values; see Table S12) for **1-Br** converged to values of ε_σ(Br) = 3711 cm^{-1} and ε_π(Br) = –933.1 cm^{-1}.

[g] Only the higher energy band (5984 cm^{-1}) was fitted; the lower energy band (4520 cm^{-1}) value converged to a value that was too high by 1288 cm^{-1} (28%). The fit for the H-only optimized geometry (average of each pair of θ values; see Table S12) for **1-I** converged to values of ε_σ(I) = 2123 cm^{-1} and ε_π(I) = –833.0 cm^{-1}.

Vibronic Coupling and Jahn Teller Analysis Table:

Table S27. M–N bond distances (R, in Å) and X–M–N$_{Tp}$ bond angles (θ, in °) of [M^{II}X(Tp^{tBu,Me})], M = Fe in comparison with M = Co computed using X-ray data along with their distortions from the idealized trigonal C_{3v} geometry quantified using the effective interaction normal modes depicted in Scheme 3 and defined by Equations 1 and 2 in the main text. For **1-Br** and **1-I**, both molecules per unit cell were used from each crystal structure for the comparisons, with the values in parentheses corresponding to molecule B. Previously reported X-ray data for **1-Cl** (INUTUH01 reported by Theopold et al.[1]) were used instead of the X-ray data reported herein due to the crystallographically-imposed trigonal symmetry precluding Jahn-Teller analysis.

**X =**	**F**		**Cl**		**Br**		**I**	
**M =**	**Co** ^{a,b}	**Fe**	**Co** ^{c}	**Fe A (B)**	**Co A** ^{a} **(B)**	**Fe A (B)**	**Co A** ^{a} **(B)**	**Fe A (B)**
R_{MN_1}	2.031<br>[2.003]	2.113	2.023	2.104<br>(2.092)	2.052<br>(2.034)	2.110<br>(2.119)	2.066<br>(2.039)	2.122<br>(2.114)
R_{MN_3}	2.028<br>[2.002]	2.108	2.023	2.097<br>(2.084)	2.051<br>(2.031)	2.098<br>(2.088)	2.063<br>(2.018)	2.087<br>(2.087)
R_{MN_5}	2.038<br>[2.001]	2.079	2.036	2.065<br>(2.060)	2.022<br>(2.053)	2.058<br>(2.060)	2.009<br>(2.053)	2.056<br>(2.063)
θ_{XMN_1}	121.8<br>[121.9]	118.4	121.3	120.4<br>(118.4)	118.8<br>(121.3)	119.2<br>(117.9)	118.6<br>(124.2)	119.4<br>(117.8)
θ_{XMN_3}	121.1<br>[122.1]	122.9	121.3	122.4<br>(120.7)	119.4<br>(124.2)	120.0<br>(122.8)	118.8<br>(120.9)	120.3<br>(121.5)
θ_{XMN_5}	123.3<br>[121.7]	132.4	121.1	126.5<br>(130.0)	126.0<br>(117.7)	130.6<br>(128.4)	126.2<br>(117.4)	128.9<br>(128.5)
q_{str}	–0.008<br>[0.001]	0.026	–0.011	0.029<br>(0.023)	0.024<br>(–0.017)	0.038<br>(0.036)	0.045<br>(–0.020)	0.040<br>(0.031)
q_{bend}	–0.056<br>[0.008]	–0.353	0.007	–0.152<br>(–0.302)	–0.200<br>(0.147)	–0.326<br>(–0.242)	–0.220<br>(0.148)	–0.268<br>(–0.263)
$\frac{q_{bend}(Fe)}{q_{bend}(Co)}$	6.36		22.0 ^{d}<br>(43.5)		1.64<br>(1.64)		1.22<br>(1.78)	

^{a} Data for [Co^{II}FTp(^{tBu,Me})], [Co^{II}BrTp(^{tBu,Me})], and [Co^{II}ITp(^{tBu,Me})] were taken from Telser and Fielder et al.[51]

^{b} The values in brackets pertain to data from DFT optimization of the entire [Co^{II}FTp(^{tBu,Me})] complex, which show a regular trigonal geometry around cobalt as expected for the non-degenerate $^{4}A_2(C_{3v})$ Jahn-Teller inactive ground state.

^{c} Data for [Co^{II}ClTp(^{tBu,Me})] were taken from Ferrence and Beitelman.[52] This was to avoid structures with trigonal symmetry.

^{d} The q_{bend} ratios are artificially large due to the near-trigonal symmetry of the X-ray structure for [Co^{II}ClTp(^{tBu,Me})].

Tables of *xyz* Coordinates for DFT-Optimized Geometries:

Table S28. H-only optimized geometry for **1-F**.

Fe	0.00000000	0.00000000	0.00000000
F	0.20244954	-0.16943286	1.79428166
N	-0.68422821	1.28912602	-2.53648851
N	-0.86515202	1.47613345	-1.18020142
N	1.46657863	-0.04171427	-2.56247227
N	1.73319083	0.00000011	-1.20908085
N	-0.74877184	-1.25321500	-2.55815907
N	-0.89804090	-1.48435492	-1.19696092
C	-1.22362158	2.32323981	-3.20178463
C	-1.76861563	3.18933872	-2.28015365
C	-1.52765344	2.62378132	-1.02461063
C	2.62920771	-0.03057185	-3.25184344
C	3.65989016	0.01348641	-2.33772313
C	3.06281757	0.03945490	-1.06993818
C	-1.35427618	-2.24498034	-3.23944213
C	-1.90414405	-3.13093144	-2.32648320
C	-1.59715090	-2.61636862	-1.06323919
C	-1.22875393	2.42659373	-4.70116503
C	-1.89962470	3.15763938	0.35211137
C	-2.89260093	4.30619127	0.17280474
C	-0.64030696	3.67350509	1.04296944
C	-2.56465238	2.07731118	1.18757139
C	2.68008062	-0.06397569	-4.74880539
C	3.71536330	0.09799404	0.29952044
C	5.22491250	0.16406179	0.13605213
C	3.24724898	1.35475142	1.03558568
C	3.35563327	-1.14114482	1.10903694
C	-1.38455140	-2.30119497	-4.72974131
C	-1.91204342	-3.19589703	0.29528098
C	-2.88190302	-4.37078667	0.12775182
C	-2.60825191	-2.16423846	1.16946189
C	-0.62554340	-3.68313587	0.92879615
B	0.00000017	-0.00000000	-3.06083661
H	-2.28597309	4.11575306	-2.49959911
H	4.71907853	0.02747341	-2.56745233
H	-2.45730061	-4.03227194	-2.56125125
H	-0.20862310	2.42220703	-5.10985698
H	-1.71825925	3.36029215	-5.00231768
H	-1.76973472	1.58690385	-5.15961204
H	-2.45425367	5.13879898	-0.39620783
H	-3.19150967	4.69833076	1.15575413
H	-3.80100785	3.97208833	-0.34876749
H	0.08721947	2.86374009	1.19811394
H	-0.88740097	4.09431489	2.02946881

H	-0.15431368	4.45804829	0.44534549
H	-3.45536163	1.67550505	0.68389779
H	-2.87507668	2.48937950	2.15930098
H	-1.88280165	1.23940533	1.39956667
H	2.17443388	-0.95342879	-5.15097206
H	3.72502689	-0.08250531	-5.08138700
H	2.19433040	0.81716111	-5.19241111
H	5.61721634	-0.72679554	-0.37699210
H	5.70614680	0.21725712	1.12343342
H	5.53496553	1.05187182	-0.43503588
H	3.75105185	1.43325814	2.01085040
H	2.16597355	1.32820466	1.22949042
H	3.47513320	2.26039177	0.45491950
H	3.65223471	-2.05812095	0.57869344
H	2.27878525	-1.19075404	1.31832547
H	3.87682658	-1.12372026	2.07847971
H	-1.87222125	-1.41576081	-5.16227624
H	-1.93894970	-3.18996040	-5.05484306
H	-0.37220454	-2.35524311	-5.15605508
H	-3.82102222	-4.04981141	-0.34567656
H	-3.12611411	-4.79282763	1.11311495
H	-2.44763840	-5.17781496	-0.48086944
H	-1.95295897	-1.31259266	1.40274771
H	-2.90107064	-2.61610671	2.12905519
H	-3.51405026	-1.78093303	0.67859041
H	-0.12875252	-4.43042670	0.29282696
H	-0.82646230	-4.14656623	1.90725012
H	0.07815085	-2.85514486	1.09515115
H	-0.01260583	0.01489931	-4.26245792

Table S29. Fully optimized geometry for (–) isomer of **1-F**.

Fe	0.00000000	0.00000000	0.00000000
F	-0.28928645	0.07072307	1.80282465
N	-0.73001692	1.26117854	-2.51651485
N	-0.87084662	1.48502163	-1.16970374
N	1.45889752	-0.00107565	-2.49168730
N	1.68418491	0.00000000	-1.14012093
N	-0.72918128	-1.26240627	-2.50807112
N	-0.86071244	-1.47639250	-1.15939982
C	-1.30795085	2.27414649	-3.21850467
C	-1.83668514	3.16910738	-2.29261260
C	-1.54179365	2.64280797	-1.01729787
C	2.64300014	-0.02206457	-3.15172019
C	3.65846181	-0.03440126	-2.19082208
C	3.01753969	-0.02237218	-0.93974399
C	-1.30617163	-2.28329858	-3.19633460
C	-1.82627081	-3.17370475	-2.25865725
C	-1.52500964	-2.63628427	-0.99089598
C	-1.32117464	2.33179141	-4.70974529
C	-1.88939297	3.19635140	0.34946416
C	-2.60210614	4.54868750	0.19109611
C	-0.60182810	3.39698992	1.17502920
C	-2.82708182	2.21187560	1.08064935
C	2.74770798	-0.03058785	-4.64012914
C	3.59731036	-0.02601519	0.45849866
C	5.13024311	-0.09806313	0.38475023
C	3.17774618	1.26675634	1.18927470
C	3.06496966	-1.24767405	1.23580934
C	-1.32855657	-2.35303512	-4.68683825
C	-1.85866132	-3.17305948	0.38562201
C	-2.56160339	-4.53345918	0.25046479
C	-2.79894150	-2.18621972	1.10961297
C	-0.56534942	-3.35260786	1.20674125
B	0.00000005	0.00000004	-3.02778874
H	-2.36631224	4.08665635	-2.52104386
H	4.72424375	-0.05123800	-2.38720467
H	-2.35313704	-4.09539778	-2.47678543
H	-0.30406823	2.35421508	-5.12754850
H	-1.84383150	3.23900731	-5.03670421
H	-1.83523802	1.46430644	-5.14884261
H	-1.96415364	5.28275323	-0.32273128
H	-2.85336853	4.95283074	1.18197897
H	-3.53798769	4.44575022	-0.37738593
H	-0.11306414	2.43658260	1.38476676
H	-0.84379299	3.86130840	2.14260165
H	0.10711649	4.04817446	0.64383473

H	-3.73613224	2.02826460	0.48995871
H	-3.12458824	2.62757234	2.05504421
H	-2.32595748	1.25349598	1.26583761
H	2.27172506	-0.92093255	-5.07563656
H	3.80354170	-0.02761279	-4.93651685
H	2.26238218	0.84962618	-5.08517392
H	5.46406862	-1.01524226	-0.12202948
H	5.55120619	-0.10028017	1.39993040
H	5.54834761	0.76582631	-0.15216333
H	3.61572858	1.29041439	2.19785624
H	2.08563799	1.32153676	1.29955612
H	3.51524012	2.15731156	0.64048245
H	3.29228936	-2.18334960	0.70601142
H	1.97682600	-1.18390472	1.37885132
H	3.52508474	-1.28934415	2.23390650
H	-1.85953628	-1.49616502	-5.12659868
H	-1.83853027	-3.27058950	-5.00443811
H	-0.31369035	-2.35989871	-5.10973842
H	-3.50110035	-4.44511274	-0.31422221
H	-2.80378069	-4.92588462	1.24824077
H	-1.92035660	-5.26918012	-0.25669017
H	-2.30542896	-1.22072482	1.28105469
H	-3.08694029	-2.59211870	2.09084188
H	-3.71294996	-2.01534359	0.52295510
H	0.14529070	-4.00747084	0.68255194
H	-0.80007169	-3.80333310	2.18240927
H	-0.07861020	-2.38750662	1.40154338
H	0.01766410	-0.00541332	-4.22863916

Table S30. Fully optimized geometry for (+) isomer of **1-F**.

Fe	0.00000000	0.00000000	0.00000000
F	0.27665205	0.00431406	1.80633393
N	-0.71364613	1.27080257	-2.49666291
N	-0.83638649	1.47023478	-1.14608766
N	1.45807520	-0.01667055	-2.51972053
N	1.73146210	0.00000002	-1.17427418
N	-0.73936306	-1.25657808	-2.49533330
N	-0.82972372	-1.47314322	-1.14443592
C	-1.28880019	2.30165651	-3.16705714
C	-1.79351202	3.18888100	-2.21417345
C	-1.49403916	2.63157559	-0.95705573
C	2.61908516	-0.05146698	-3.23051989
C	3.66433433	-0.05465001	-2.31253073
C	3.07037632	-0.02172344	-1.03244286
C	-1.35184140	-2.26670520	-3.16364425
C	-1.84548843	-3.15926172	-2.20988241
C	-1.50063518	-2.62651969	-0.95373978
C	-1.32797585	2.38573359	-4.65639367
C	-1.79131283	3.15021745	0.43393367
C	-2.58887181	4.46009142	0.33979643
C	-0.46517978	3.40794091	1.18069448
C	-2.61838297	2.10379551	1.20954741
C	2.66229250	-0.08292603	-4.72194518
C	3.74085837	-0.01045097	0.32672281
C	5.26745814	-0.03304706	0.14601408
C	3.34198225	1.26728197	1.09327627
C	3.31262864	-1.25519419	1.13199370
C	-1.43644750	-2.32766434	-4.65218840
C	-1.75659982	-3.16901462	0.43647578
C	-2.60786972	-4.44506247	0.34430267
C	-2.50507616	-2.11718486	1.28086675
C	-0.40791747	-3.50251994	1.10998789
B	0.00000013	-0.00000005	-3.02658982
H	-2.31331483	4.11797168	-2.41759036
H	4.72196697	-0.07841272	-2.54782223
H	-2.38837949	-4.07520139	-2.41224674
H	-0.31694673	2.40487567	-5.08840085
H	-1.84762560	3.30237387	-4.96050039
H	-1.85579483	1.52857261	-5.09849782
H	-2.02248036	5.23850751	-0.19198520
H	-2.80864839	4.83335370	1.34983370
H	-3.54518431	4.31019451	-0.18219019
H	0.08860845	2.47206612	1.33793491
H	-0.66870948	3.84261541	2.17053546
H	0.17402641	4.10394650	0.61925037

H	-3.55534775	1.87139811	0.68387684
H	-2.86362111	2.48409632	2.21194798
H	-2.05030107	1.17243324	1.34028856
H	2.14594249	-0.96612481	-5.12558170
H	3.70577337	-0.11324675	-5.05843224
H	2.18526044	0.80383688	-5.16432410
H	5.59534276	-0.94005356	-0.38265906
H	5.75664942	-0.02079846	1.13032612
H	5.61950258	0.84310417	-0.41790963
H	3.85653690	1.29935089	2.06539793
H	2.26254526	1.29058953	1.28970237
H	3.61852364	2.16683515	0.52456271
H	3.53749292	-2.17649994	0.57557428
H	2.23969751	-1.22952561	1.36145007
H	3.85513716	-1.28736054	2.08885956
H	-1.95485771	-1.45100539	-5.06610879
H	-1.98826685	-3.22614735	-4.95383745
H	-0.43973010	-2.36605487	-5.11510398
H	-3.58170958	-4.24543836	-0.12614527
H	-2.79324214	-4.84100650	1.35267699
H	-2.09659443	-5.22674195	-0.23612396
H	-1.87880667	-1.23218829	1.46274762
H	-2.76384665	-2.53934894	2.26310445
H	-3.43184899	-1.79787906	0.78358894
H	0.15410531	-4.23839595	0.51742854
H	-0.57933976	-3.92007943	2.11314397
H	0.21135478	-2.60265335	1.22439909
H	-0.01824319	0.00139337	-4.22717828

Table S31. H-only optimized geometry for **1-Cl**.

Fe	0.00000000	0.00000000	0.00000000
Cl	0.00000001	-0.00000000	2.23300300
N	-0.73021619	1.26478436	-2.49873400
N	-0.86375410	1.49608198	-1.14980099
N	1.46042999	0.00000127	-2.49873401
N	1.72750900	0.00000004	-1.14980101
N	-0.73022784	-1.26476164	-2.49873400
N	-0.86376789	-1.49605802	-1.14980099
C	-1.30901957	2.26731203	-3.19678300
C	-1.82539442	3.16171042	-2.27894300
C	-1.53351276	2.65615007	-1.01773700
C	-1.34140527	2.32340619	-4.68721300
C	-1.86756509	3.23475161	0.34628900
C	-0.57695875	3.52476267	1.12263301
C	-2.62717602	4.55045111	0.15291100
C	2.61804599	-0.00000407	-3.19678302
C	3.65080600	-0.00000882	-2.27894302
C	3.06703600	-0.00000613	-1.01773702
C	2.68281798	-0.00000437	-4.68721301
C	3.73514601	-0.00000920	0.34628899
C	3.34099918	-1.26271139	1.12263300
C	5.25438001	-0.00001621	0.15291099
C	-1.30904046	-2.26728396	-3.19678300
C	-1.82542356	-3.16167759	-2.27894299
C	-1.53353723	-2.65611994	-1.01773699
C	-1.34142669	-2.32337783	-4.68721300
C	-1.86759491	-3.23471839	0.34628901
C	-2.76405443	-2.26202627	1.12263301
C	-2.62721797	-4.55041089	0.15291101
C	-2.76403358	2.26206775	1.12263301
C	3.34101083	1.26269661	1.12263299
C	-0.57699124	-3.52474134	1.12263301
B	0.00000000	0.00000000	-3.02793100
H	-0.00329057	0.00000080	-4.22664018
H	-2.35266812	4.07956884	-2.51166299
H	-0.32953270	2.34885568	-5.11651252
H	-1.87166071	3.22806385	-5.00850221
H	-0.01083118	2.60620406	1.32540586
H	-0.81679860	3.98327287	2.09350366
H	0.06858308	4.21704300	0.56306789
H	-2.02312758	5.29012387	-0.39280411
H	-2.87812125	4.98117602	1.13276053
H	4.70904230	-0.00001281	-2.51347235
H	2.19327135	-0.88535098	-5.11840544
H	3.73175453	-0.00000812	-5.00742534

H	2.26185460	-1.29783202	1.32043496
H	3.85308669	-1.28190571	2.09625042
H	3.62336235	-2.16733933	0.56499649
H	5.59202562	-0.89170134	-0.39533501
H	5.75297768	-0.00001864	1.13279957
H	-2.35270636	-4.07953075	-2.51166290
H	-1.85672101	-1.45277188	-5.11732335
H	-1.87169100	-3.22803042	-5.00850182
H	-2.25526512	-1.30727247	1.30887372
H	-3.02753943	-2.69117151	2.10066688
H	-3.69369496	-2.06155315	0.57049433
H	-3.56752555	-4.39773371	-0.39679034
H	-2.87816687	-4.98113340	1.13276062
H	-1.85670809	1.45280527	-5.11732353
H	-2.25525272	1.30730939	1.30887357
H	-3.02751495	2.69121526	2.10066690
H	-3.69367580	2.06160297	0.57049412
H	-3.56748481	4.39778248	-0.39679067
H	2.19327772	0.88534588	-5.11840520
H	2.26186650	1.29782698	1.32043487
H	3.85309859	1.28188633	2.09625035
H	3.62338200	2.16732196	0.56499638
H	5.59203384	0.89166599	-0.39533473
H	-0.32955440	-2.34883708	-5.11651246
H	-0.01085501	-2.60618821	1.32540616
H	-0.81683570	-3.98324909	2.09350366
H	0.06854404	-4.21702805	0.56306819
H	-2.02317645	-5.29008929	-0.39280414

Table S32. H-only optimized geometry for molecule A of the previously reported structure for **1-Cl** (INUTUH01; see Theopold et al.[1]).

Fe	0.00000000	0.00000000	0.00000000
Cl	-0.13600238	0.04582330	2.22301618
N	-0.71427363	-1.27456411	-2.48563582
N	-0.82521050	-1.55219962	-1.14381608
N	1.46313010	0.01442300	-2.48276969
N	1.73135955	0.00000015	-1.12582347
N	-0.75245933	1.25866468	-2.49528449
N	-0.87810838	1.52555806	-1.15329478
C	-1.42090659	-2.75384711	-1.03652446
C	-1.69409225	-3.24127532	-2.31400227
C	-1.23083360	-2.28460916	-3.20609192
C	-1.28181872	-2.27359329	-4.69541718
C	-1.73410789	-3.38876198	0.30005924
C	-2.40651602	-4.75025233	0.07887610
C	-2.70666393	-2.49586626	1.08023614
C	-0.44613453	-3.59865052	1.10725290
C	3.06591139	0.00190738	-0.99694870
C	3.64591045	0.01561588	-2.26764392
C	2.60407001	0.02807676	-3.16915554
C	2.64942274	0.05764598	-4.66265959
C	3.74884909	-0.00496542	0.35929884
C	5.26442804	-0.06204833	0.16062820
C	3.39668538	1.27021598	1.12679543
C	3.31405951	-1.23678509	1.16795397
C	-1.53506952	2.69280607	-1.05551343
C	-1.82492626	3.17082957	-2.32907969
C	-1.31476958	2.23566033	-3.22092795
C	-1.32586280	2.24975660	-4.70959721
C	-1.87543511	3.33525189	0.27542627
C	-2.58367861	4.67602760	0.04782377
C	-0.58115994	3.60278014	1.06781192
C	-2.80890172	2.41038619	1.06944390
B	0.00000005	0.00000000	-2.98591281
H	0.00561918	-0.00499753	-4.18514705
H	-2.17611057	-4.17664366	-2.57238778
H	-1.85299695	-1.41390142	-5.07400054
H	-1.76537021	-3.19001850	-5.05537784
H	-0.27804673	-2.21929969	-5.14035047
H	-3.34783160	-4.65088187	-0.48096624
H	-2.64050627	-5.20617777	1.05108462
H	-1.74926281	-5.44264190	-0.46764482
H	-2.26131775	-1.51829872	1.30798172
H	-2.97124119	-2.97054081	2.03684451
H	-3.63124427	-2.33177740	0.50800405

H	0.26656598	-4.22870074	0.55559993
H	-0.67714873	-4.09131168	2.06317474
H	0.04111910	-2.64200080	1.33886601
H	4.70226795	0.02271217	-2.50850522
H	2.15990057	-0.82324803	-5.10109646
H	3.69265661	0.07401082	-5.00028374
H	2.14388266	0.94735023	-5.06344726
H	5.56957971	-0.97009067	-0.37993114
H	5.76483016	-0.07092492	1.13932187
H	5.63462011	0.81189829	-0.39544997
H	3.68106243	2.16652177	0.55680280
H	3.92695360	1.29050411	2.09038989
H	2.32018966	1.32198502	1.34068233
H	2.23719655	-1.21449872	1.38310137
H	3.84261569	-1.25717910	2.13223878
H	3.54305987	-2.16589494	0.62653193
H	-2.34591109	4.08474775	-2.58846038
H	-0.30835133	2.27073906	-5.12645084
H	-1.85715049	3.14147506	-5.06400164
H	-1.82902334	1.36459277	-5.12476156
H	-1.94852581	5.37868451	-0.51105378
H	-2.81939077	5.13433473	1.01858153
H	-3.52801906	4.54951717	-0.50179929
H	-0.04901799	2.67023486	1.29601793
H	-0.81811905	4.09144678	2.02446831
H	0.09209201	4.25993867	0.49884922
H	-3.73540140	2.21948958	0.50845059
H	-3.07599034	2.87477984	2.03055339
H	-2.33183481	1.44670031	1.28941255

Table S33. H-only optimized geometry for molecule B of the previously reported structure for **1-Cl** (INUTUH01; see Theopold et al.[1]).

Fe	0.00000000	0.00000000	0.00000000
Cl	-0.28542829	-0.05301462	2.20927961
N	-0.73501048	-1.26179954	-2.48961125
N	-0.81506764	-1.54915659	-1.14483635
N	1.45549216	-0.00226137	-2.46175102
N	1.73994858	0.00000015	-1.10277249
N	-0.72618230	1.26937517	-2.47596409
N	-0.77995716	1.56242954	-1.13648144
C	3.06079114	0.00752803	-0.96544181
C	3.65817983	-0.00145958	-2.22985067
C	2.61900759	-0.00302208	-3.13477120
C	2.68398829	-0.02484637	-4.62915230
C	3.72573455	0.01923904	0.39607014
C	5.24629816	0.06961234	0.24070379
C	3.34947461	-1.24831499	1.16326506
C	3.27861384	1.27625217	1.16767663
C	-1.40958874	2.73526382	-1.02072815
C	-1.79352095	3.19057487	-2.28862006
C	-1.33817409	2.23642138	-3.18720296
C	-1.48878447	2.18865031	-4.67187808
C	-1.64588227	3.41302010	0.31848518
C	-2.25796879	4.79498912	0.11279318
C	-2.62092643	2.56862717	1.15614828
C	-0.30481231	3.57325980	1.06747355
C	-1.47051777	-2.70785679	-1.04470746
C	-1.81805217	-3.16085141	-2.31902903
C	-1.34184901	-2.22645276	-3.20713538
C	-1.43806176	-2.19148374	-4.69013828
C	-1.71777641	-3.40433213	0.28328683
C	-2.33488084	-4.79045664	0.04957835
C	-2.68838412	-2.57563949	1.13917134
C	-0.37475927	-3.58696650	1.02831526
B	0.00000001	0.00000000	-2.97779559
H	0.01833187	0.00871479	-4.17821673
H	4.71594565	-0.00245770	-2.46399273
H	2.17210161	-0.90539136	-5.04218639
H	3.73135718	-0.05194018	-4.95314757
H	2.21128664	0.86471252	-5.06942523
H	5.63099695	-0.80967822	-0.29733953
H	5.71725034	0.08679792	1.23354365
H	5.56752648	0.97204983	-0.29986355
H	2.26739875	-1.29980567	1.35022157
H	3.85387384	-1.26553665	2.14081308
H	3.64677644	-2.14805855	0.60536355

H	3.53079516	2.18803592	0.60791015
H	3.78013491	1.31744875	2.14567714
H	2.19525876	1.26795762	1.35152912
H	-2.32409946	4.10324606	-2.53167418
H	-0.51695757	2.13855544	-5.18287561
H	-2.01216352	3.08845524	-5.01724049
H	-2.07050994	1.31121102	-4.98980150
H	-1.60279224	5.44796935	-0.48313535
H	-2.41632081	5.27692024	1.08796310
H	-3.23444241	4.73538608	-0.39050211
H	-3.58093122	2.45262580	0.63185086
H	-2.81307479	3.06078663	2.12133285
H	-2.21422390	1.57177393	1.36677945
H	0.14929892	2.59869843	1.28841426
H	-0.46944721	4.08689788	2.02585619
H	0.40552288	4.16453868	0.47191121
H	-2.35219948	-4.07001733	-2.56891267
H	-1.98752585	-1.30634636	-5.04277798
H	-1.96770265	-3.08337904	-5.04710875
H	-0.44748609	-2.16942587	-5.16687442
H	-3.30748733	-4.71951133	-0.45951296
H	-2.50010158	-5.28528110	1.01701374
H	-1.67520024	-5.43339697	-0.55162973
H	-2.28197109	-1.58195479	1.36392570
H	-2.87524496	-3.08354785	2.09720859
H	-3.65168102	-2.45193009	0.62239425
H	0.33174216	-4.16673658	0.41708332
H	-0.54129737	-4.12437970	1.97341715
H	0.08288309	-2.62065016	1.27433681

Table S34. Fully optimized geometry for (+) isomer of **1-Cl**.

Fe	0.00000000	0.00000000	0.00000000
Cl	0.16263759	-0.01712046	2.20624421
N	-0.72647227	1.26481953	-2.47339193
N	-0.85913595	1.48209333	-1.12342290
N	1.45816534	-0.00184498	-2.49332788
N	1.74688846	0.00000006	-1.14935490
N	-0.73470639	-1.25901855	-2.47444152
N	-0.87978008	-1.46958525	-1.12481188
C	-1.31267482	2.27448633	-3.16368901
C	-1.83506794	3.16901250	-2.22916551
C	-1.53660497	2.63970390	-0.96071702
C	-1.34990521	2.33478554	-4.65411858
C	-1.86770301	3.19956564	0.40622923
C	-0.56207224	3.51089987	1.16748540
C	-2.70835372	2.17471242	1.19512786
C	-2.67770801	4.49716729	0.24998785
C	2.60875251	0.01417570	-3.21926291
C	3.66555509	0.02538745	-2.31572872
C	3.09085333	0.01691818	-1.02786523
C	2.63689506	0.01839419	-4.71139826
C	3.79740326	0.02768559	0.31210783
C	3.42598125	-1.24089890	1.10688040
C	3.40099007	1.29422307	1.09838175
C	5.31988057	0.04152195	0.09202379
C	-1.33175584	-2.26178920	-3.16584000
C	-1.87447300	-3.14471989	-2.23218799
C	-1.57725749	-2.61551246	-0.96325882
C	-1.35707125	-2.32740410	-4.65625341
C	-1.92899438	-3.16592750	0.40228328
C	-2.75177795	-2.12220175	1.18459771
C	-0.63537900	-3.50813486	1.17129461
C	-2.76684886	-4.44539342	0.24251362
B	0.00000000	0.00000000	-2.99453213
H	-2.36758029	4.08624500	-2.45089119
H	-0.33923135	2.35581662	-5.08632080
H	-1.87685543	3.24245438	-4.97210601
H	-1.87112434	1.46721984	-5.08333621
H	0.02617989	2.60092595	1.34084170
H	-0.79457040	3.94828565	2.14962894
H	0.05517901	4.22659664	0.60566854
H	-3.63554601	1.93193975	0.65660414
H	-2.97349696	2.58432519	2.18071462
H	-2.14747605	1.24671949	1.36358556
H	-2.10588980	5.26578781	-0.28991539
H	-2.92638777	4.89745454	1.24278631

H	-3.62001228	4.32157345	-0.28913590
H	-0.01490984	0.00087635	-4.19469289
H	4.71988122	0.03839070	-2.56512250
H	2.16809864	-0.88280017	-5.13306878
H	3.67720831	0.05446032	-5.05688829
H	2.10681942	0.88714342	-5.12744342
H	2.35027629	-1.28558414	1.31456555
H	3.95142644	-1.24695342	2.07341848
H	3.71481309	-2.14399090	0.54993160
H	3.66601828	2.19877211	0.53216408
H	3.92985966	1.31925429	2.06259709
H	2.32515920	1.31623723	1.30983273
H	5.65388771	-0.84407829	-0.46782122
H	5.83054184	0.03778823	1.06525971
H	5.64124179	0.94031250	-0.45423384
H	-2.41919795	-4.05459996	-2.45464195
H	-1.85264433	-1.44927190	-5.09399453
H	-1.90250137	-3.22368943	-4.97536360
H	-0.34298568	-2.37465998	-5.07842539
H	-2.17813176	-1.20035166	1.34306007
H	-3.01955502	-2.51895224	2.17473566
H	-3.67767068	-1.87171024	0.64711744
H	-0.03408452	-4.24061217	0.61384579
H	-0.88480125	-3.93803135	2.15258441
H	-0.02426723	-2.61372086	1.34728719
H	-3.70223683	-4.24863952	-0.30113506
H	-3.02811847	-4.84022822	1.23427925
H	-2.20967812	-5.22655930	-0.29473924

Table S35. H-only optimized geometry for molecule A of **1-Br**.

Fe	0.00000000	0.00000000	0.00000000
Br	0.10443016	-0.30000060	2.36910330
N	-0.83642179	-1.19680978	-2.51072870
N	-1.06540318	-1.39823173	-1.16741924
N	1.46239230	-0.11420745	-2.48376945
N	1.76253144	0.00000030	-1.13772843
N	-0.63733818	1.29807128	-2.46976722
N	-0.68082374	1.58724834	-1.11902846
C	-1.56230592	-2.09451483	-3.23699317
C	-2.25105957	-2.89520497	-2.36952169
C	-1.92216037	-2.42698519	-1.08817361
C	2.60741590	-0.15963704	-3.19543087
C	3.65393180	-0.07369751	-2.31743663
C	3.10794024	0.04640694	-1.04512435
C	-1.21283270	2.31351674	-3.14926404
C	-1.61116327	3.27802258	-2.25140996
C	-1.25773094	2.79584191	-0.98780453
C	-1.53660119	-2.10985768	-4.74048702
C	-2.45177493	-2.97955036	0.23420292
C	-3.44197930	-4.12329095	-0.00322943
C	-3.17033570	-1.85881896	1.01025966
C	-1.26455306	-3.50747432	1.07505988
C	2.61812857	-0.27663033	-4.68975787
C	3.86130030	0.18558008	0.26844833
C	3.76601199	-1.13213537	1.05143482
C	3.24302812	1.31304361	1.10296712
C	5.32469345	0.50972682	-0.00770325
C	-1.32338390	2.32602607	-4.64797606
C	-1.44044808	3.46051654	0.35878404
C	-1.84044608	4.90958771	0.14438482
C	-2.52119792	2.73655743	1.15931373
C	-0.11242071	3.44485980	1.14044170
B	-0.00000014	0.00000026	-2.98783959
H	-2.91530572	-3.71216393	-2.62185110
H	4.70590403	-0.07895453	-2.57835334
H	-2.08902814	4.22019040	-2.49050770
H	-1.91628350	-1.16839522	-5.16215364
H	-2.16595218	-2.92931870	-5.10858101
H	-0.51877164	-2.25648296	-5.12838487
H	-3.80706966	-4.49560689	0.96418419
H	-2.97071096	-4.96587187	-0.53057179
H	-4.31342373	-3.79281605	-0.58786123
H	-4.01387197	-1.45895431	0.42836075
H	-2.48644579	-1.03248998	1.24437411
H	-3.55827675	-2.24716062	1.96342467

H	-1.62646104	-3.88636380	2.04240832
H	-0.53466339	-2.71595128	1.27835905
H	-0.75488759	-4.32882906	0.54992667
H	2.07032286	-1.16674014	-5.02890445
H	3.65286498	-0.35299557	-5.04562366
H	2.15401525	0.59738236	-5.16838690
H	4.32759883	-1.05364562	1.99451509
H	4.18425969	-1.96651819	0.46935635
H	2.72298004	-1.36907519	1.30115215
H	2.19990800	1.10118617	1.36404602
H	3.27921514	2.26969943	0.56096230
H	3.79705921	1.43106944	2.04615480
H	5.85817895	0.64416493	0.94415922
H	5.43157553	1.43700445	-0.58942101
H	5.82665386	-0.30146915	-0.55540259
H	-0.33635746	2.25437269	-5.12534289
H	-1.79901461	3.25913410	-4.97279141
H	-1.92836699	1.48513796	-5.01558871
H	-1.08228754	5.45632550	-0.43512857
H	-1.95027926	5.41518512	1.11464042
H	-2.80307940	4.99842548	-0.38170017
H	-3.47454391	2.71645881	0.61128630
H	-2.68677764	3.24420854	2.12124658
H	-2.22693569	1.70002929	1.37791082
H	0.20078672	2.42033865	1.38075975
H	-0.23327545	3.98362693	2.09152278
H	0.68880959	3.92869421	0.56431789
H	0.00705922	0.02002729	-4.18782271

Table S36. H-only optimized geometry for molecule B of **1-Br**.

Fe	0.00000000	0.00000000	0.00000000
Br	-0.00285260	-0.26898655	2.37394913
N	-0.82067576	-1.20944550	-2.51304867
N	-0.99115877	-1.46689640	-1.16435957
N	1.45679081	-0.09683670	-2.46985244
N	1.75245992	-0.00000003	-1.13531789
N	-0.63368720	1.31930081	-2.46882338
N	-0.72016589	1.57848509	-1.11035626
C	-1.46407667	-2.17302999	-3.22991764
C	-2.05495718	-3.03439746	-2.35878956
C	-1.74534467	-2.56767235	-1.06939015
C	2.60927004	-0.14862476	-3.18142263
C	3.65378761	-0.07537329	-2.29750525
C	3.09620711	0.04485136	-1.02394355
C	-1.11104272	2.37049494	-3.16527269
C	-1.51479417	3.31817193	-2.25304165
C	-1.24368892	2.82243902	-0.99216142
C	-1.49856145	-2.15210459	-4.73096558
C	-2.19028919	-3.17628434	0.24492471
C	-3.06832669	-4.40069922	-0.00427351
C	-3.03227173	-2.12034161	1.00730725
C	-0.96814975	-3.58701522	1.06777426
C	2.64025820	-0.25853435	-4.66967886
C	3.80819046	0.18521672	0.30083838
C	3.71649072	-1.14865595	1.07872528
C	3.19721689	1.30961046	1.13531725
C	5.28251773	0.49589671	0.04003163
C	-1.14762895	2.41191014	-4.65939073
C	-1.47498495	3.47628337	0.35388145
C	-1.91621399	4.92274302	0.14810085
C	-2.57452399	2.70906867	1.10672884
C	-0.18128462	3.46535459	1.19302318
B	-0.00000016	0.00000002	-2.99029290
H	-2.64856859	-3.90377313	-2.61436678
H	4.70681938	-0.08089930	-2.55331044
H	-1.93868973	4.28658263	-2.49405134
H	-1.97927277	-1.23885586	-5.10963299
H	-2.06662419	-3.01576040	-5.09781390
H	-0.48936966	-2.19571301	-5.16433348
H	-3.96833467	-4.14518254	-0.58262203
H	-3.39545175	-4.81866738	0.95833179
H	-2.52300918	-5.18976235	-0.54334666
H	-2.43467723	-1.23209617	1.24652586
H	-3.39800696	-2.54143864	1.95560554
H	-3.89856552	-1.81110072	0.40496572

H	-0.38513192	-4.35462665	0.53684189
H	-1.28262479	-4.00454191	2.03620847
H	-0.31094917	-2.73504911	1.27578643
H	2.10463921	-1.15097680	-5.02318710
H	3.68020656	-0.32599577	-5.01188946
H	2.17689732	0.61323459	-5.15358649
H	2.67297019	-1.39384914	1.31946945
H	4.27236394	-1.07117681	2.02484175
H	4.14409786	-1.97246313	0.48957201
H	3.23344067	2.26800244	0.59638451
H	3.75414416	1.42336266	2.07706625
H	2.15353750	1.10146663	1.40054790
H	5.77931315	-0.31826209	-0.50739694
H	5.80962024	0.62063411	0.99673860
H	5.40422648	1.42453569	-0.53692490
H	-0.13787891	2.35679625	-5.09039913
H	-1.61084079	3.34945442	-4.98988377
H	-1.72683023	1.57527804	-5.07415508
H	-2.86138188	4.98591079	-0.41138066
H	-1.15566643	5.50398662	-0.39377113
H	-2.07587386	5.40444480	1.12324960
H	-2.79241939	3.20248208	2.06556557
H	-2.25853888	1.67938671	1.32540417
H	-3.50251269	2.67051055	0.51805304
H	0.63431676	3.98138242	0.66641869
H	0.14147240	2.44069918	1.41785909
H	-0.35473827	3.97523828	2.15173869
H	0.01282654	0.02088925	-4.18981983

Table S37. Fully optimized geometry for (+) isomer of **1-Br**.

Fe	0.00000000	0.00000000	0.00000000
Br	-0.04954199	-0.11234388	2.36349701
N	-0.74409599	-1.25416000	-2.48047414
N	-0.89168310	-1.50796693	-1.13684045
N	1.45832921	-0.01381884	-2.46486069
N	1.72219651	0.00000052	-1.11581618
N	-0.71432285	1.27186604	-2.46529604
N	-0.83197848	1.50634484	-1.11616051
C	-1.33367309	-2.23855359	-3.21075641
C	-1.87389798	-3.15092914	-2.31161018
C	-1.57941963	-2.66412036	-1.02167154
C	2.61999447	-0.02249184	-3.16464301
C	3.66265563	-0.01704444	-2.23856205
C	3.06590101	0.00122400	-0.96508707
C	-1.29135456	2.28099481	-3.16337300
C	-1.79740226	3.19144991	-2.23585694
C	-1.48985879	2.67779121	-0.96330601
C	-1.35099842	-2.25512410	-4.70294013
C	-1.93947914	-3.28533386	0.31206849
C	-2.72205175	-4.58921734	0.07931797
C	-2.82900187	-2.31537434	1.11545280
C	-0.65159741	-3.61813504	1.09300470
C	2.67910475	-0.03353514	-4.65562034
C	3.73731908	0.00983164	0.39150843
C	3.35884661	-1.27197051	1.16225556
C	3.29982740	1.26130755	1.17986146
C	5.26428884	0.04714442	0.21135423
C	-1.33076072	2.32849297	-4.65424734
C	-1.81069994	3.26383051	0.39507627
C	-2.54370281	4.60404156	0.21696586
C	-2.72688529	2.29494494	1.17014314
C	-0.50336308	3.51090917	1.17534401
B	0.00000000	0.00000000	-2.98152610
H	-2.41371311	-4.05563545	-2.56454336
H	4.72151677	-0.02332843	-2.46837176
H	-2.32156331	4.11177692	-2.46444598
H	-1.85439192	-1.36889960	-5.11583446
H	-1.88632435	-3.14621961	-5.05278697
H	-0.33551769	-2.27888616	-5.12431506
H	-2.98851321	-5.03388976	1.04852862
H	-2.12394150	-5.32604502	-0.47649371
H	-3.65390157	-4.40651919	-0.47543271
H	-3.75769626	-2.09925600	0.56763921
H	-2.31283151	-1.36908842	1.31688758
H	-3.09306566	-2.76119293	2.08586855

H	-0.90374889	-4.09532244	2.05165466
H	-0.06923988	-2.71588403	1.31664461
H	-0.01920027	-4.30880728	0.51653338
H	2.17225716	-0.91424049	-5.07508819
H	3.72594827	-0.05196012	-4.98185252
H	2.20010313	0.85639961	-5.08841871
H	3.85221202	-1.27988621	2.14531362
H	3.67615073	-2.16620654	0.60653634
H	2.27686615	-1.33257150	1.33388478
H	2.21720398	1.26625415	1.35835650
H	3.57096640	2.17730740	0.63547653
H	3.79563603	1.27943417	2.16145487
H	5.74947166	0.06035350	1.19735993
H	5.58446557	0.94665894	-0.33451608
H	5.62805764	-0.83758298	-0.33119006
H	-0.32061924	2.34503788	-5.08785570
H	-1.85807751	3.23342773	-4.97935265
H	-1.85239398	1.45689329	-5.07473367
H	-1.92691481	5.33188642	-0.33008917
H	-2.77482578	5.03129094	1.20287053
H	-3.49191317	4.47377405	-0.32454461
H	-3.66248769	2.12220890	0.61895206
H	-2.97544682	2.71618052	2.15539243
H	-2.23589278	1.32828075	1.33839668
H	0.04199599	2.57590217	1.35721743
H	-0.72886186	3.95722368	2.15500784
H	0.15380547	4.19810125	0.62313193
H	0.00544999	0.00755745	-4.18129443

Table S38. H-only optimized geometry for molecule A of **1-I**.

Fe	0.00000000	0.00000000	0.00000000
I	0.09280050	-0.27302386	2.58283877
N	-0.85271065	-1.19084915	-2.49078758
N	-1.12953526	-1.37650249	-1.15419160
N	1.45824422	-0.12048424	-2.46311553
N	1.75847892	0.00000039	-1.12388590
N	-0.63333759	1.31591622	-2.45776988
N	-0.66090237	1.60334132	-1.10397628
C	-1.54896374	-2.08735229	-3.23519596
C	-2.27000607	-2.87123524	-2.36246220
C	-1.98921519	-2.40294753	-1.07359370
C	2.58999684	-0.18337447	-3.19358523
C	3.65413804	-0.07977956	-2.31110954
C	3.10748272	0.03831913	-1.04755819
C	-1.21458739	2.31977564	-3.15430525
C	-1.59698508	3.29340087	-2.23469182
C	-1.23914785	2.81287682	-0.98328400
C	-1.47263504	-2.14983606	-4.72214417
C	-2.55310129	-2.91955870	0.23969240
C	-3.56900407	-4.03750673	-0.03232163
C	-3.26731113	-1.77513025	0.97929084
C	-1.42182942	-3.49689465	1.08889786
C	2.61493800	-0.30710461	-4.68253137
C	3.86290120	0.20155295	0.27080615
C	3.77632604	-1.10993802	1.08362845
C	3.28243342	1.36443451	1.07877361
C	5.34199137	0.51061095	-0.03192487
C	-1.35713835	2.31111863	-4.62739258
C	-1.39376181	3.50192912	0.36563799
C	-1.82731307	4.95357579	0.12592884
C	-2.45278515	2.79251438	1.19055765
C	-0.05722102	3.51520255	1.10391114
B	0.00000011	0.00000024	-2.97720916
H	-2.92846622	-3.68886532	-2.63121966
H	4.70481297	-0.08987657	-2.57504103
H	-2.07744439	4.23597347	-2.46773146
H	-1.82192421	-1.21854655	-5.19105275
H	-2.10038000	-2.97195966	-5.08717333
H	-0.44478265	-2.32393622	-5.07186764
H	-3.97444655	-4.40224154	0.92203735
H	-3.10257088	-4.89078398	-0.54597473
H	-4.41181008	-3.68238480	-0.64350418
H	-4.08662065	-1.36923077	0.36832711
H	-2.57484687	-0.95727266	1.21849602
H	-3.68712230	-2.14064994	1.92796712

H	-1.81119683	-3.84895673	2.05599690
H	-0.64664562	-2.75031771	1.29820267
H	-0.95067286	-4.34917632	0.57695418
H	2.06113051	-1.19186376	-5.02663500
H	3.65338921	-0.39761767	-5.02340474
H	2.16790457	0.56981077	-5.17269575
H	4.34260881	-1.01035506	2.02148937
H	4.19442409	-1.95074254	0.51151544
H	2.73528632	-1.34478833	1.34304848
H	2.23396833	1.19191058	1.34866448
H	3.34312579	2.30523309	0.51199850
H	3.84344216	1.48895132	2.01670713
H	5.88390867	0.66223604	0.91229449
H	5.44559849	1.42419878	-0.63485671
H	5.82973871	-0.31783094	-0.56582701
H	-0.38526326	2.21702332	-5.13258612
H	-1.82777567	3.24409139	-4.96063417
H	-1.98244843	1.47374970	-4.97057471
H	-1.09180202	5.49857086	-0.48321987
H	-1.92033887	5.47481262	1.08953595
H	-2.80425034	5.01076465	-0.37643144
H	-3.41715380	2.76559429	0.66234133
H	-2.60134756	3.30891162	2.15095105
H	-2.15622237	1.75820696	1.41760026
H	0.27985643	2.49835479	1.34468913
H	-0.15702012	4.05966657	2.05454320
H	0.72316441	4.00433458	0.50354072
H	0.00912076	0.01260340	-4.17630520

Table S39. H-only optimized geometry for molecule B of **1-I**.

Fe	0.00000000	0.00000000	0.00000000
I	0.03303971	-0.29842937	2.57805359
N	-0.82793341	-1.20262932	-2.49819628
N	-1.05130253	-1.43380231	-1.14481011
N	1.45918671	-0.10785720	-2.46616382
N	1.75970304	0.00000004	-1.12227662
N	-0.62113384	1.32008564	-2.45308157
N	-0.67491369	1.61099197	-1.09723744
C	-1.45495703	-2.14437066	-3.23232280
C	-2.10880690	-2.99606988	-2.35231529
C	-1.82740841	-2.52569332	-1.07560617
C	2.59584057	-0.17022800	-3.18234916
C	3.66095853	-0.08352394	-2.30025830
C	3.10862653	0.03387477	-1.02752579
C	-1.13485705	2.35940256	-3.14326329
C	-1.50119437	3.33881268	-2.24615752
C	-1.19344693	2.84377456	-0.97612067
C	-1.43848049	-2.15919626	-4.72449205
C	-2.33545144	-3.11086120	0.23253206
C	-3.26126116	-4.28764973	-0.05226585
C	-3.13501610	-2.02216001	0.99647542
C	-1.14430757	-3.59663849	1.07492755
C	2.63518684	-0.28915882	-4.67477215
C	3.83949910	0.19688322	0.29186520
C	3.76450293	-1.09852480	1.08026690
C	3.22503677	1.36083437	1.10760091
C	5.30504586	0.53189283	0.00197141
C	-1.20732266	2.37930259	-4.64017890
C	-1.38949401	3.53116340	0.36536674
C	-1.81095122	4.99115419	0.13436156
C	-2.46846407	2.79541135	1.17118181
C	-0.06255008	3.54418255	1.14538868
B	-0.00000011	0.00000000	-2.97050281
H	-2.71035206	-3.85521188	-2.62247025
H	4.71130464	-0.09399165	-2.56519394
H	-1.93380758	4.30140704	-2.49091300
H	-1.87913516	-1.24374662	-5.14505743
H	-2.01759682	-3.01626880	-5.08933432
H	-0.41740190	-2.24187994	-5.12390894
H	-4.13193020	-3.98632469	-0.65320949
H	-3.63564055	-4.69956522	0.89597885
H	-2.73836112	-5.09745736	-0.58239536
H	-2.50334061	-1.15928887	1.24047595
H	-3.52077708	-2.43179294	1.94147554
H	-3.98516536	-1.67468902	0.39167090

H	-0.61287169	-4.40825182	0.55567578
H	-1.49364379	-3.97833962	2.04583179
H	-0.43240816	-2.78800031	1.27290277
H	2.09962285	-1.18280938	-5.02516910
H	3.67752626	-0.36056271	-5.00769486
H	2.17571881	0.58100580	-5.16523566
H	2.72555659	-1.35833520	1.32665376
H	4.31559574	-1.00427969	2.02838535
H	4.20303101	-1.93133591	0.51061832
H	3.26633546	2.30097365	0.53913851
H	3.78575999	1.49335766	2.04426000
H	2.18163348	1.16030412	1.37596288
H	5.80969441	-0.28448155	-0.53548968
H	5.84307730	0.68501989	0.94854698
H	5.39950356	1.45059302	-0.59465948
H	-0.20751383	2.31567359	-5.09251666
H	-1.67713526	3.31257196	-4.97324036
H	-1.79768396	1.53788738	-5.02894966
H	-2.77246507	5.06102477	-0.39570647
H	-1.05418271	5.53873295	-0.44550816
H	-1.92757502	5.49709413	1.10310210
H	-2.64351257	3.30847775	2.12839656
H	-2.15846437	1.76546566	1.39767510
H	-3.41813620	2.75935929	0.61799002
H	0.72724181	4.04660782	0.56913139
H	0.27254191	2.52526299	1.37965961
H	-0.19259775	4.07622659	2.09901535
H	0.00709786	0.02163282	-4.17025723

Table S40. Fully optimized geometry for (+) isomer of **1-I**.

Fe	0.00000000	0.00000000	0.00000000
I	-0.04165183	-0.09230492	2.56484143
N	-0.74825550	-1.25196497	-2.47333274
N	-0.90247675	-1.51036989	-1.13064256
N	1.45851882	-0.01747993	-2.45934381
N	1.73313275	0.00000000	-1.11126675
N	-0.70931071	1.27497106	-2.45913799
N	-0.82600875	1.52239968	-1.11106926
C	-1.33944021	-2.22936384	-3.21084161
C	-1.88858204	-3.14287725	-2.31871499
C	-1.59793875	-2.66425449	-1.02543347
C	2.61314026	-0.02568163	-3.17000011
C	3.66389544	-0.01636211	-2.25403480
C	3.07976698	0.00386263	-0.97522094
C	-1.27805295	2.28079598	-3.16821667
C	-1.77864775	3.20352544	-2.25087439
C	-1.47643078	2.70129543	-0.97291531
C	-1.34948751	-2.24069706	-4.70315259
C	-1.97274158	-3.29439657	0.29979571
C	-2.75785088	-4.59408463	0.05158351
C	-2.86884147	-2.32715630	1.09864123
C	-0.69301071	-3.64054818	1.08774152
C	2.66015265	-0.04006437	-4.66142925
C	3.77360840	0.01517107	0.36972400
C	3.41281615	-1.26925842	1.14429311
C	3.34988443	1.26882000	1.16126531
C	5.29788071	0.05473550	0.16579064
C	-1.31539418	2.31528903	-4.65953436
C	-1.79945555	3.31215696	0.37376611
C	-2.51748677	4.65735785	0.17142336
C	-2.73364003	2.36614532	1.15499032
C	-0.49393845	3.56110221	1.15549757
B	0.00000000	0.00000000	-2.97106007
H	-2.43181601	-4.04361459	-2.57810360
H	4.72057456	-0.02160242	-2.49325874
H	-2.29588963	4.12525928	-2.48899301
H	-1.84775394	-1.35151961	-5.11569518
H	-1.88598572	-3.12893789	-5.05838012
H	-0.33192427	-2.26644815	-5.11915894
H	-3.03363816	-5.04388451	1.01582353
H	-2.15724852	-5.32934300	-0.50355064
H	-3.68463980	-4.40534353	-0.50955913
H	-3.80048855	-2.12381721	0.55082315
H	-2.36260666	-1.37346235	1.29023171
H	-3.12654223	-2.76616215	2.07376089

H	-0.95429736	-4.11146412	2.04693676
H	-0.09954105	-2.74545241	1.31047173
H	-0.06708352	-4.34029673	0.51515955
H	2.14927853	-0.92109335	-5.07519074
H	3.70449895	-0.06044324	-4.99551205
H	2.17907493	0.84949705	-5.09264022
H	3.90645914	-1.26985631	2.12711294
H	3.74114103	-2.15938665	0.58831124
H	2.33195073	-1.34529407	1.31577261
H	2.26738566	1.28617044	1.33779456
H	3.63134115	2.18238976	0.61789566
H	3.84482504	1.27991106	2.14331480
H	5.79723252	0.07002697	1.14465633
H	5.60824054	0.95440239	-0.38540043
H	5.65539232	-0.83008121	-0.38069820
H	-0.30466800	2.31695397	-5.09194752
H	-1.83259189	3.22303828	-4.99293891
H	-1.84603269	1.44584161	-5.07308155
H	-1.88985625	5.37085106	-0.38213525
H	-2.74930173	5.10077483	1.15000968
H	-3.46434624	4.52951289	-0.37295358
H	-3.67469751	2.21365674	0.60698373
H	-2.96860793	2.79323861	2.14094562
H	-2.26507625	1.38826858	1.32167167
H	0.05242832	2.62714220	1.33809771
H	-0.72000712	4.00818018	2.13459366
H	0.16293378	4.24763258	0.60200606
H	0.00390152	0.00743277	-4.17040323

References:

1. Jové, F. A.; Pariya, C.; Scoblete, M.; Yap, G. P. A.; Theopold, K. H., A Family of Four-Coordinate Iron(II) Complexes Bearing the Sterically Hindered Tris(pyrazolyl)borato Ligand $Tp^{tBu,Me}$. *Chem. Eur. J.* **2011,** *17*, 1310-1318.
2. Trofimenko, S.; Calabrese, J. C.; Kochi, J. K.; Wolowiec, S.; Hulsbergen, F. B.; Reedijk, J., Spectroscopic Analysis, Coordination Geometry, and X-ray Structures of Nickel(II) Compounds with Sterically Demanding Tris(pyrazolyl)borate Ligands and Azide or (Thio)cyanate Anions. Crystal and Molecular Structures of Bis[(μ-thiocyanato-*N*,*S*)(hydrotris(3-isopropyl-4-bromopyrazol-1-yl)borato)nickel(II)]-3-Heptane and (Thiocyanato-*N*)(hydrotris(3-*tert*-butyl-5-methylpyrazol-1-yl)borato)nickel(II). *Inorg. Chem.* **1992,** *31*, 3943-3950.
3. Sheldrick, G. M., A short history of *SHELX*. *Acta Crystallogr. A* **2008,** *64*, 112-122.
4. Sheldrick, G. M., *SHELXL-97, Program for crystal structure refinement*. University of Göttingen: Göttingen, Germany, 1997.
5. Dolomanov, O. V.; Bourhis, L. J.; Gildea, R. J.; Howard, J. A. K.; Puschmann, H., *OLEX*2: a complete structure solution, refinement and analysis program. *J. Appl. Crystallogr.* **2009,** *42*, 339-341.
6. Bain, G. A.; Berry, J. F., Diamagnetic Corrections and Pascal's Constants. *J. Chem. Educ.* **2008,** *85*, 532-536.
7. Bill, E. *mpView*, Version 1.4.6; Max Planck Institute for Chemical Energy Conversion.
8. Bill, E. *JulX20*, Version 1.4; Max Planck Institute for Chemical Energy Conversion.
9. Kahn, O., *Molecular Magnetism*. VCH Publishers, Inc.: 1993.
10. Hassan, A. K.; Pardi, L. A.; Krzystek, J.; Sienkiewicz, A.; Goy, P.; Rohrer, M.; Brunel, L. C., Ultrawide Band Multifrequency High-Field EMR Technique: A Methodology for Increasing Spectroscopic Information. *J. Magn. Reson.* **2000,** *142*, 300-312.
11. Bill, E. *mf2*, Version 2.7.3; Max Planck Institute for Chemical Energy Conversion.
12. Bill, E. *mx*, Version 1.8.4; Max Planck Institute for Chemical Energy Conversion.
13. Gütlich, P.; Bill, E.; Trautwein, A. X., *Mössbauer Spectroscopy and Transition Metal Chemistry: Fundamentals and Application*. Springer: 2011.
14. Taylor, J. R., *An Introduction to Error Analysis: The Study of Uncertainties in Physical Measurements.* 2nd ed.; University Science Books: 1997.
15. Neese, F., Quantum Chemistry and EPR Parameters. *eMagRes* **2017,** *6*, 1-22.
16. Tarrago, M.; Römelt, C.; Nehrkorn, J.; Schnegg, A.; Neese, F.; Bill, E.; Ye, S., Experimental and Theoretical Evidence for an Unusual Almost Triply Degenerate Electronic Ground State of Ferrous Tetraphenylporphyrin. *Inorg. Chem.* **2021,** *60*, 4966-4985.
17. Schäffer, C. E.; Jørgensen, C. K., The angular overlap model, an attempt to revive the ligand field approaches. *Mol. Phys.* **1965,** *9*, 401-412.
18. Schönherr, T.; Atanasov, M.; Adamsky, H., Angular Overlap Model. In *Comprehensive Coordination Chemistry II*, 2003; Vol. 2, pp 443-455.
19. Bersuker, I. B., Modern Aspects of the Jahn-Teller Effect Theory and Applications to Molecular Problems. *Chem. Rev.* **2001,** *101*, 1067-1114.
20. Neese, F., The ORCA program system. *WIREs Comput. Mol. Sci.* **2012,** *2*, 73-78.
21. Neese, F., Software update: The ORCA program system—version 5.0. *WIREs Comput. Mol. Sci.* **2022,** *12*, e1606.
22. Neese, F.; Wennmohs, F.; Aravena, D.; Atanasov, M.; Becker, U.; Bistoni, G.; Bykov, D.; Chilkuri, V. G.; Datta, D.; Dutta, A. K.; Ehlert, S.; Ganyushin, D.; Garcia, M.; Guo, Y.; Hansen, A.; Helmich-Paris, B.; Huntington, L.; Izsák, R.; Kettner, M.; Kollmar, C.; Kossmann,

S.; Krupička, M.; Lang, L.; Lechner, M.; Lenk, D.; Liakos, D. G.; Manganas, D.; Pantazis, D. A.; Papadopoulos, A.; Petrenko, T.; Pinski, P.; Pracht, P.; Reimann, C.; Retegan, M.; Riplinger, C.; Risthaus, T.; Roemelt, M.; Saitow, M.; Sandhöfer, B.; Schapiro, I.; Sen, A.; Sivalingam, K.; de Souza, B.; Stoychev, G.; Van den Heuvel, W.; Wezisla, B.; Kállay, M.; Grimme, S.; Valeev, E.; Chan, G.; Pittner, J.; Brehm, M.; Goerigk, L.; Åsgeirsson, V.; Unger, L., *ORCA Manual - Version 5.0.4*. 2023.
23. Grimme, S.; Antony, J.; Ehrlich, S.; Krieg, H., A consistent and accurate *ab initio* parametrization of density functional dispersion correction (DFT-D) for the 94 elements H-Pu. *J. Chem. Phys.* **2010,** *132*, 154104.
24. Grimme, S.; Ehrlich, S.; Goerigk, L., Effect of the Damping Function in Dispersion Corrected Density Functional Theory. *J. Comput. Chem.* **2011,** *32*, 1456-1465.
25. Becke, A. D., Density-functional exchange-energy approximation with correct asymptotic behavior. *Phys. Rev. A: Gen. Phys.* **1988,** *38*, 3098-3100.
26. Perdew, J. P., Density-functional approximation for the correlation energy of the inhomogeneous electron gas. *Phys. Rev. B* **1986,** *33*, 8822-8824.
27. Eichkorn, K.; Treutler, O.; Öhm, H.; Häser, M.; Ahlrichs, R., Auxiliary basis sets to approximate Coulomb potentials. *Chem. Phys. Lett.* **1995,** *240*, 283-290.
28. Eichkorn, K.; Weigend, F.; Treutler, O.; Ahlrichs, R., Auxiliary basis sets for main row atoms and transition metals and their use to approximate Coulomb potentials. *Theor. Chem. Acc.* **1997,** *97*, 119-124.
29. Weigend, F.; Ahlrichs, R., Balanced basis sets of split valence, triple zeta valence and quadruple zeta valence quality for H to Rn: Design and assessment of accuracy. *Phys. Chem. Chem. Phys.* **2005,** *7*, 3297-3305.
30. Weigend, F., Accurate Coulomb-fitting basis sets for H to Rn. *Phys. Chem. Chem. Phys.* **2006,** *8*, 1057-1065.
31. Lee, C.; Yang, W.; Parr, R. G., Development of the Colle-Salvetti correlation-energy formula into a functional of the electron density. *Phys. Rev. B: Condens. Matter Mater. Phys.* **1988,** *37*, 785-789.
32. Becke, A. D., Density functional thermochemistry. III. The role of exact exchange. *J. Chem. Phys.* **1993,** *98*, 5648-5652.
33. Douglas, M.; Kroll, N. M., Quantum Electrodynamical Corrections to the Fine Structure of Helium. *Ann. Phys.* **1974,** *82*, 89-155.
34. Hess, B. A., Applicability of the no-pair equation with free-particle projection operators to atomic and molecular structure calculations. *Phys. Rev. A: Gen. Phys.* **1985,** *32*, 756-763.
35. Hess, B. A., Relativistic electronic-structure calculations employing a two-component no-pair formalism with external-field projection operators. *Phys. Rev. A: Gen. Phys.* **1986,** *33*, 3742-3748.
36. Jansen, G.; Hess, B. A., Revision of the Douglas-Kroll transformation. *Phys. Rev. A: Gen. Phys.* **1989,** *39*, 6016-6017.
37. Samzow, R.; Hess, B. A.; Jansen, G., The two-electron terms of the no-pair Hamiltonian. *J. Chem. Phys.* **1992,** *96*, 1227-1231.
38. Pantazis, D. A.; Chen, X.-Y.; Landis, C. R.; Neese, F., All-Electron Scalar Relativistic Basis Sets for Third-Row Transition Metal Atoms. *J. Chem. Theory Comput.* **2008,** *4*, 908-919.
39. Pantazis, D. A.; Neese, F., All-Electron Scalar Relativistic Basis Sets for the Lanthanides. *J. Chem. Theory Comput.* **2009,** *5*, 2229-2238.

40. Pantazis, D. A.; Neese, F., All-Electron Scalar Relativistic Basis Sets for the Actinides. *J. Chem. Theory Comput.* **2011,** *7*, 677-684.
41. Pantazis, D. A.; Neese, F., All-electron scalar relativistic basis sets for the 6*p* elements. *Theor. Chem. Acc.* **2012,** *131*, 1292.
42. Rolfes, J. D.; Neese, F.; Pantazis, D. A., All-electron scalar relativistic basis sets for the elements Rb-Xe. *J. Comput. Chem.* **2020,** *41*, 1842-1849.
43. Neese, F.; Wennmohs, F.; Hansen, A.; Becker, U., Efficient, approximate and parallel Hartree–Fock and hybrid DFT calculations. A 'chain-of-spheres' algorithm for the Hartree–Fock exchange. *Chem. Phys.* **2009,** *356*, 98-109.
44. Hellweg, A.; Hättig, C.; Höfener, S.; Klopper, W., Optimized accurate auxiliary basis sets for RI-MP2 and RI-CC2 calculations for the atoms Rb to Rn. *Theor. Chem. Acc.* **2007,** *117*, 587-597.
45. Chmela, J.; Harding, M. E., Optimized auxiliary basis sets for density fitted post-Hartree–Fock calculations of lanthanide containing molecules. *Mol. Phys.* **2018,** *116*, 1523-1538.
46. Angeli, C.; Cimiraglia, R.; Evangelisti, S.; Leininger, T.; Malrieu, J.-P., Introduction of *n*-electron valence states for multireference perturbation theory. *J. Chem. Phys.* **2001,** *114*, 10252-10264.
47. Singh, S. K.; Atanasov, M.; Neese, F., Challenges in Multireference Perturbation Theory for the Calculations of the *g*-Tensor of First-Row Transition-Metal Complexes. *J. Chem. Theory Comput.* **2018,** *14*, 4662-4677.
48. Atanasov, M.; Ganyushin, D.; Pantazis, D. A.; Sivalingam, K.; Neese, F., Detailed Ab Initio First-Principles Study of the Magnetic Anisotropy in a Family of Trigonal Pyramidal Iron(II) Pyrrolide Complexes. *Inorg. Chem.* **2011,** *50*, 7460-7477.
49. Atanasov, M.; Aravena, D.; Suturina, E.; Bill, E.; Manganas, D.; Neese, F., First principles approach to the electronic structure, magnetic anisotropy and spin relaxation in mononuclear 3d-transition metal single molecule magnets. *Coord. Chem. Rev.* **2015,** *289-290*, 177-214.
50. Spiller, N.; Chilkuri, V. G.; DeBeer, S.; Neese, F., Sulfur vs. Selenium as Bridging Ligand in Di-Iron Complexes: A Theoretical Analysis. *Eur. J. Inorg. Chem.* **2020,** *2020*, 1525-1538.
51. Devkota, L.; SantaLucia, D. J.; Wheaton, A. M.; Pienkos, A. J.; Lindeman, S. V.; Krzystek, J.; Ozerov, M.; Berry, J. F.; Telser, J.; Fiedler, A. T., Spectroscopic and Magnetic Studies of Co(II) Scorpionate Complexes: Is There a Halide Effect on Magnetic Anisotropy? *Inorg. Chem.* **2023,** *62*, 5984-6002.
52. Ferrence, G. M.; Beitelman, A. D., Chloro[hydridotris(3-*tert*-butyl-5-methylpyrazolyl)borato]cobalt(II). *Acta Crystallogr. E* **2007,** *63*, m153-m155.
